# Developmental dynamic transcriptome and systematic analysis reveal the major genes underlying isoflavone accumulation in soybean

**DOI:** 10.3389/fpls.2023.1014349

**Published:** 2023-03-07

**Authors:** Heng Chen, Changkai Liu, Yansheng Li, Xue Wang, Xiangwen Pan, Feifei Wang, Qiuying Zhang

**Affiliations:** ^1^ Key Laboratory of Soybean Molecular Design and Breeding, Northeast Institute of Geography and Agroecology, Chinese Academy of Sciences, Harbin, China; ^2^ Innovation Academy for Seed Design, Chinese Academy of Sciences, Harbin, China; ^3^ College of Advanced Agricultural Sciences, University of Chinese Academy of Sciences, Beijing, China

**Keywords:** soy isoflavone, dynamic regulation networks, dynamic accumulation patterns, transcriptome, meta-analysis

## Abstract

**Introduction:**

Soy isoflavone, a class of polyphenolic compounds exclusively occurred in legumes, is an important bioactive compound for both plants and human beings. The outline of isoflavones biosynthesis pathway has been drawn up basically in the previous research. However, research on the subject has been mostly restricted to investigate the static regulation of isoflavone content in soybean, rather than characterize its dynamic variation and modulation network in developing seeds.

**Methods:**

In this study, by using eight recombinant inbred lines (RIL), the contents of six isoflavone components in the different stages of developing soybean seeds were determined to characterize the dynamic variation of isoflavones, and the isoflavones accumulation pattern at physiological level was investigated. Meanwhile, we integrated and analyzed the whole genome expression profile of four lines and 42 meta-transcriptome data, based on the multiple algorithms.

**Results:**

This study: 1) obtained 4 molecular modules strongly correlated with isoflavone accumulation; 2) identified 28 novel major genes that could affect the accumulation of isoflavones in developing seeds free from the limitation of environments; 3) discussed the dynamic molecular patterns regulating isoflavones accumulation in developing seed; 4) expanded the isoflavone biosynthesis pathway.

**Discussion:**

The results not only promote the understandings on the biosynthesis and regulation of isoflavones at physiological and molecular level, but also facilitate to breed elite soybean cultivars with high isoflavone contents.

## Graphical Abstract

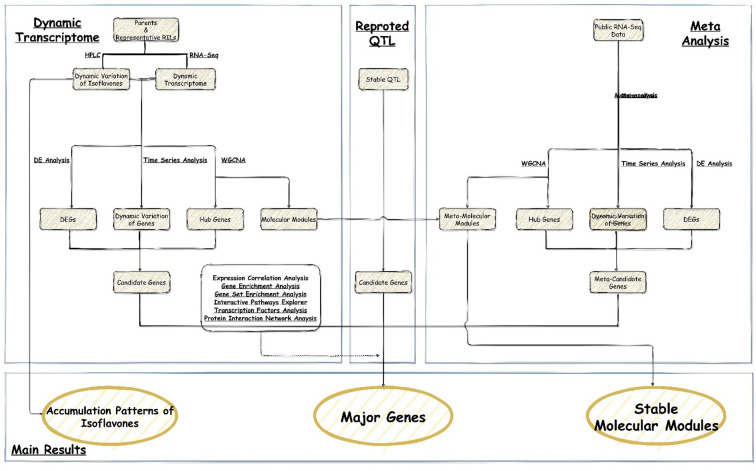

## 1 Introduction

Soy isoflavone, a sub–group of flavonoids derived from phenylalanine, is a class of polyphenolic compounds exclusively occurred in legumes. This secondary metabolite is usually referred as phytoestrogen, since it is structurally and functionally similar to estrogen. Currently, isoflavones have been widely used in health product and bio–pharmaceutical, for it has the functions of preventing breast cancer, relieving Alzheimer disease, resisting menopause syndrome, et cetera (Dixon, 2004). Isoflavones also play a vital role in the system of plant defense responses. It could be further synthesized into major phytoalexins, and therefore exhibiting significant effect in plant insect–resistance, disease–resistance, and various other biotic stresses (Graham et al., 2007).

Because of the irreplaceable role of isoflavone in human health and plant growth, many related studies have been carried out, including its structure, ingredient, and genetic background. Up to now, a total of 12 kind of isoflavones are found, which are generally divided into 4 main categories based on their structure. They are: 1) aglycones (including daidzein, glycitein and genistein); 2) glycosides (including daidzin, glycitin and genistin); 3) acetyl–glycosides (including acetyl-daidzin, acetyl-glycitin and acetyl-genistin); 4) malonyl–glycosides (including malonyl-daidzin, malonyl-glycitin and malonyl-genistin) (Dixon, 2004). The glucosides are the hinges of isoflavone biosynthesis pathway *in vivo*, for they are the products of aglycones and the precursors of other two categories (Yu et al., 2003). Since the aglycones are the mainly active form in soybean seed while the glycosides are the primary storage form in soybean seed, the aglycones and glycosides are the most important concerned categories among these 12 isoflavones (Chen et al., 2021).

Isoflavone content is a typical quantitative trait, which is regulated by both genetic and environmental factors. The approaches of quantitative genetics have been the major methods for investigating the genetic background of isoflavone, such as QTL mapping. Based on the high-density genetic map, a total of 63 stable QTL (containing 52 meta-QTL and 11 novel QTL) were mapped by our proceeding study (Chen et al., 2021). However, although these QTL have been narrowed down to a short interval length, major genes influencing the accumulation of isoflavones are required for the quantitative trait genes (QTG), rather than QTL, regulate the phenotype *in vivo*.

Transcriptome analysis is a good way to obtain QTG from QTL, as well as an efficient and useful approach in exploring the major genes of complex quantitative trait (Stark et al., 2019). For instance, *CHS7* and *cytochrome P450* have been identified separately by Dhaubhadel et al. (2007) and (Guttikonda et al., 2010), which influenced the accumulation of isoflavone. Generally, candidate genes could be obtained from RNA–seq data by differential gene expression analysis (DE analysis). By comparing the transcriptional changes, the differential expression genes (DEG) among several significantly different samples could be obtained. However, the internal relationships between candidate genes could not be clarified in this way. The weighted gene co–expression network analysis (WGCNA) to make up the inadequacy of DE analysis, for it could assist to describe the internal relationships among candidate genes by analyzing the degree of association between genes, finding molecular modules (clusters) and mapping hub genes (Zhao et al., 2010). The time series analysis is also applied to analyze RNA–seq data, which generally require a time series expression profile and could give the temporal expression variation of several important clusters or genes.

The outline of isoflavones biosynthesis pathway has been basically revealed for decades. However, the accumulation pattern in different developing stages of isoflavones at both molecular and physiological level is still unclear. Actually, there is a significant difference between the variation trend of different components, and a quite close internal relationship among different components of isoflavone (D’Agostina et al., 2008). The main factor influencing isoflavones variation might not be same in different developing stages. Consequently, clarifying the accumulation patterns of isoflavone components at physiological and molecular level is quite essential for comprehensively and deeply understanding the biosynthesis and regulation mechanisms of isoflavone in soybean.

The aims of current study were: 1) to investigate the accumulation patterns of isoflavones at physiological and molecular level; 2) to map the major genes and QTG associated with isoflavones accumulation; 3) to explore the dynamic regulation network regulating isoflavones accumulation in developing seed. The results could facilitate elucidating the molecular mechanism of isoflavones biosynthesis and regulation, as well as breeding elite soybean cultivars with high isoflavone content.

## 2 Materials and methods

### 2.1 Materials and field experiment

#### 2.1.1 Materials for characterizing the phenotype dynamic variation

**Figure 1:**
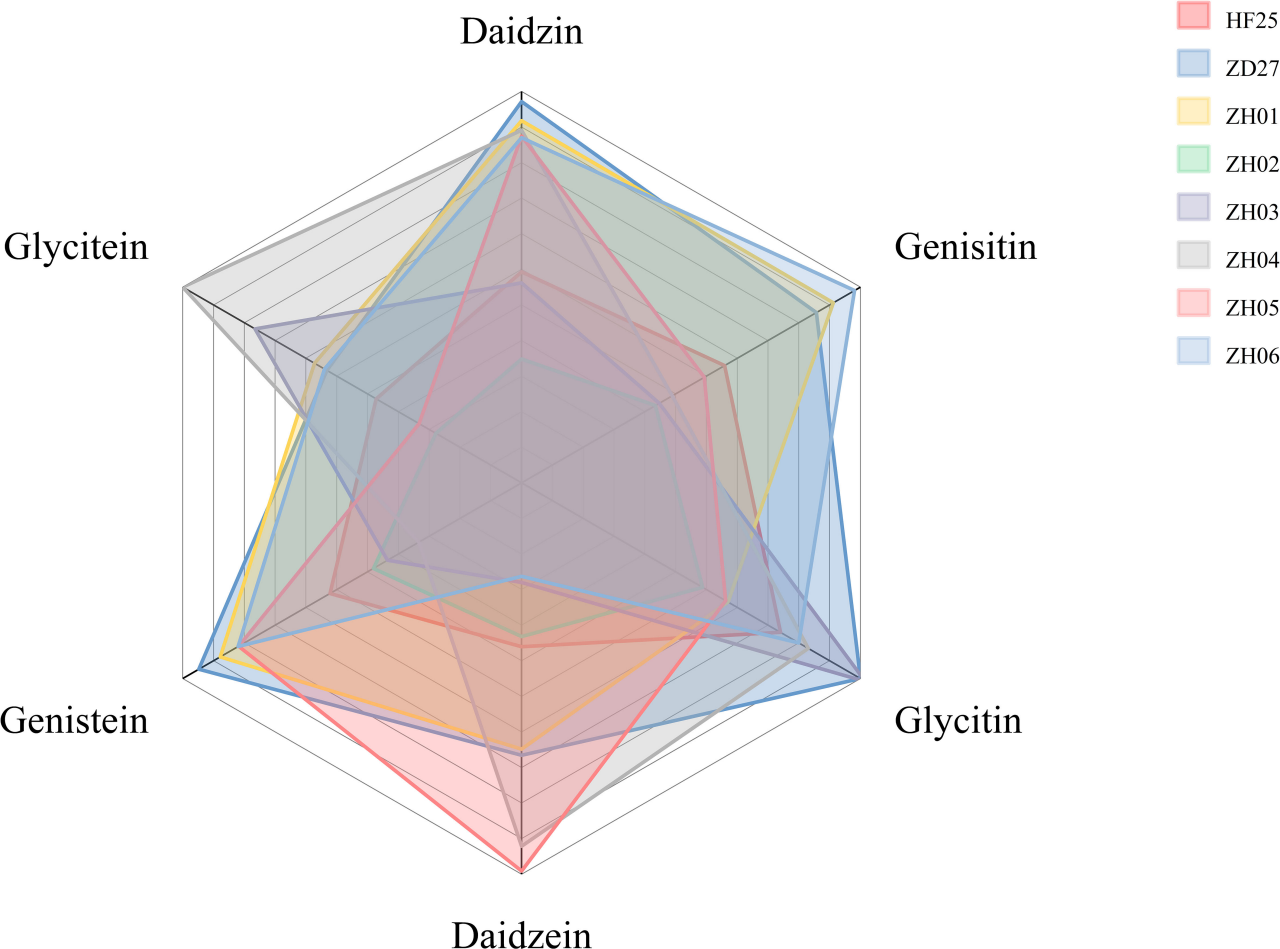
The isoflavone content of samples in this study (The distance between central point and vertex of each color block represents the content of isoflavone component in a sample).

To construct the materials for characterizing the dynamic variation of six isoflavone components, the parental lines and six representative lines of a RIL population (**Figure 1**) constructed by our research group were adopted (Chen et al., 2021). Briefly, by using the single–seed descendent method, a F7 population named as ZH RIL with 162 lines was obtained based on a pair of parents, ‘Zhongdou 27’ (ZD27) and ‘Hefeng 25’ (HF25).

The two cultivars exhibit significant difference at phenotype level: ‘Zhongdou 27’ containing high isoflavone contents, a late–maturing cultivar, mainly planted in Huang–Huai–Hai region of China (Northern China); while ‘Hefeng 25’ containing low isoflavone contents, a higher–yielding cultivar, is mainly planted in Northeast China. Meanwhile, the six selected lines are also significantly different from each other at phenotypic level, as shown by the results of isoflavone determination (**Figure 1**). All of these materials were planted in the Acheng district, Harbin, Heilongjiang province, China (45.6°N, 126.6°E) in 2021. The main materials information could be found in **Figure 1** and **Table 1**.

**Table 1:** Basic information of filed experiments.

Experiments	Samples	Periods	Field experiment locations
**Dynamic variation of isoflavones**	ZD27 HF25 ZH01 ZH02 ZH03 ZH04 ZH05 ZH06	S1-S10	Acheng district, Harbin
**RNA-seq**	ZD27 HF25 ZH01 ZH02	S2-S8	Acheng district, Harbin
**qRT-PCR**	ZD27 HF25 ZH01 ZH02	S2-S8	Acheng district, Harbin

For characterizing the complete process of isoflavone accumulation during seed development, we selected 10 representative periods (S1-S10) in this part (**Figure 1** and **Table 1**). Samples at S1 period were collected at R5 of soybean developing stage, while samples at S10 period were at full maturity stage. Each interval period was 7 days.

#### 2.1.2 Materials for RNA-seq and q-RTPCR

To construct the materials for RNA–seq and qRT–PCR analysis, the parents (ZD27 and HF25) and two representative lines (ZH01 and ZH02) of the RIL population were adopted. They were planted in the Acheng district, Harbin, Heilongjiang province, China. According to the time series, 7 groups (S2-S8) of soybean seeds were used for RNA-seq (**Figure 1** and **Table 1**).

#### 2.1.3 Field experiment and sample collection

These materials were sown in rows with 3 m long, 0.65 m wide and a distance with 0.08 m between the individual plants. Field management followed normal soybean production practices. The main information of the field experiment design could be found in **Figure 1** and **Table 1**.

Soybean seeds for RNA-seq were collected into pre–cooling and RNase–free cryogenic vials (Biosharp, Anhui, China), and then quickly frozen and stored at -80 °C condition. Soybean seeds for isoflavone determination were treated by the following steps: 1) green removed at 105 °C for 30 mins; 2) dried at 45 °C for 48 hours; 3) stored at -20 °C condition.

### 2.2 Phenotype identification

To determine the contents of six isoflavones in soybean seed, the high–performance liquid chromatography (HPLC) method was utilized, which is one of the most effective and common approach for isolating and determining complex metabolites. The detailed operations and conditions of HPLC assay were described in our previous paper (Chen et al., 2021).

### 2.3 RNA-seq and quality control

To obtain the whole genome expression profile of the developing seeds, we performed the following steps in turn: total RNA isolation, RNA sequencing and *de novo* transcriptome assembly. Several kits, programs and instruments were also utilized, whose detailed operations could be acquired on the tutorials or instructions of manufacturer.

For the isolation of total RNA, the Ultrapure RNA Kit (ComWin Biotech, Beijing, China) was used. The extracting solution of total RNA was stored at -80 °C condition. RNA quality was checked and evaluated by the Agilent 2100 Nano Bioanalyzer (Agilent Technologies, California, USA) and NanoDrop 2000 spectrophotometer (Thermo Fisher Scientific, Massachusetts, USA). The total RNA extraction was permitted to construct cDNA library, if and only if it could meet the criteria as follow: 1) RNA content ≥ 1.0 µg and RNA concentration ≥ 35 ng/µl; 2) OD260/OD230 ≥ 1.0 and OD260/280 ≥ 1.8; 3) RNA Integrity Number (RIN) ≥ 7.

For RNA sequencing, the Illumina Truseq™ RNA sample prep Kit (NEB, Massachusetts, USA) and Illumina NovaSeq 6000 platform (Illumina, California, USA) was used to perform RNA paired–end (PE) sequencing. Then, the BCL convert (Illumina, California, USA) was used to transform the sequence results to raw reads. The Phred Score (QPhred) and base distribution were selected to evaluate the quality of RNA sequencing. The formula of Phred Score is:


$$QPhred=-10\;\log_{10}e$$

where the *e* represents the error rate of base sequencing.

For *de novo* transcriptome assembly, StringTie2 (Kovaka et al., 2019) was utilized. The gffcompare, a subassembly of StringTie2, was also used to discover new transcripts, genes and/or exons based on the reference genome Williams 82. a4. v1 (Schmutz et al., 2010) from Phytozome v13 (Goodstein et al., 2011).

### 2.4 Transcriptome analysis

To further analyze the whole genome expression profile of developing seeds, multiple algorithms were applied to characterize the clusters and genes associated with isoflavone accumulation. Several sorts of software were involved, whose specific operation could be found in their tutorials or references.

#### 2.4.1 Whole genome expression quantification and analysis

For quantifying the whole genome expression, the program Salmon (Patro et al., 2017) was used. The transcripts per million reads (TPM) was selected as standards to evaluate the expression level, whose formula is as follows:


$$TPM=\frac{R\times 10^{6}}{\left(\frac{R_{1}}{l_{1}}+\frac{R_{2}}{l_{2}}+\cdots +\frac{R_{n}}{l_{n}}\times l\right)}$$

where the *R* and *l* represents the read counts and length of the analyzed gene, the *R_1_* to *R_n_* represents the read counts of the *1st* gene to *nth* gene, the *l_1_* to *l_n_* represents the gene length of *1st* gene to *nth* gene.

For analyzing the variation of whole genome expression between different biological repetitions of a single sample, we performed the correlation analysis with Pearson correlation coefficient. The result provides a reference for DE analysis as well. Additionally, for characterizing and investigating the variation of whole genome expression among different samples, three algorithms were utilized, including Venn analysis and correlation analysis (with Pearson correlation coefficient).

#### 2.4.2 Selected algorithms

For detecting the DEGs among different samples, DE analysis was performed with the raw read counts *via* the R package DEseq2 (Love et al., 2014). The parameters set for this algorithm were *FDR q – val< 0.05* and |log_2_ *FC*| ≥ 1 (fold change). Moreover, for obtaining the gene clusters and hub genes correlated with isoflavones accumulation, the R package WGCNA (Zhang and Horvath, 2005) was utilized.

For analyzing the dynamic variation of transcript profile, two programs, the maSigPro (Nueda et al., 2014) and Short Time–series Expression Miner (STEM) (Ernst and Bar-Joseph, 2006), were respectively applied to the temporal expression differences and temporal expression trends during seeds development. In this process, we selected 6 reported genes as standard to screen major genes of isoflavone accumulation. Their expression variation in developing seeds was characterized based on RNA-seq data of this study (**Figure S1**).

For further analysis and selection of the candidate genes based on their properties and functions, several other databases and related procedures were also applied, including Plant Transcription Factor Database V5.0 (PlantTFDB) (Tian et al., 2019) and STRING V11 (Szklarczyk et al., 2018).

For annotating and analyzing the functions of candidate genes, the gene functional annotation and enrichment analysis were used in the study. In this process, the R package ClusterProfiler (Yu et al., 2012) was used in KEEG and GO enrichment analysis, which was based on the Fisher’s precision probability test with *P – adjust >* 0.05.

### 2.5 Meta-analysis

To collect and integrate the RNA-seq data from the previous studies, the meta–analysis (Borenstein et al., 2009) was used. The basic information of meta-data collected in this study were: cultivars’ name, period of seed development, experiment time, experimental conditions, sequencing techniques, sequencing platform, library layout, treatment and source (accession number and reference).

Approximately 700 Gbp meta-data were collected from the National Center of Biotechnology Information (NCBI) and National Genomics Data Center (NGDC), including whole genome expression profiles of 80 developing soybean seeds. The transcriptome data was divided into 4 groups, whose main information was as shown in **Table 2** and **Table S1**. To be specific, the materials of group 1 were formed by ‘Suinong 14’ (SN14) and its four (chromosome segment substitution lines) CSSLs: SN14a, SN14b, SN14c and SN14d. Each germplasm contained samples with 3 stages: S1 (early maturation), S2 (middle maturation) and S3 (dry seed). The samples of group 2 consisted of ‘Williams 82’ (Wm82). Each cultivar in group 2 contained samples with 4 stages: S1 (early maturation), S2 (middle maturation), S3 (late maturation) and S4 (dry seed). The samples of group 3 included Dongnong 47 (DN47) and its near–isogenic line (DN47n), and each cultivar comprised seeds with 5 stages (days after flowering, DAF): S1 (18 DAF), S2 (25 DAF), S3 (35 DAF), S4 (50 DAF) and S5 (55 DAF). The group 4 was formed by 2 RIL derived from V99–5089 and CX–1834, VC–M and VC–m, and these materials involved 5 periods: S1 (2 – 4 mm seed), S2 (4 – 6 mm seed), S3 (6 – 8 mm seed), S4 (8 – 10 mm seed) and S5 (10 – 12 mm seed).

**Table 2:** The basic information of Meta–data collected in this study.

Group	Germplasms	Number of Sample	Meta–data Size	Platform	Accession and Reference
**G1**	SN14 SN14a SN14b SN14c SN14d	45	355 Gbp	Illumina HiSeq 4000	PRJCA000523 (Qi et al., 2018)
**G2**	Wm82	12	9 Gbp	Illumina HiSeq 2000	PRJNA388955 (Pelletier et al., 2017)
**G3**	DN47 DN47n	20	211 Gbp	Illumina Hiseq 2000	PRJNA315512 (Song et al., 2016)
**G4**	VC–M VC–m	30	118 Gbp	Illumina Hiseq 2000	PRJNA304631 (Redekar et al., 2015)

The collected raw data were treated by the following steps: 1) quantifying the data by using Salmon software and TPM algorithm; 2) performing multiple algorithms in transcriptome analysis, including DE analysis, WGCNA and time series analysis; 3) performing gene functional annotation and enrichment analysis. For simplifying the analysis process in this part, the following conditions and requirements were set: 1) the Williams 82.a4.v1 was selected as the reference genome; 2) the expression abundance of *IFS1* (*Glyma.07G202300*) and *CHS7* (*Glyma.01G228700*) were used to define the strength of isoflavone accumulation; 3) the top 5000 genes with maximum mean absolute deviation (MAD) of each meta-data group were used to perform WGCNA; 4) the gene cluster generated by WGCNA were selected with the combination with metabolic pathway analysis by iPath; 5) the gene clusters number of time series analysis was set as nine.

### 2.6 Linking candidate genes with isoflavone-related QTL

To further screen the candidate genes, the association analysis between the candidate genes and QTL was performed. Based on the 63 stable QTL (52 meta-QTL and 11 novel QTL) mapped in our previous study (**Table S2**) (Chen et al., 2021), the genes acquired from the transcriptome analysis and meta-analysis were selected through the co-localization with these QTL to obtain QTG underlying the accumulation of isoflavone. The results could further understand the relationship between the candidate genes and isoflavone components.

### 2.7 qRT-PCR

To validate the reliability of RNA–seq and select the major genes obtained from transcriptome analysis and meta–analysis, the qRT–PCR analysis was conducted based on a LightCycler 480II platform (Roche, Basel, Switzerland) with MagicSYBR Mixture (ComWin Biotech, Beijing, China). The total RNA was used to construct cDNA library immediately by HiFiScript cDNA Synthesis Kit (ComWin Biotech, Beijing, China)., and then stored at -20 °C condition. The primers were listed in **Table S3**, and the PCR amplification conditions were set according to **Table S4**. The internal control was *GmUKN1* (*Glyma.12G020500*) (Hu et al., 2009). Three independent experiments were executed for each sample. The relative abundance of gene expression was estimated *via* the underlying comparative threshold method (2^-ΔΔ^ *^CT^*) (Pfaffl, 2001).

## 3 Results

### 3.1 Dynamic variation of isoflavone components

**Figure 2:**
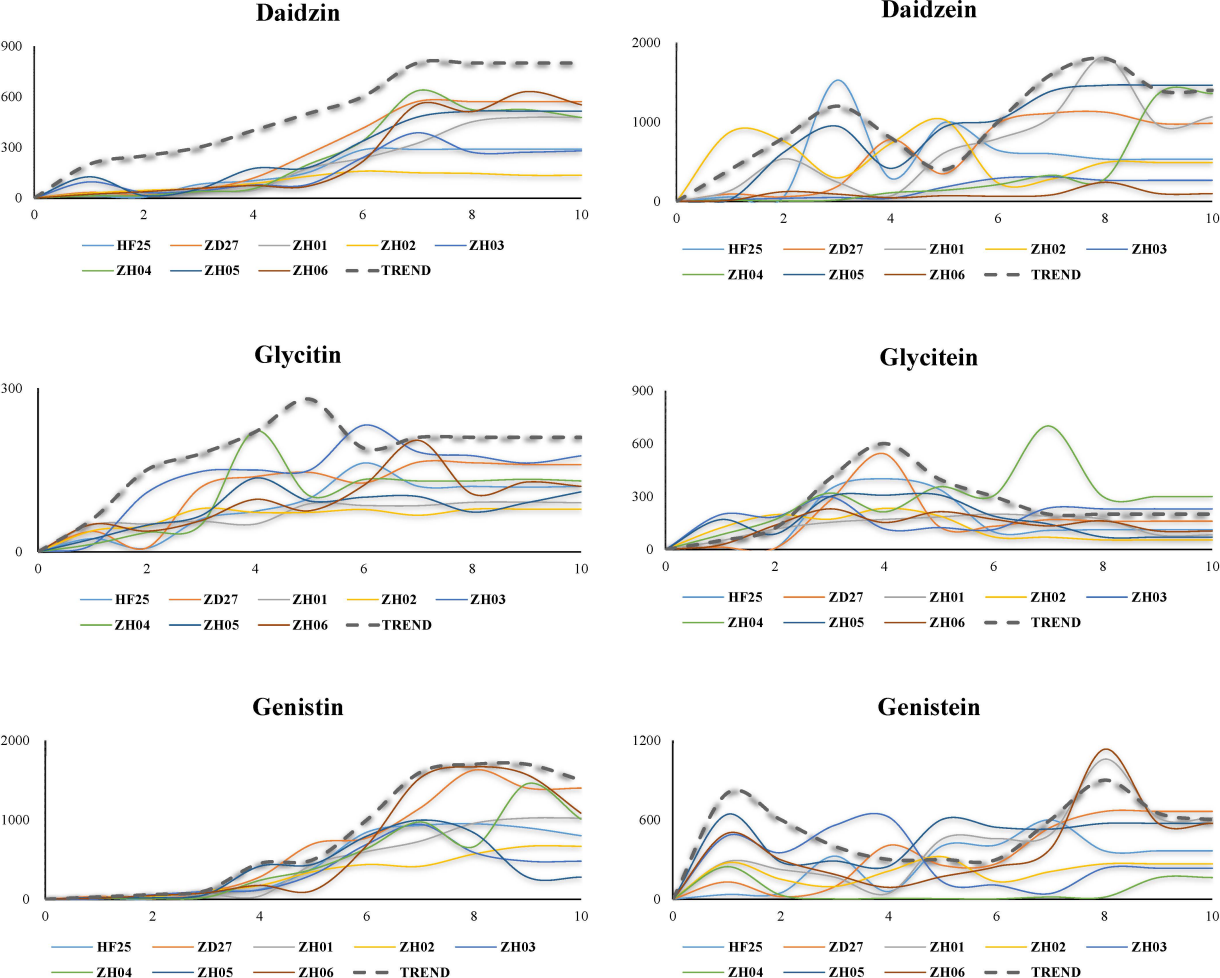
Dynamic variation of six isoflavone compounds in developing soybean seed (Each broken line represents the variation trend of isoflavone content, while each full line represents the real situation of the change of isoflavone content).

The accumulation patterns and overall trends of each isoflavone contents in developing seeds were summarized in **Figure 2**.

The accumulation pattern of daidzin is “climbing mode”: rising gently and then reaching equilibrium. Specifically, the accumulation of daidzin started during S1-S2, increased rapidly at S4, and reached equilibrium at S7 or S8.

The accumulation pattern of daidzein is “two peaks mode”, which could be divided into 3 phases: 1) rising and then decreasing (S1-S5); 2) rising and decreasing again (S5-S8); 3) reaching equilibrium (S9-S10). The first peak value was occurred at S2 or S3, while the second peak value was usually occurred at S7 or S8.

The accumulation pattern of glycitin is “single peak and climbing mode”, which is similar to pattern of daidzin accumulation but have a peak at S5. The glycitin contents reached its equilibrium at S6 or S7 (except ZH02 which reached its equilibrium at S3), which was notably earlier than other isoflavone components.

The accumulation pattern of glycitein is “single peak mode”: 1) increased during S1-S4; 2) decreased slowly during S4-S7; 3) kept constant till the end. Of course, there were some exceptions. The variation of glyceitin contents in ZD27 started at S2 and ended at S5, which exhibited a short and early variation process; while ZH04 had a long and late accumulation process which reached the peak at S7 and ended at S8.

The accumulation pattern of genistin is “two-part climbing mode”. In the first part (S1-S4): the contents of genistin were unchanged during S1-S3, and then increased slowly during S3-S4. In the second part (S5-S10): genistin contents increased at a high rate during S5-S7, and stopped increasing and maintained constant during S7-S9, and declined lightly during S9-S10. Two significant characteristics were suggested: 1) the difference of genistin contents among different lines firstly appeared at S4; 2) the genistin contents of matured seed were depended on the genistin biosynthesis rate during S5-S7.

The accumulation pattern of genistein is “bipolar peak mode”, which exhibits a special variation model compared with other isoflavones. The pattern could be separated to two parts: 1) S1-S5 (the first peak appeared at S1 or S2, and then decreased till S5); 2) S5-S10 (the genistein restarted to accumulate at S5, and reached second peak at S8, and then decreased lightly and kept constant).

### 3.2 Transcriptome analysis

#### 3.2.1 RNA–seq evaluation

Based on the Illumina NovaSeq 6000 platform, this study obtained 444.5G clean reads (with a Q30 ratio of more than 90%) from the 56 soybean germplasms (**Table S5**). The results of quality control suggested a high quality of sequencing data (**Table S5** and **Figure S2A**), and the comparative analysis of data indicated that the samples of RNA-seq were highly pure and the reads were appropriate to gene assembly (**Figure S2B**). Evaluating the quality of sequencing data indicated that: 1) the gene annotation is quite complete (**Figure S2C**); 2) the coverage of sequencing was great and homogeneous (**Figure S2D**). Therefore, the high-quality RNA–seq data were obtained.

**Figure 3:**
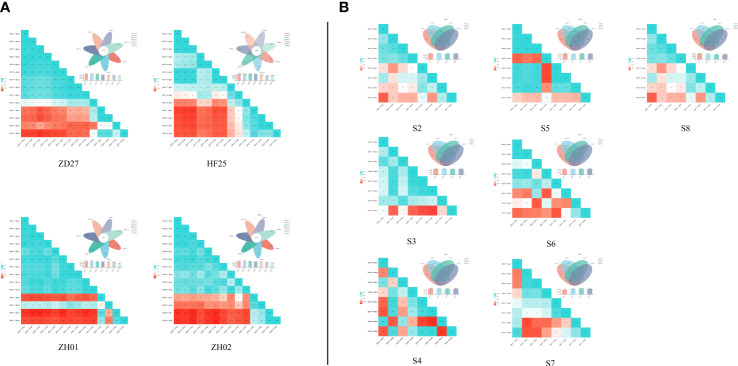
Venn analysis and correlation analysis among the RNA-seq of different samples **(A)** Venn analysis and correlation analysis of horizontal comparison; **(B)** Venn analysis and correlation analysis of lengthways comparison.

By transcript assembly and gene functional annotation, the current research obtained: 1) 118347 transcripts and 50556 genes (**Table S6**). The expression amount of these genes and transcripts were quantified by Salmon (**Figure S2E**) (Patro et al., 2017). This indicates that the samples and their transcriptome data could characterize the development of soybean seeds in whole. Several specifically expressed and co-expressed genes were acquired, by the Venn analysis among the different stages of the same germplasm and among the different germplasms in the common stage (**Figure 3**). Moreover, the expression amounts of genes exhibited consistency with qRT-PCR results of selected genes in this study, indicating a strong reliability of RNA-seq results (**Figure S3**). All in all, the transcript profile of this research is strongly reliable, repeatable and representative, as well as suitable for further analysis.

#### 3.2.2 DE analysis

From the perspective of germplasm and period, the RNA-seq data of 56 soybean samples were divided into 11 groups (4 germplasm groups and 7 period groups) for DE analysis (**Figure S4**). Among them, 11 times of DE analysis were carried out for each germplasm group, and 6 times of DE analysis were carried out for each period group.

In ZH01 group, 36571 DEGs were detected, including 17228 up-regulated genes and 19343 down-regulated genes. In ZH02 group, 76744 DEGs were generated, including 36051 up-regulated genes and 40693 down-regulated genes. In ZD27 group, 36773 DEGs were detected, including 16679 up-regulated genes and 20094 down-regulated genes. In HF25 group, approximately 41000 DEGs were obtained, including about 20000 up-regulated genes and 21000 down-regulated genes (**Figure S4**). Additionally, we also detected 4286 DEGs in S2 group, 3231 DEGs in S3 group, 5536 DEGs in S4 group, 11542 DEGs in S5 group, 36 DEGs in S6 group, 12338 DEGs in S7 group and 1999 DEGs in S8 group (**Figure S4**).

#### 3.2.3 DEGs related to the dynamic variation of isoflavone components

According to the variation trend of daidzin content in the developing seeds, the research suggests that the related main genes should be significantly differential expression in S3-S4 (the beginning of daidzin accumulation) and S6-S7 (the end of daidzin accumulation) period, which may be slightly different in various lines. Thus, the DEGs of 8 comparison groups were selected to examine the genes modulating daidzin accumulation, as shown in **Table S7**. The results of gene functional annotation showed that 122 genes may be closely related to the accumulation of daidzin. Comparing the expression of these 122 genes in different samples at S4 (the daidzin content began to differ among each sample) and S7 (the daidzin is close to the end of accumulation in ZD27, and is still accumulating in ZH01, but stops accumulating in HF25 and ZH02). This indicates that the 38 genes of S4 stage should play an important role in the initial stage of daidzin accumulation, while the 61 genes of S7 stage should influence the terminal stage of daidzin accumulation (**Table S7**). A total of 23 genes functionally related to daidzin accumulation were significantly differentially expressed in both initial and terminal stage, indicating that these genes might be involved in the whole process of daidzin accumulation. Briefly, the above results suggest that: 1) 15 genes significantly affect the initiation of daidzin accumulation; 2) 38 genes are involved in the termination of daidzin accumulation; 3) 23 genes are involved in the whole process of daidzin accumulation.

Based on the variation trend of daidzein content, the current study suggests that the daidzein related major genes are significantly differentially expressed in S2-S3 (the rising section of the first peak of daidzein accumulation), S3-S4 (the falling section of the first peak of daidzin accumulation), S6-S7 (the rising section of the second peak of daidzein accumulation) and S7-S8 period (the end of daidzein accumulation). According to the above characteristics, the DEGs of 19 comparison groups were selected (**Table S7**). With the combination of gene functional annotation and gene enrichment analysis, a total of 198 DEGs were obtained. *Via* further comparing these genes with the DEGs obtained from lengthways comparison (S2, S4, S6 and S7 were selected), we found 132 candidate genes involved in the accumulation of daidzein (**Table S7**), including: 1) 38 genes significantly affecting the initial accumulation of daidzein at the first stage; 2) 59 genes involved in the termination of daidzein accumulation at first stage; 3) 20 genes involved in the whole process of daidzein accumulation at first stage; 4) 48 genes significantly affecting the initiation of daidzein accumulation at the second stage; 5) 79 genes involved in the termination of daidzein accumulation at the second stage; 6) 24 genes involved in the whole process of daidzein accumulation at the second stage; 7) 10 genes involved in the whole process of daidzein accumulation.

Combining with the change trend of glycitin content and the results of DE analysis, the glycitin related genes were significantly differentially expressed in S2-S3 (rising stage of glycitin accumulation) and S5-S6 (falling stage of glycitin accumulation), with slight difference in various soybean germplasms. From the above perspective, DEGs of 6 comparison groups were used for researching the major genes of glycitin accumulation (**Table S7**). Screening with gene functional annotation, 155 genes were obtained. The differential expression of these genes in different materials at the same period (S2, S4 and S5 were selected) indicates that 119 genes are involved in the accumulation of glycin during the development of soybean seed (**Table S7**), including: 1) 36 genes involved in the beginning of the increase stage of glycin content; 2) 44 genes involved in the termination of increase stage and the beginning of decrease stage; 3) 106 genes involved in the termination of the decline stage; 4) 17 genes involved in the whole increasing process; 5) 36 genes involved in the whole decline process; 6) 16 genes involved in the whole process of glycitin accumulation.

Due to the dynamic variation of glycitein content is “single peak mode”, we argue that the major genes of glycitein accumulation should be signally varied in S2-S4 (rising stage of glycitein accumulation) and S4-S7 (falling stage of glycitein accumulation). Consequently, DEGs from 9 lengthways comparison groups were utilized to screen candidate genes (**Table S7**). The results manifest that 156 genes are closely associated with the accumulation of glycitein. *Via* further comparing these genes with the DEGs obtained from lengthways comparison (S2, S4 and S5 were selected), 138 genes were found (**Table S7**), containing: 1) 34 genes participated in the beginning of increase stage of glycitein content; 2) 46 genes participated in the termination of increase stage and the beginning of decrease stage; 3) 130 genes participated in the termination of decline stage; 4) 19 genes participated in the whole increase process; 5) 43 genes participated in the whole decline process; 6) 18 genes participated in the whole process of glycitein accumulation.

For the variation trend of genistin content is “two-part climbing mode”, the expression of genistin related major genes should be markedly different in S3-S4 (the first stage of increase of genistin content), S5-S6 (the second stage of the increase) and S6-S7 (the cessation of genistin accumulation). Based on this view, DEGs of 11 lengthways comparison groups were chosen for the investigation of candidate genes (**Table S7**). With gene functional annotation, 187 genes of genistin were attained. By comparing with the DEGs obtained from lengthways comparison (S3, S4, S5 and S7 were selected), 170 genes involved in the accumulation of genistin during the development of soybean were attained (**Table S7**). These candidate genes are consisted of: 1) 47 genes associated with the beginning of the first stage of genistin content increase; 2) 56 genes associated with the termination of the first stage; 3) 24 genes associated with the whole process of first stage; 4) 56 genes associated with the beginning of the second stage; 5) 90 genes associated with the termination of the second stage; 6) 63 genes associated with the whole process of the second stage; 7) 15 genes involved in the whole process of genistin accumulation.

Since the accumulation pattern of genistein content is “bipolar peak mode”, the differential expression of major genes related to genistein accumulation was found in S0-S1 (the rising part of the first peak of genistein content), S1-S2 (the falling part of the first peak), S5-S6 (the rising part of the second peak) and S8-S9 (the falling part of the second peak). The DEGs of 22 lengthways comparison groups were selected to investigate the genes modulating genistein accumulation (**Table S7**). A total of 212 genes were found. Further comparing with the DEGs of 4 lengthways comparison groups (S2, S4, S6 and S8), 83 candidate genes were selected (**Table S7**), containing: 1) 38 genes significantly affecting the beginning of the first stage of genistein accumulation; 2) 52 genes affecting the termination of the first stage; 3) 19 genes affecting the whole process of the first stage; 4) 45 genes affecting the initiation of the second stage; 5) 43 genes affecting the termination of the second stage; 6) 10 genes affecting the whole process of second stage; 7) only the gene, *Glyma.13G206700* (encoding the glucose-6-phosphate transporter), that affects the whole process of genistein accumulation.

Integrating all candidate genes, we found 60 genes that affect the accumulation of both daidzin and daidzein, 80 genes that affect the accumulation of both glycitin and glycitein, and 64 genes that affect the accumulation of genistein and genistein. Additionally, the results also showed that 25 genes regulated the accumulation of all components of glucoside isoflavones, while 39 genes modulated the accumulation of all components of aglycone isoflavones.

To sum up, 250 DEGs related to isoflavone accumulation were obtained in this study, including 76 genes influencing the accumulation of daidzin, 132 genes influencing the accumulation of daidzein, 119 genes influencing the accumulation of glycitin, 138 genes influencing the accumulation of glycitin, 170 genes influencing the accumulation of genistein, and 83 genes influencing the accumulation of genistein (**Table S7**).

#### 3.2.4 WGCNA

Based on the phenotypic and transcriptome data, the WGCNA was performed to analyze the internal relationship of isoflavone related genes. This study constructed 11 molecular modules (**Figures S5A, S5B**). Combining with the results of correlation analysis (**Figure S5C**), module significance analysis (MS) (**Figures S5D, S5E**) and iPath (**Figure S5F**), three modules (yellow, purple and turquoise) were suggested to play important roles in the biosynthesis of isoflavones. Additionally, although the brown module was judged as insignificant in correlation analysis, we still proposed that the brown gene module also plays an important role in isoflavones accumulation. Because: 1) the brown module consists of lots of candidate genes with high gene significance (GS) value; 2) the MS value of brown module is high; 3) according to the cluster analysis and correlation analysis (**Figure S5C**), the brown module is strongly correlated with yellow and turquoise module. Consequently, the four gene modules (**Table S8**), yellow, purple, turquoise and brown, should have vital functions in the accumulation of isoflavones in developing soybean seeds.

According to the GS analysis, 434 and 477 candidate genes were highly correlated with the glucosides and aglycone isoflavones respectively (GS ≥ 0.5), involving yellow, brown and purple molecular modules. Some candidate genes have been identified to participate in the accumulation of isoflavones, such as *Glyma.01G228700* (encoding chalcone synthase), *Glyma.07G202300* (encoding 2-hydroxyisoflavone synthase) and *Glyma.13G173401* (encoding Cytochrome P450 monooxygenases) (Dhaubhadel et al., 2003; Dhaubhadel et al., 2007; Guttikonda et al., 2010), which indirectly supported that the candidate genes detected based on WGCNA algorithm were highly credible.

In brief, the 4 gene clusters, yellow, purple, turquoise and brown (**Table S8**), were identified to have a great impact on the accumulation of isoflavones in the developing seeds of soybean. A total of 828 candidate genes with high GS value were also obtained, including 434 and 477 candidate genes correlated with glucosides and aglycones respectively.

#### 3.2.5 Time series analysis and comprehensive analysis

Thirty gene clusters were generated by time series analysis (**Figure S6**). The expression trend of genes of 24 clusters was close to that of genes promoting isoflavone accumulation, including cluster 1, cluster 2, cluster 3, cluster 6, cluster 9, cluster 10, cluster 11, cluster 12, cluster 13, cluster 14, cluster 15, cluster 16, cluster 17, cluster 18, cluster 19, cluster 20, cluster 21, cluster 22, cluster 23, cluster 25, cluster 26, cluster 27, cluster 29 and cluster 30. While the expression trend of genes of cluster 4, cluster 5, cluster 7, cluster 8, cluster 24 and cluster 28 was close to that of genes inhibiting isoflavone accumulation. Integrating the dynamic change of isoflavone content (**Figure 2**), the expression abundance variation of reported genes (**Figure S1**) and the results of gene function annotation, a total of 362 genes were given in this part, which may play an important role in the accumulation of isoflavones.

Finally, further Integrating with the results of DE analysis, WGCNA and time series analysis, 210 candidate genes were suggested to have a significant impact on the accumulation of isoflavones during soybean seed development.

### 3.3 Meta-analysis

#### 3.3.1 G1

The materials of G1 were divided into 5 categories: SN14, SN14a, SN14b, SN14c and SN14d. To analyze the whole genome expression profile of these samples, DE analysis (**Figure S7A**), WGCNA (**Figure S7B**) and times series analysis (**Figure S7C**) were performed. Initially, a total of 452 DEGs, functionally associated with the accumulation of isoflavone and its components, was obtained. Additionally, 241 hub genes, involved 12 molecular modules, were generated. With the functional annotation, 3 gene modules and 23 hub genes were mapped in this study (**Table S9**). Meanwhile, according to the results of time series analysis, the expression tendency of seven gene clusters (cluster 1, cluster 3, cluster 4, cluster 5, cluster 6 and cluster 7) was in accordance to the expression patterns of genes which promoted the isoflavone accumulation. The expression tendency of cluster 2 and cluster 9 seemed like the expression patterns of genes which suppressed the isoflavone accumulation.

#### 3.3.2 G2

The materials of G2 contained 4 developing stages of Wm82. To analyze the whole genome expression profile of these samples, DE analysis (**Figure S8A**), WGCNA (**Figure S8B**) and times series analysis (**Figure S8C**) were performed. Initially, a total of 362 DEGs, functionally associated with the accumulation of isoflavone and its components, were obtained. Additionally, 141 hub genes, involved 8 molecular modules, were generated. With the functional annotation, 3 gene modules and 16 hub genes were mapped in this study (**Table S9**). Meanwhile, according to the results of time series analysis, the expression tendency of four gene clusters (cluster 2, cluster 3, cluster 4 and cluster 7) was in accordance to the expression patterns of genes which promoted the isoflavone accumulation. The expression tendency of cluster 1, cluster 5, cluster 6, and cluster 9 seemed like the expression patterns of genes which suppressed the isoflavone accumulation.

#### 3.3.3 G3

The materials of G3 were divided into DN14 and DN14n. To analyze the whole genome expression profile of these samples, DE analysis (**Figure S9A**), WGCNA (**Figure S9B**) and times series analysis (**Figure S9C**) were performed. Initially, a total of 563 DEGs, functionally associated with the accumulation of isoflavone and its components, were obtained. Additionally, 161 hub genes, involved 7 molecular modules, were generated. With the functional annotation, 3 gene modules and 23 hub genes were mapped in this study (**Table S9**). Meanwhile, according to the results of time series analysis, the expression tendency of four gene clusters (cluster4, cluster 6, cluster 7 and cluster 8) was in accordance to the expression patterns of genes which promoted the isoflavone accumulation. The expression tendency of 4 clusters, including cluster 2, cluster 3, cluster 8 and cluster 9, seemed like the expression patterns of genes which suppressed the isoflavone accumulation.

#### 3.3.4 G4

The materials of G4 were divided into VCM and VCm. To analyze the whole genome expression profile of these samples, DE analysis (**Figure S10A**), WGCNA (**Figures S10B, C**) and times series analysis (**Figure S10D**) were performed. Initially, a total of 642 DEGs, functionally associated with the accumulation of isoflavone and its components, were obtained. Additionally, the RNA-seq data of VCM and VCm were used to perform WGCNA respectively, for there were significant difference between these two germplasms. A total of 121 hub genes, involved 6 molecular modules, were generated *via* analyzing the RNA-seq data of VCM; while 141 hub genes and 7 molecular modules were obtained *via* performing WGCNA with transcriptome profile of VCm. With the functional annotation, 3 gene modules and 13 hub genes were mapped in this study (**Table S9**). Meanwhile, according to the results of time series analysis, the expression tendency of seven gene clusters (cluster 2, cluster 3, cluster 5, cluster 6, cluster 7, cluster 8 and cluster 9) was in accordance to the expression patterns of genes which promoted the isoflavone accumulation. The expression tendency of cluster 1 and cluster 4 seemed like the expression patterns of genes which suppressed the isoflavone accumulation.

Briefly, a total of 1436 DEGs, 54 hub genes and 9 molecular modules (**Table S9**) were attained by summarizing the results of meta-analysis. With further combination of gene functional annotation and gene enrichment analysis, 292 genes which might affect the accumulation of isoflavones were acquired.

### 3.4 Stable major genes and molecular modules of isoflavone accumulation

The stable major genes and molecular modules of isoflavone accumulation were suggested in this study *via* the following steps: 1) integrating the results of transcriptome analysis and meta-analysis; 2) linking candidate genes with isoflavone-related QTL.

#### 3.4.1 Integrating the results of transcriptome analysis and meta-analysis

**Figure 4:**
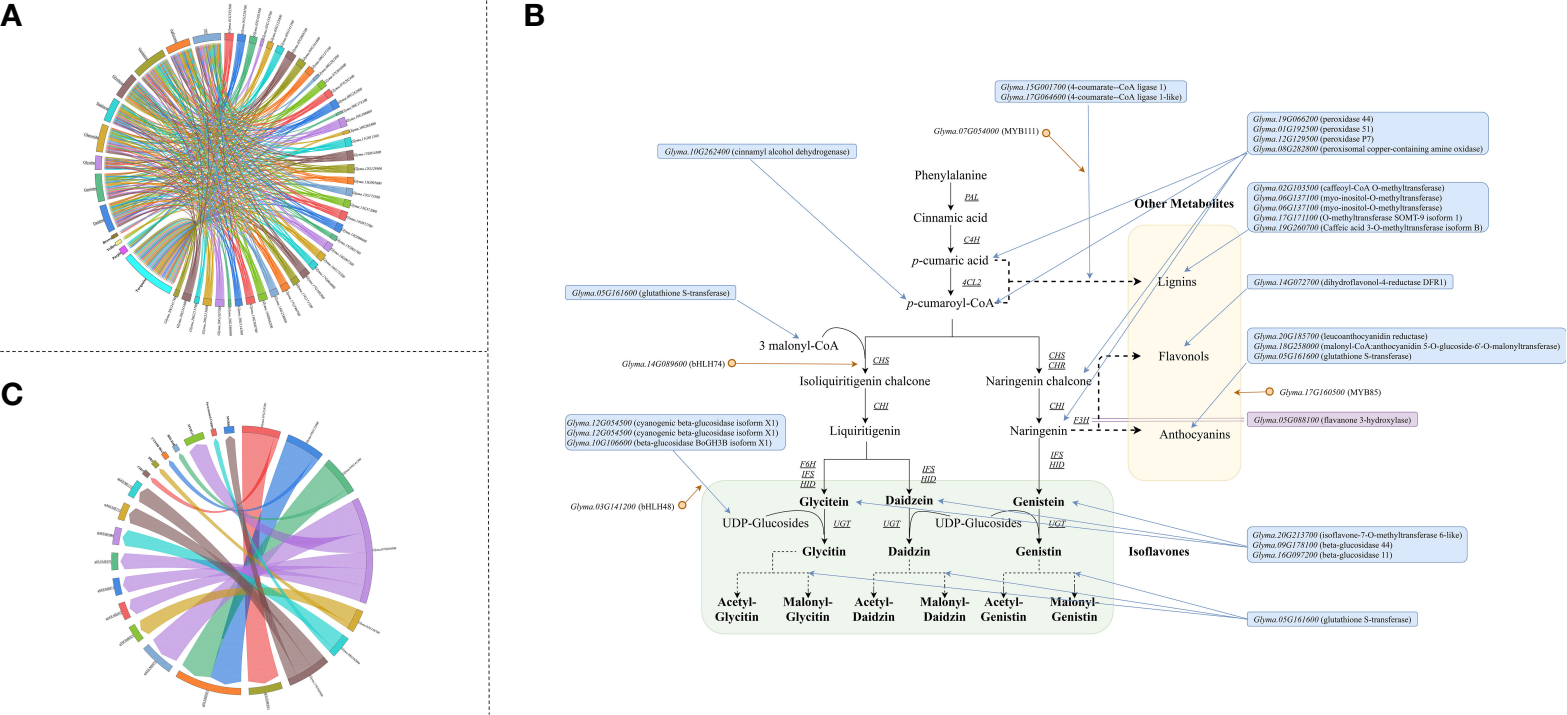
Major genes underlying isoflavone accumulation free from the limitation of environments **(A)** Major genes underlying isoflavone accumulation and their effects (The underlined words are isoflavone components, and the italicized words are QTL name, and the bold words are gene module generated by WCGNA); **(B)** QTG underlying isoflavone accumulation and corresponded stable QTL (The underlined words are isoflavone components, and the italicized words are gene name, and the bold words are gene annotation description); **(C)** Expansion of isoflavone biosynthesis pathway (The gene in the blue frame was predicted major gene, and the gene in the purple frame was the major gene identified by previous study, and the gene behind orange point was transcription factor. The arrow means the possible way that major genes regulate isoflavone).

Among the 210 and 292 candidate genes obtained from the transcriptome analysis and meta-analysis respectively, 41 genes were overlapped. Therefore, these genes are stable major genes which could affect the accumulation of isoflavones free from the limitation of environments and germplasms. (**Table S10** and **Figure 4A**).

Additionally, we obtained 4 gene modules and 9 meta-molecular modules *via* WGCNA with RNA-seq data and meta-data respectively. Comparing these modules, we found that each gene module could find at least 2 similar meta-molecular modules (some genes were overlapped). For instance, in the brown module: 1) 752 genes were similar to the blue module of G2 group meta-data; 2) 614 genes were similar to the blue module of G3 group meta-data; 3) 1195 genes were similar to the turquoise module of G4-VCM group meta-data; 4) 635 genes were similar to the blue module of G4-VCm group meta-data. This suggests that: 1) the internal effects and correlation of all molecular modules are rather stable; 2) the effects of these modules on isoflavone are quite stable. Hence, the purple, yellow, turquoise and brown module could regulate the isoflavone accumulation in developing soybean seeds free from the limitation of environments and germplasms.

Furthermore, *via* performing co-expression analysis and transcription factors analysis based on the major genes and molecular modules, four transcription factors were obtained: *Glyma.03G141200* (*bHLH48*), *Glyma.07G054000* (*MYB111*), *Glyma.14G089600* (*bHLH74*) and *Glyma.17G160500* (*MYB85*) (**Table S10** and **Figure 4A**). The following evidences in this study support that these transcription factors regulate the accumulation of isoflavones: 1) significantly differential expression were existed among high isoflavone lines and low isoflavone lines; 2) significantly differential expression were found during seeds development and accompanied by isoflavone variation; 3) GS analysis examined that these genes were highly correlated with the accumulation of various isoflavones; 4) according to the results of transcription factor target genes analysis, many major genes were regulated by them; 5) the strong correlation between transcription factor and target genes could be found in meta-analysis as well. Among them, *MYB111* has been demonstrated to regulate flavonoid synthesis in *Arabidopsis thaliana* by Stracke et al. (2007), while the other 3 genes were found for the first time.

#### 3.4.2 QTG analysis and isoflavones biosynthesis pathway expansion

Comparing the major genes with the 63 stable QTL (52 meta-QTL and 11 novel QTL) mapped in our previous study (**Table S2**) (Chen et al., 2021), 7 major genes were identified to be the QTG of 10 stable QTL (**Figure 4B**). Among them, 3 QTG encodes transcription factors, while 3 QTG encodes CYP450 protein. The QTL, qGLEME031, contains 2 QTG, *Glyma.03G141200* (*bHLH48*) and *Glyma.03G122000* (*CYP450 98A2*). *Glyma.07G054000* (*MYB111*) is the QTG of 3 stable QTL.

Additionally, on the basis of their effects and functions, these major genes could be divided into 5 categories: 1) 13 genes could directly take part in the biosynthesis of isoflavones; 2) 7 genes could regulate the biosynthesis of isoflavones directly by transcription regulation or post-transcription regulation; 3) 15 genes could indirectly influence isoflavone accumulation *via* regulating the biosynthesis of other related metabolites (such as anthocyanin); 4) 3 genes could mediately underly isoflavone synthesis through regulating the soybean seed growth and development; 5) the effects of 3 genes are unknown currently. Thus, *via* integrating the functions and internal interaction of major genes, the isoflavones biosynthesis pathway in soybean was supposed to be expanded (**Figure 4C**).

#### 3.4.3 The expression of major genes in soybean seeds during seed development

**Figure 5:**
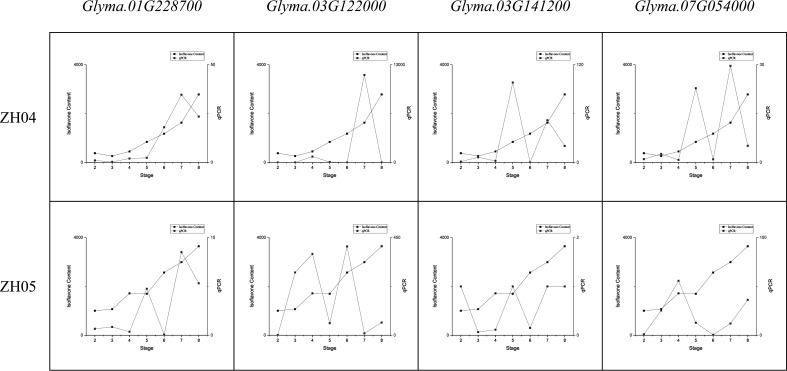
Comparing the variation of major genes expression and total isoflavone content during seed development.

A series of qRT-PCR assays showed that the major genes were constitutively expressed in the seeds of the ZH04 (low total isoflavone content) and ZH05 (high total isoflavone content) lines (**Figure 5**). Obviously, the expression trends of these major genes are consistent with the variation of isoflavone content. This result indicates that the expression of major genes in soybean seeds is related to isoflavone content, which also suggested that our results are reasonable and reliable.

#### 3.4.4 Dynamic molecular mechanisms regulating isoflavone accumulation in developing seeds

Combining with the major genes and accumulation pattern, the process of isoflavone accumulation can be divided into three phases: phase I (S1-S3, the initiation of synthesis), phase II (S4-S6, the main synthesis and rapid variation) and phase III (S7-S9, the termination of synthesis).

**Figure 6:**
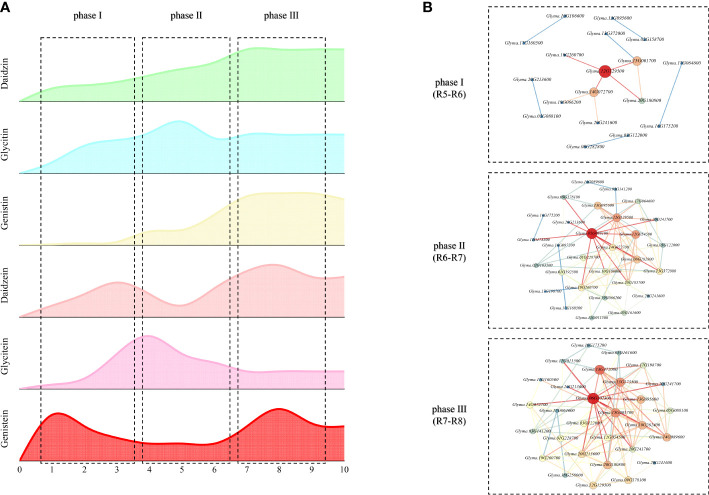
Dynamic molecular mechanisms regulating isoflavone accumulation in developing seeds **(A)** Trend of isoflavone dynamic variation; **(B)** Dynamic molecular network regulating isoflavone.

With further combination of the association analysis, the dynamic molecular patterns regulating isoflavones accumulation in developing soybean seed are characterized in **Figure 6**. There are three obvious rules: 1) the genes that have the most impact vary from time to time; 2) a single gene might show antipodal effects in different phase; 3) some genes modulate isoflavones accumulation in one phase, while their homologous genes might regulate the accumulation in another phase. The internal reasons of these rules are suggested as follows: 1) the variation of key genes might be depended on the plant growth factors, like phytohormone, for the main life activity of plants is determined by their growth stage whose variation are controlled by plant growth factors; 2) the antipodal effects of same genes might be developed by the negative control mechanisms of plants; 3) the temporal variation of homologous genes may be regulated by photoperiod signal transduction pathway.

In a word, this research: 1) characterized the dynamic variation of isoflavone content; 2) obtained 41 stable major genes and 4 molecular modules influencing isoflavone accumulation; 3) expanded the isoflavone biosynthesis pathway; 4) explored the dynamic molecular patterns regulating isoflavones accumulation in the developing seed of soybean.

## 4 Discussion

### 4.1 Dynamic variation of six isoflavone components

During soybean seed development, there are huge and significant differences in the dynamic variation of various isoflavones.

The accumulation pattern of daidzin is “climbing mode”. Three noticeable characteristics of its variation are suggested: 1) the first gap between high daidzin contents lines and low daidzin contents lines generally occurred in S4 period; 2) the higher daidzin contents lines could maintain their biosynthesis rates, while the rate of lower lines decreased at S4; 3) the accumulation rate of high daidzin contents lines was almost equivalent to that of the low daidzin contents lines, but the accumulation duration in higher lines was obviously longer than lower lines. Thus, the uppermost reason determining the daidzin contents in matured seeds of soybean should be the accumulation duration, rather the accumulation rates. With further speculation, the current research suggests: 1) the daidzin contents could be regulated by modulating seed growth period; 2) the expression of daidzin related genes in different lines is similar in early developing stage, but differs in late developing stage.

The accumulation pattern of daidzein is “two peaks mode”. According to the variation of daidzein content in different lines, there is no significant correlation between the peak value and final content. For example, the peak value of HF25 (the parent with lower daidzein content) was noticeably greater than the peak value of ZD27 (the parent with higher daidzein content). Additionally, the germplasms with high daidzein contents generally reached their peak later than the low-daidzein content germplasms did. Consequently, summarizing these phenomena, this study demonstrates that: 1) the daidzein contents in matured seed might be determined by not only the biosynthesis of daidzein, but also the decomposition and metabolism, and latter might be more important; 2) most of daidzein synthesized in the early stage of seed development would be further metabolized, while the daidzein accumulated in late stage of seed development would be stored; 3) daidzein contents are modulated by growth-regulating factors (GRF), as same as daidzin contents are.

The accumulation pattern of glycitin is “single peak and climbing mode”. An obviously characteristic is that the glycitin contents reach equilibrium at S6, siginificantly earlier than other isoflavone components. This indicates that the accumulation of glycitin is a short-time activity and centered in the early development stage. In addition, the glycitin contents of parents were similar, but a significant phenotype variation is found in their RIL lines. This suggests that: 1) although the parents are similar at phenotype level, their genotypes may be diverse; 2) the variation within group is quite large, and a lot of lines with different genotypes are generated; 3) many glycitin related genes may be mutated.

The accumulation pattern of glycitein is “single peak mode”. A strong correlation is existed between seed glycitein contents and length of soybean growth period, which could be inferred from two special samples: ZD27 and ZH04. Generally, glycitein contents increased during S1-S4, and decreased slowly during S4-S7, and then kept constant till the end. However, the variation of glyceitin contents in ZD27 started at S2 and ended at S5, which exhibits a short and early variation process; while ZH04 had a long and late accumulation process which reached the peak at S7 and ended at S8. The glyceitin contents of ZD27 is lower than average, while the glyceitin content of ZH04 is the highest one in this research. We infer that the distinction of growth duration period leads to the diverse accumulation processes of glycitein in different germplasms. Indeed, two rules might be suggested: 1) the glycitein contents are depended on the ratio of synthesis rate and resolving rate; 2) in accordance with the accumulation patterns of daidzein, the glycitein synthesized in early developing stage tends to be further metabolized, while the glycitein accumulated in late developing stage is apt to storage. Since the glycitein contents rapidly decreased during S4-S7, but other isoflavones did not variate in this way, a specific regulation mechanism might regulate this process and does not influence the accumulation process of other isoflavones.

The accumulation pattern of genistin is “two-part climbing mode”. According to the variation tendency, the gap of genistin contents in different lines starts to increase at S4, and the genistin contents in matured seed is determined by its total synthesis in the rapid accumulation phase (S5-S7) in a large part. In this period, the lines with higher genistin content produced more genistin isoflavone, while the lower genistin lines would accumulate less genistin content. Additionally, the accumulation of genistin is relatively later than other components, which is a typical metabolite synthesized in the late developing stage. The synthesis rate of this kind of metabolites would be significantly influenced by their precursors’ contents accumulated in early developing stage. With the combination of the dynamic variation of genistein this study supports that the biosynthesis of genistin depends on the genistein content accumulated during S1-S4. With further speculation, the expression of genistin related genes would be higher after S3, but lower before S3.

The accumulation pattern of genistin is “two-part climbing mode”. As its variation in developing seed shown, three rules could be summed up: 1) lines whose first peak is high usually contain lower genistin contents finally, while lines whose first peak is low always contains a higher genistein contents in the end; 2) the first peak of each line almost occurs at S1, but their second peak distributes at various stages; 3) the second peak value and final contents would be higher if the second peak occurs later.

Although there are significant differences in the accumulation patterns of various isoflavones, several common rules appropriate to all components are found. First of all, the biosynthesis of all isoflavones is mainly took place during S2-S8, and the synthesis of aglycones is lightly earlier than of glucosides generally. Secondly, the first gap of contents between high isoflavone line and low isoflavone line appears during S2-S4. Additionally, most of isoflavone synthesized in early development stage would be further metabolized and/or decomposed, while isoflavone synthesized in late development stage would be stored. Finally, one of the most important factors determining isoflavone contents in matured seed is the duration of synthesis during the soybean development. Generally, high isoflavone cultivars always have long synthesis duration.

### 4.2 Major genes and molecular modules related to isoflavone contents

#### 4.2.1 Major genes and their effects

By integrating transcriptome analysis and meta-analysis, a total of 41 major genes influencing the accumulation of isoflavones were suggested, including 28 novel major genes.

Four transcription factor genes, *MYB111* (*Glyma.07G054000*), *bHLH48* (*Glyma.03G141200*), *bHLH74* (*Glyma.14G089600*) and *MYB85* (*Glyma.17G160500*), were attained. *MYB111* has been demonstrated to regulate flavonoid synthesis in *Arabidopsis thaliana* (Stracke et al., 2007), but its downstream genes and related mechanisms were still unknown. Based on the TF analysis and co-expression analysis, this study argues that its impacts on isoflavone accumulation might be *via* regulating *4CL1*. The other three TF genes were detected for the first time, and related mechanisms are predicted as: 1) the effects of *bHLH48* might be mediated by *Glyma.10G106600* (beta-glucosidase BoGH3B isoform X1) regulating the contents of UDP-glucosides; 2) *bHLH74* should regulate *CHS7*; 3) *MYB85* should indirectly affect isoflavone accumulation *via* regulating anthocyanidin pathway mediated by *Glyma.18G258000* (encoding malonyl-CoA: anthocyanidin 5-O-glucoside-6’-O-malonyltransferase).

One interesting finding is that lots of peroxidase-related genes are suggested to be major genes in the current study, such as peroxidase 51 (*Glyma.01G192500*). The dynamic variation and expression patterns of these major genes are conformed to the definition of major genes influencing isoflavone synthesis. Thus, we identified the peroxidase-related genes affecting isoflavone accumulation in plants. This study also found that: 1) the up-regulated expression of these genes is always accompanied by the decrease of the contents of isoflavone; 2) based on the GS analysis, the gene is strongly negatively correlated with the accumulation of all isoflavones; 3) these genes are expressed together with *Glyma.19G260700* (Caffeic acid 3-O-methyltransferase isoform B), *Glyma.20G185700* (leucoanthocyanidin reductase) and *Glyma.14G072700* (dihydroflavonol-4-reductase DFR1). Thus, three points of views are put out: 1) the effects of peroxidase-related genes in isoflavone accumulation were negative; 2) these genes should influence isoflavone synthesis *via* regulating the pathway of *p*-cumaric acid synthesizing to Naringenin (including *p*-cumaric acid, *p*-cumaroyl-CoA, Chalcone, Naringenin-chalcone and Naringenin); 3) the negative effects of these genes should be resulted by promoting the intermediate metabolites of isoflavone pathway (like *p*-cumaric acid, *p*-cumaroyl-CoA, Chalcone, Naringenin-chalcone and Naringenin) synthesizing to other secondary metabolites, such as lignin, flavanol and anthocyanidin.

Another interesting finding is centered by *IFS1* (*Glyma.07G202300*) and *IFS2* (*Glyma.13G173500*). *IFS* is a quite important gene for isoflavone synthesis, for it 1) encodes protein catalyzing the first committed step of isoflavone biosynthesis; 2) determines whether plants produce isoflavone (Jung et al., 2000). Hence, the intuitive view, up-regulated expression of *IFS* should lead to higher production of isoflavone in soybean seed, and could be easy to accepted. In this study, however, neither *IFS1* nor *IFS2* are not suggested to have great impacts in improving the production of isoflavones. We found that they are notably highly expressed in ZD27 during S5-S7 comparing with ZH01, ZH02 and HF25, while the isoflavone content of ZD27 is higher than ZH02 and HF25, but equal to ZH01. Additionally, the highly expressive stages of *IFS* were later than the stages of isoflavones rapid accumulation, and weak correlation between isoflavones and *IFS* are found. With the combination of the above views and phenomenon, two assumptions are proposed: 1) *IFS* does determine isoflavone synthesis in plants, but does not regulate isoflavone contents; 2) another *IFS* homologous gene are existed, whose high expression could offset low expression levels of *IFS1* and *IFS2*. We tend more to support the first hypothesis, because *IFS* homologous gene is not found in this study. Though several major genes detected in this study are function-unknown gene, the results of sequence alignment between *IFS* and them do not support they are homologous. With further deduction on the basis of the first assumption, isoflavone synthase may have a long median life expectancy and could catalyze the biosynthesis of isoflavone rapidly, and therefore the pathway does not require too many IFS proteins. Consequently, the activity and number of IFS would not restrict the rate of isoflavone synthesis in developing soybean seeds generally, and up-regulated expression of *IFS* would not lead to higher production of isoflavone.

#### 4.2.2 Potentially new regulation mechanisms of isoflavone synthesis

The isoflavone synthesis is regulated by multiple factors, here we propose two potential regulation mechanisms below.

The rules of isoflavones dynamic change in this study suggests that a strong correlation should be existed between the seed development and isoflavone accumulation. Several evidences in this study imply that the essence of this relationship is photoperiod regulating isoflavone accumulation, and related mechanism might be mediated by 3 major genes, *CHS4a* (*Glyma.08G110500*), *CHS7* (*Glyma.01G228700*) and *bHLH74* (*Glyma.14G089600*). First of all, compared with the early-matured cultivars and lines (like HF25, SN14 and ZH02), the expression abundance of *CHS* is obviously high in late matured samples (like ZD27 and Wm82). In addition, *CHS* is involved in isoflavone synthesis and circadian clock, according to the gene functional annotation. The expression correlation analysis also suggests that several circadian clock genes are significantly correlatively expressed with *CHS7*, such as *Glyma.07G048500* (*Late-elongated hypocotyl*, *LHY*) and *Glyma.14G049700* (encoding E3 ubiquitin-protein ligase COP1). Additionally, this study also found that *CHS7* and these circadian clock genes were regulated by *bHLH74*. Actually, the view, *CHS* involved in photoperiod, has been identified in *Arabidopsis*. Jenkins et al. (2001), based on the experiments with photoreceptor mutants, discovered that distinct UV-A/blue (cry mediated) and UV-B photoreception systems control *CHS* expression. Hildreth et al. (2022) found that the transcriptional activity of two key circadian clock genes, *CCA1* (*Circadian clock associated 1*, also known as *LHY*) and *TOC1* (*Timing of cab expression 1*), was altered in *CHS*-deficient seedlings across the day/night cycle. Several research indirectly supported that *bHLH74*, a circadian clock related TF, influences isoflavone accumulation were also found. Li et al. (2019) demonstrated that the down-regulation of *bHLH74* would inhibit anthocyanin (a secondary metabolite, which has a common precursor, chalcone, with isoflavones) accumulation in *Arabidopsis*. *Via* putting the above points and evidences together, the possible molecular mechanism of photoperiod regulating isoflavone accumulation is inferred: photoreception systems, *via* activating the downstream signal transduction pathway, up-regulate *bHLH74* to promote the expression *CHS*, and therefore accelerate isoflavone accumulation.

Another possible new mechanism modulating isoflavone synthesis was mediated by *4CL1* and *4CL2*. According to their tissues-gene expression atlas (**Figure S11**), drawn on the basis of Soybean Expression Atlas database (Machado et al., 2020), we found:1) *Gm4CL1* (*Glyma.15G001700*) and *Gm4CL2* (*Glyma.13G372000*) are tissue-specific genes; 2) the expression of *Gm4CL1* and *Gm4CL2* could be also approximative in some tissues, such as flower, pod and seed. In addition, according to Lindermayr et al. (2002) and Schneider et al. (2003), *Gm4CL1* takes part in the biosynthesis of lignin, while *Gm4CL2* takes part in the biosynthesis of isoflavone in soybean. Hence, three opinions are proposed: 1) the functions of *Gm4CL1* and *Gm4CL2* are different; 2) their expression and effects might not be mutual interference; 3) the impacts of *Gm4CL1* on isoflavones are weak. In this study, however, *Gm4CL1* is not only negatively correlated with isoflavone accumulation in soybean, but also significantly up-regulated expressed in HF25 (low isoflavone contents germplasm) comparing with ZD27 and ZH01 (high isoflavone contents germplasms). While *Gm4CL2* is neither strongly correlated with isoflavones accumulation, nor significantly differently expressed among high and low isoflavone contents germplasms. Moreover, the expression of *Gm4CL2* is similar in ZD27 and HF25, while of *Gm4CL1* is obviously up-regulated in HF25 (low isoflavone content cultivars) at S5 and S7 (two important stages of isoflavone synthesis) comparing with ZD27 (high isoflavone content cultivars). Thus, this study proposes that *Gm4CL1* could strongly and negatively regulate isoflavone accumulation in soybean, and the negative effects of *Gm4CL1* might be generated by 4CL1 protein competing substrates with 4CL2. To be more specific, 4CL1 and 4CL2 synthesize different products *via* bonding same substrates (*p*-cumaric acid and *p*-cumaric-CoA), and more substrates would be utilized by 4CL1 when the expression of *Gm4CL1* is increased, and therefore less precursors of isoflavones would be generated by 4CL2 with decreased isoflavones.

### 4.3 Summary and further research avenue

Based on the developing seeds of soybean from 2 parents and 6 RILs, and combined with the reported QTL and meta-analysis method, this study: 1) characterized the dynamic changes of isoflavone contents; 2) revealed the accumulation patterns of six isoflavone compounds at both physiological and molecular level; 3) obtained 28 novel major genes and 4 molecular modules associated with isoflavones accumulation; 4) expended the isoflavone biosynthesis pathway. The results will give a meaningful guidance for the molecular design and breeding of elite soybean cultivars with high isoflavone contents. Meanwhile, investigating the functional differences between *Glyma.15G001700* (*4CL1*) and *Glyma.13G372000* (*4CL2*), and the molecular mechanisms of growth-regulating factors (GRF) modulating the isoflavone contents are required for future research.

## Data availability statement

The data presented in the study are deposited in the NCBI repository, accession number PRJNA895728.

## Author contributions

HC and QYZ designed the research; HC, CKL, XW and YSL conducted the experiments and analyzed the data; FFW and XWP conducted the field trial; HC and QYZ wrote and revised the manuscript. These authors' contribution is equally: HC and CKL. All authors read and approved the manuscript.

## Supplementary material

Supplementary Figure 1 Dynamic variation of reported genes associated with isoflavone accumulation.

Supplementary Figure 2 The quality control, evaluation and quantification of RNA-seq data **(A)** The histogram of data filtering; **(B)** The histogram of data comparative analysis; **(C)**The histogram of mapped reads distribution; **(D)** The graph of gene covering analysis; **(E)** The box plot of gene expression distribution.

Supplementary Figure 3 Verification of RNA-Seq results by qRT-PCR.

Supplementary Figure 4 Distribution heap diagram of DEGs.

Supplementary Figure 5 Results of WGCNA in this study **(A)** Gene dendrogram and module colors; **(B)** Correlation between modules; **(C)** Correlation between module and trait; **(D)** Correlation between gene and trait; **(E)** Module significance (GS) between module and trait; **(F)** Metabolite pathways of molecular module.

Supplementary Figure 6 Results of time series analysis.

Supplementary Figure 7 Transcriptome analysis of G1 meta-data.

Supplementary Figure 8 Transcriptome analysis of G2 meta-data.

Supplementary Figure 9 Transcriptome analysis of G3 meta-data.

Supplementary Figure 10 Transcriptome analysis of G4 meta-data.

Supplementary Figure 11 Expression level of *Gm4CL1* and *Gm4CL2* in various tissues.

Supplementary Figure 12 GO annotation and enrichment analysis of Yellow molecular module.

Supplementary Figure 13 GO annotation and enrichment analysis of Purple molecular module.

Supplementary Figure 14 GO annotation and enrichment analysis of Brown molecular module.

Supplementary Figure 15 GO annotation and enrichment analysis of Turquoise molecular module.
