# Supplementary material for: Developmental dynamic transcriptome and systematic analysis reveal the major genes underlying isoflavone accumulation in soybean
Source: Front Plant Sci. 2023 Mar 7;14:1014349. doi: 10.3389/fpls.2023.1014349 (PMC10027745; doi:10.3389/fpls.2023.1014349)
Supplement: Supplementary Figure 1 — Dynamic variation of reported genes associated with isoflavone accumulation. [file DataSheet_1.pdf]

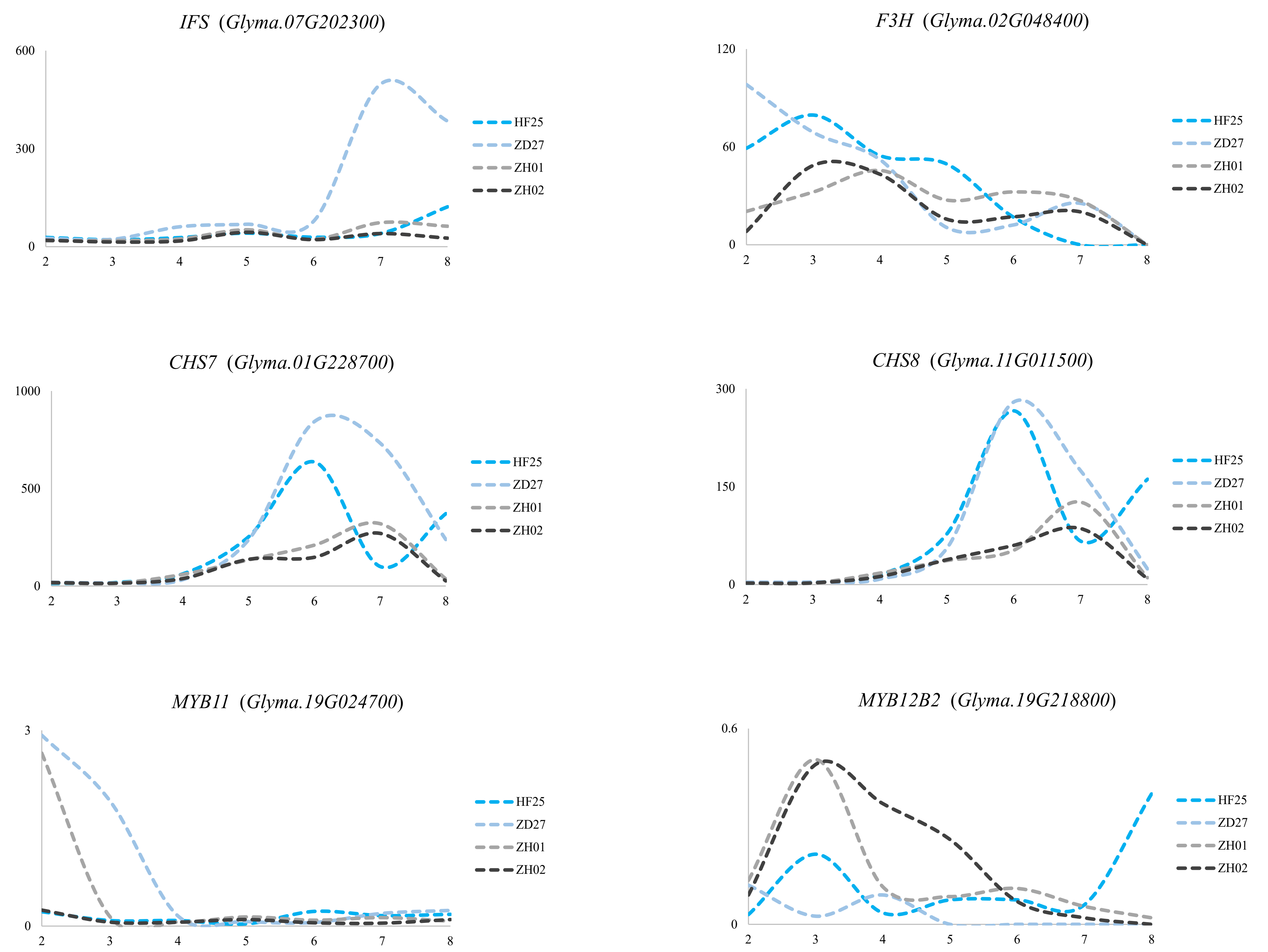

The selected genes influencing the accumulation of isoflavone contents

Gene Name	Gene ID [a]	Description	Effect [b]	Reference
F3H	*Glyma.02G048400*	Gene of anthocyanin pathway	-	Nagamatsu et al., 2009
IFS	*Glyma.07G202300*	Isoflavone synthase	+	Jung et al., 2000
CHS7	*Glyma.01G228700*	Chalcone synthase	+	Dhaubhadel et al., 2007
CHS8	*Glyma.11G011500*	Chalcone synthase	+	Dhaubhadel et al., 2007
MYB11	*Glyma.19G024700*	Transcription factor	+/-	Pandey et al., 2015
MYB12B2	*Glyma.19G218800*	Transcription factor	+/-	Li et al., 2013

[a] **Gene ID** was based on Wm82.a4.v1 assemblies

[b] **Effect:** The symbol, "+", refers to promote; the symbol, "-", refers to restrain; the symbol, "+/-", refers to regulate
