# Supplementary figures and images for: Developmental dynamic transcriptome and systematic analysis reveal the major genes underlying isoflavone accumulation in soybean
Source: Front Plant Sci. 2023 Mar 7;14:1014349. doi: 10.3389/fpls.2023.1014349 (PMC10027745; doi:10.3389/fpls.2023.1014349)

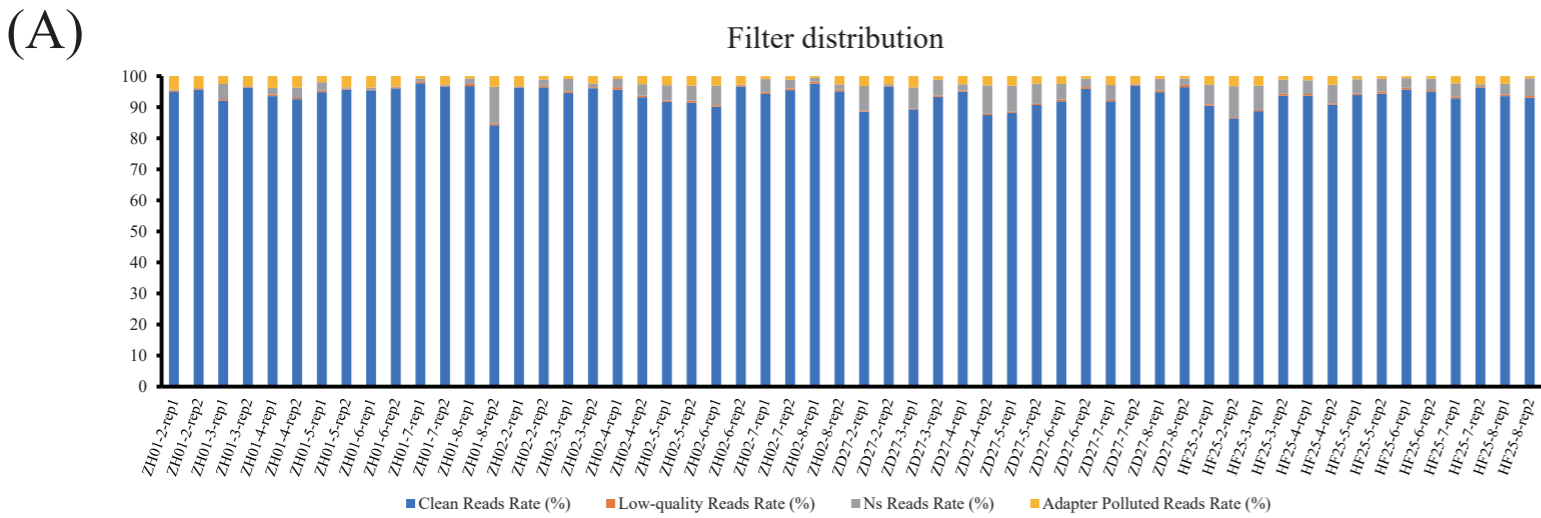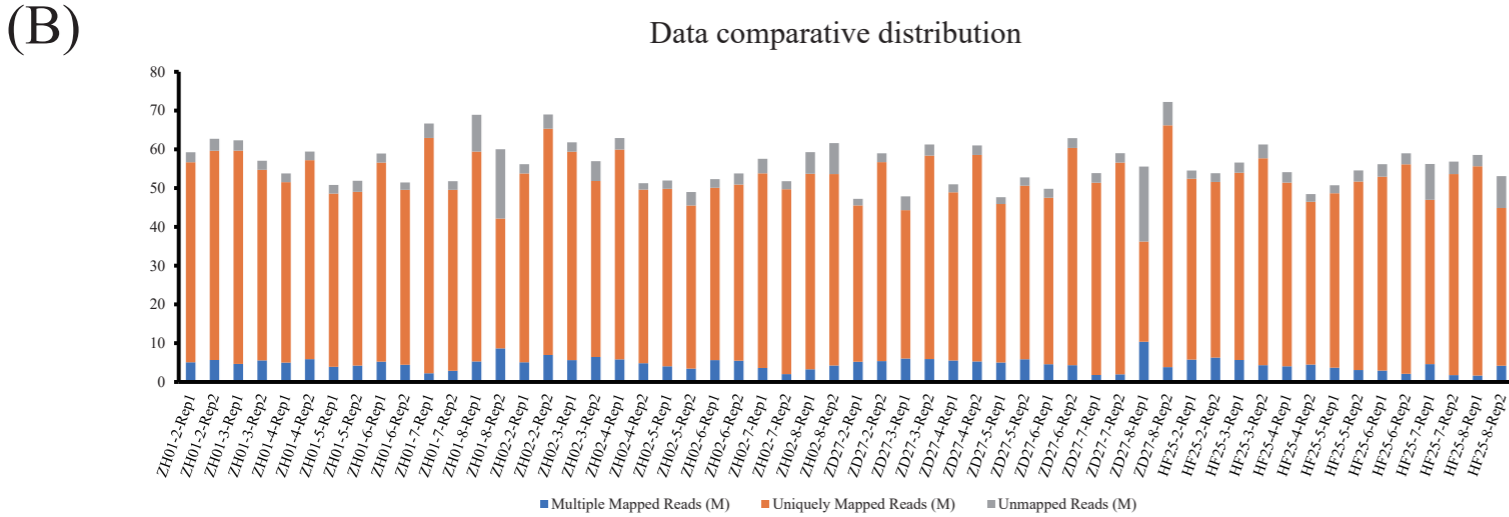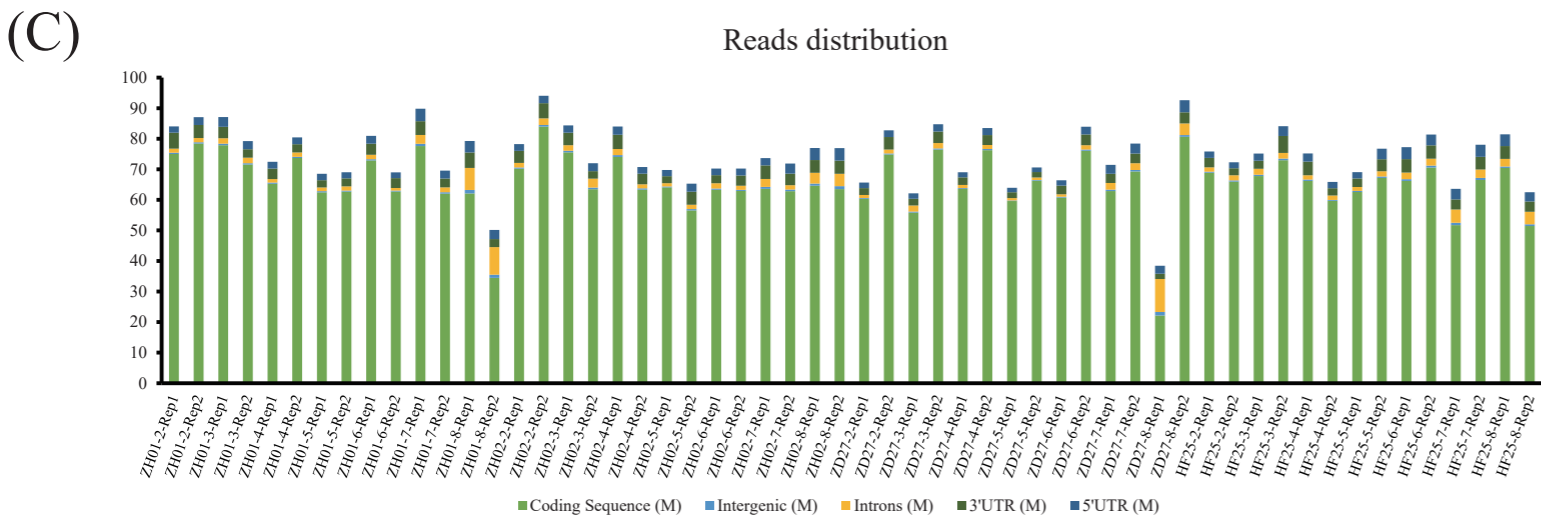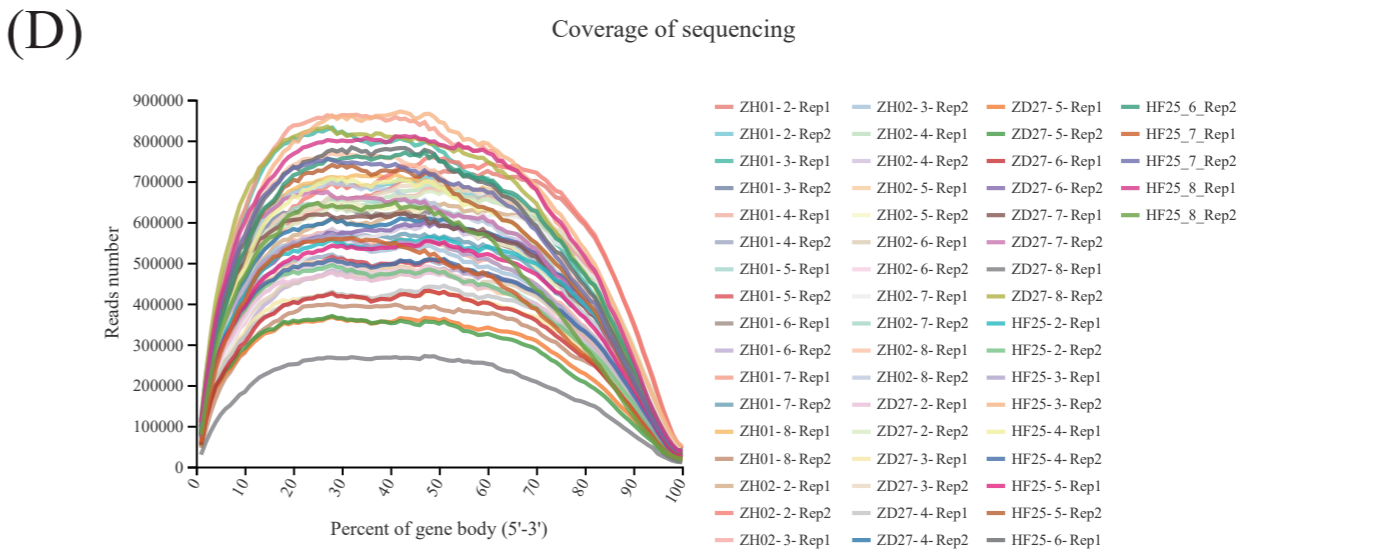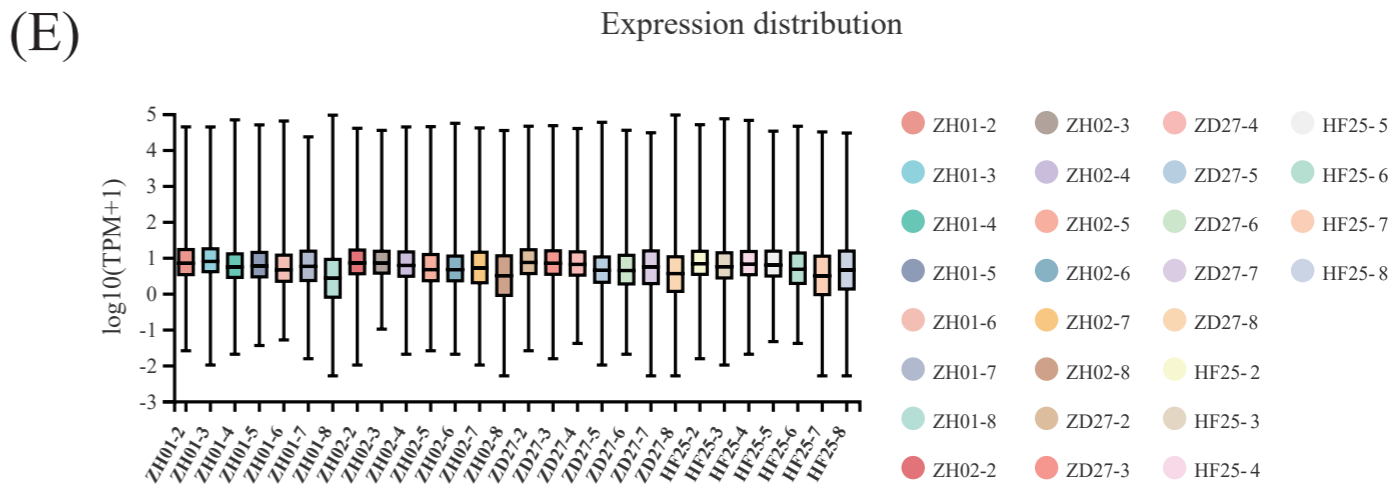

Supplement: Supplementary Figure 2 — The quality control, evaluation and quantification of RNA-seq data (A) The histogram of data filtering; (B) The histogram of data comparative analysis; (C)The histogram of mapped reads distribution; (D) The graph of gene covering analysis; (E) The box plot of gene expression distribution. [file DataSheet_2.pdf]

*Glyma.06G290100*

*Glyma.06G303100*

*Glyma.07G268100*

*Glyma.12G103100*

*Glyma.13G329000*

*Glyma.17G053300*

ZD27

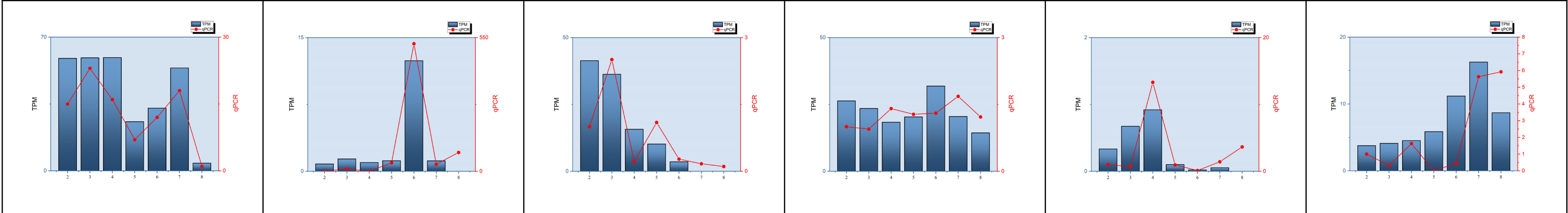

HF25

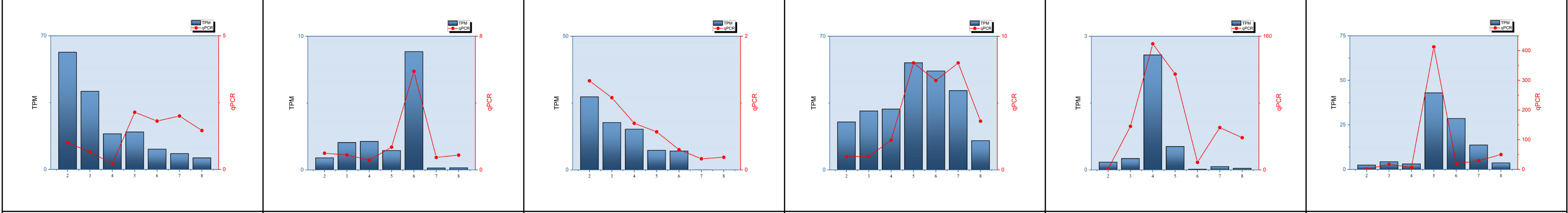

ZH01

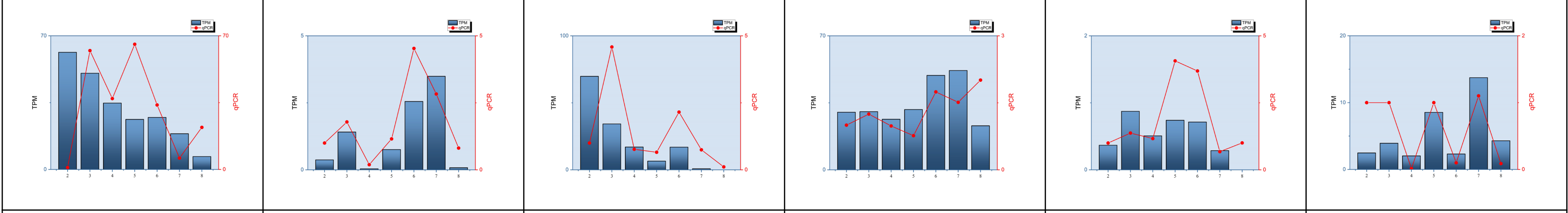

ZH02

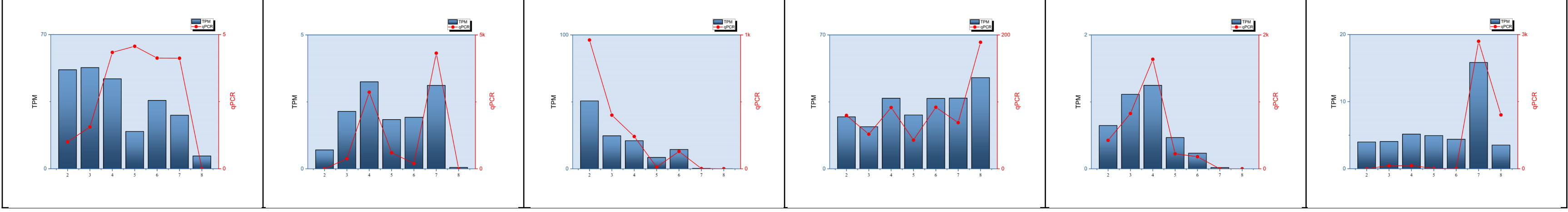

Supplement: Supplementary Figure 3 — Verification of RNA-Seq results by qRT-PCR. [file DataSheet_3.pdf]

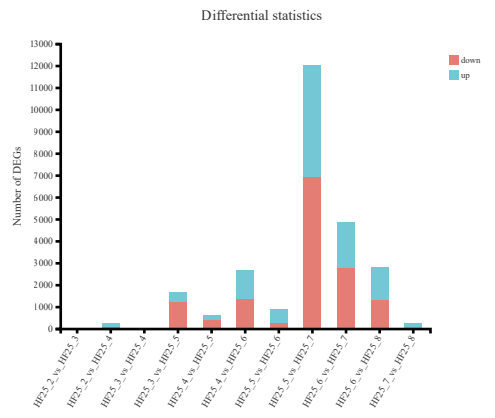

HF25

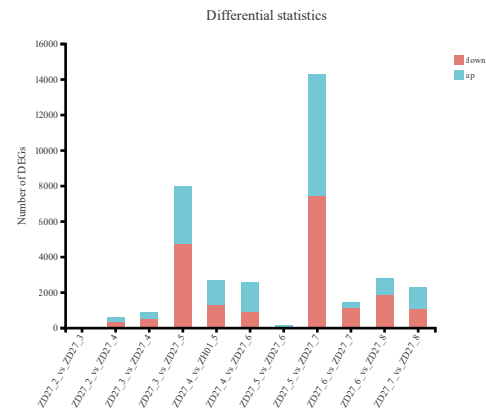

ZD27

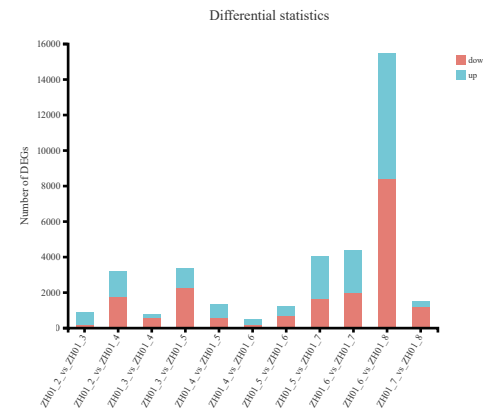

ZH01

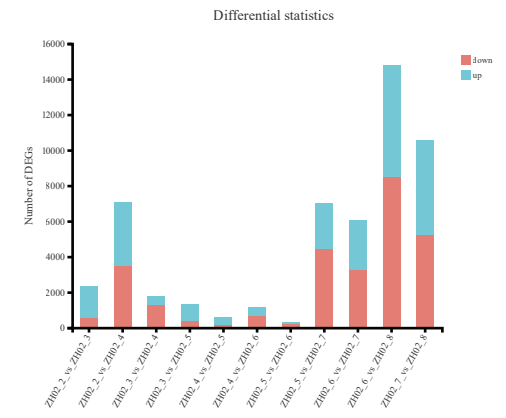

ZH02

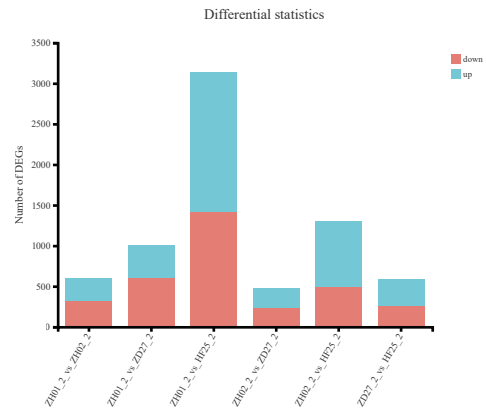

S2

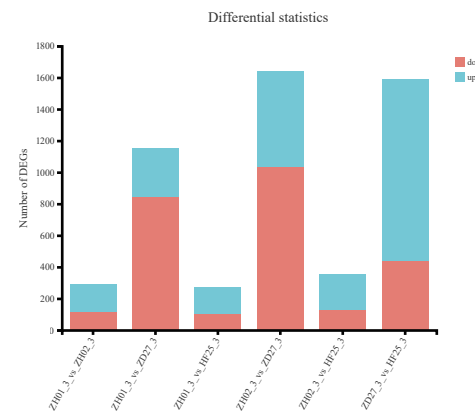

S3

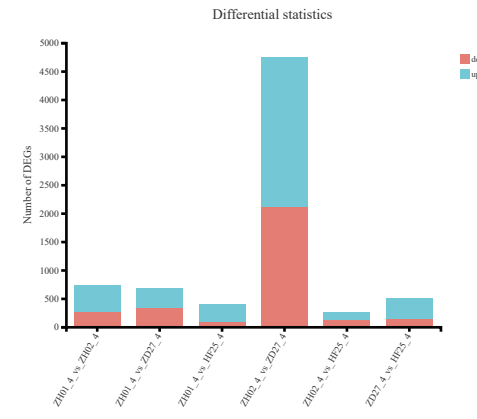

S4

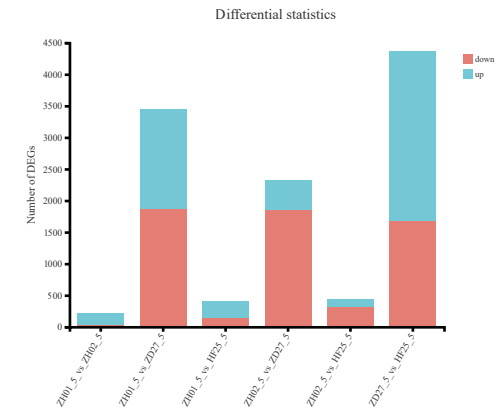

S5

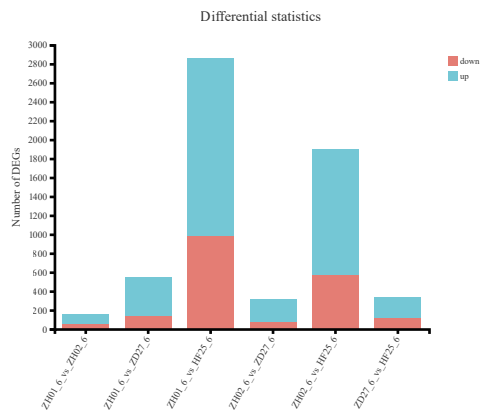

S6

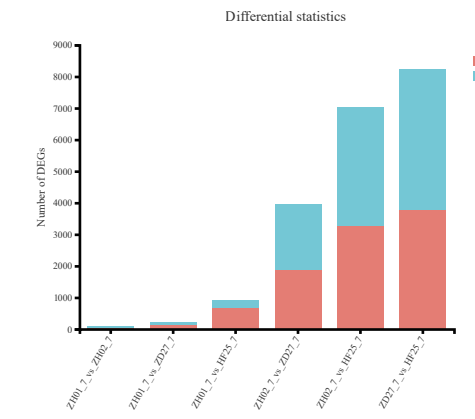

S7

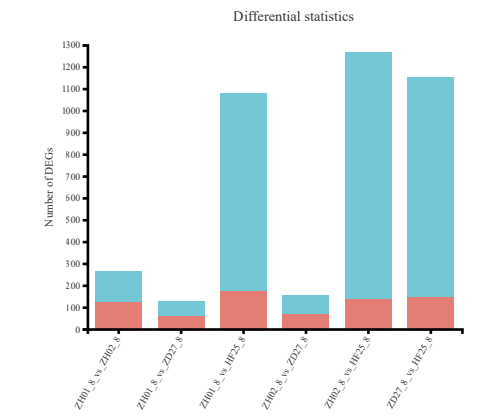

S8

Supplement: Supplementary Figure 4 — Distribution heap diagram of DEGs. [file DataSheet_4.pdf]

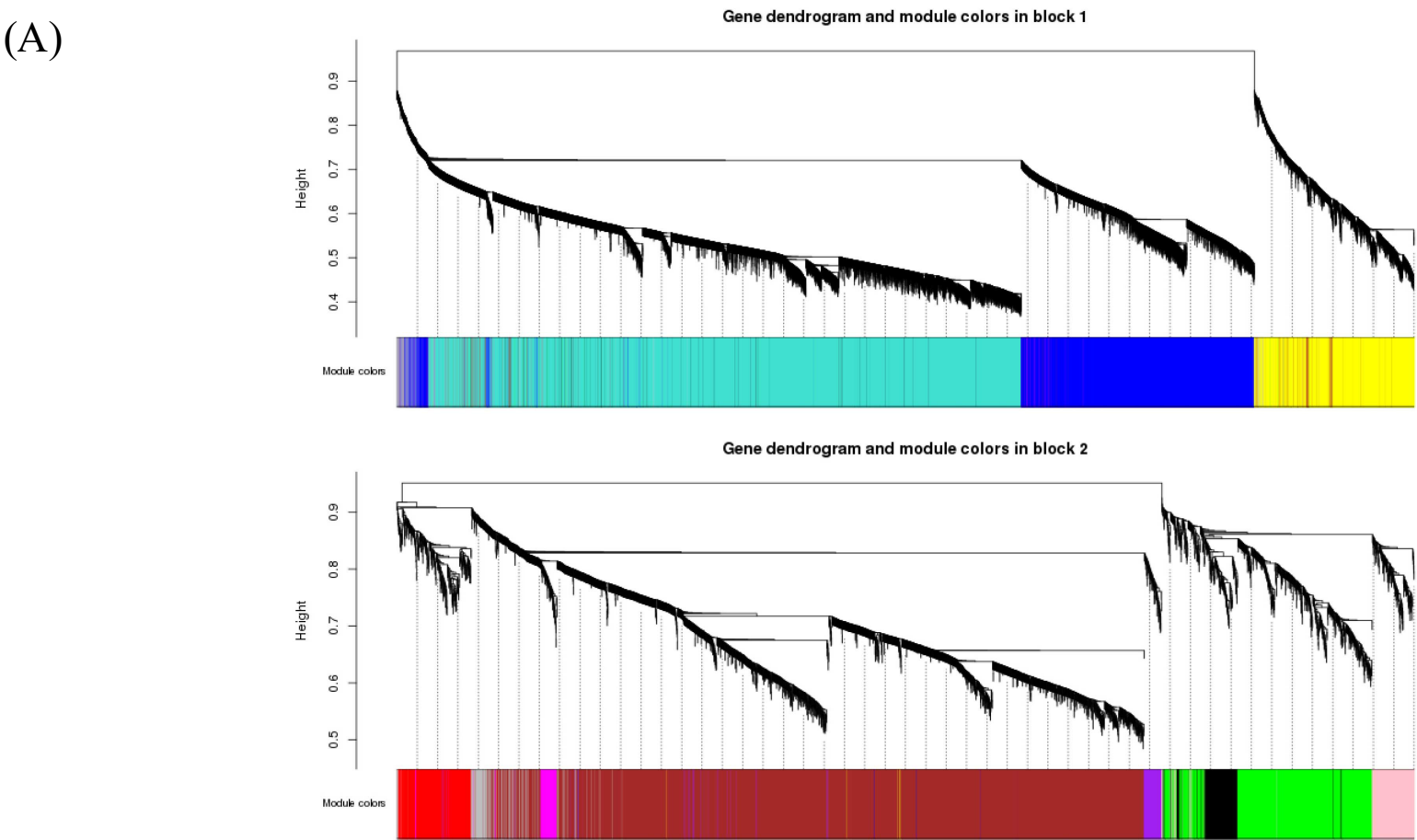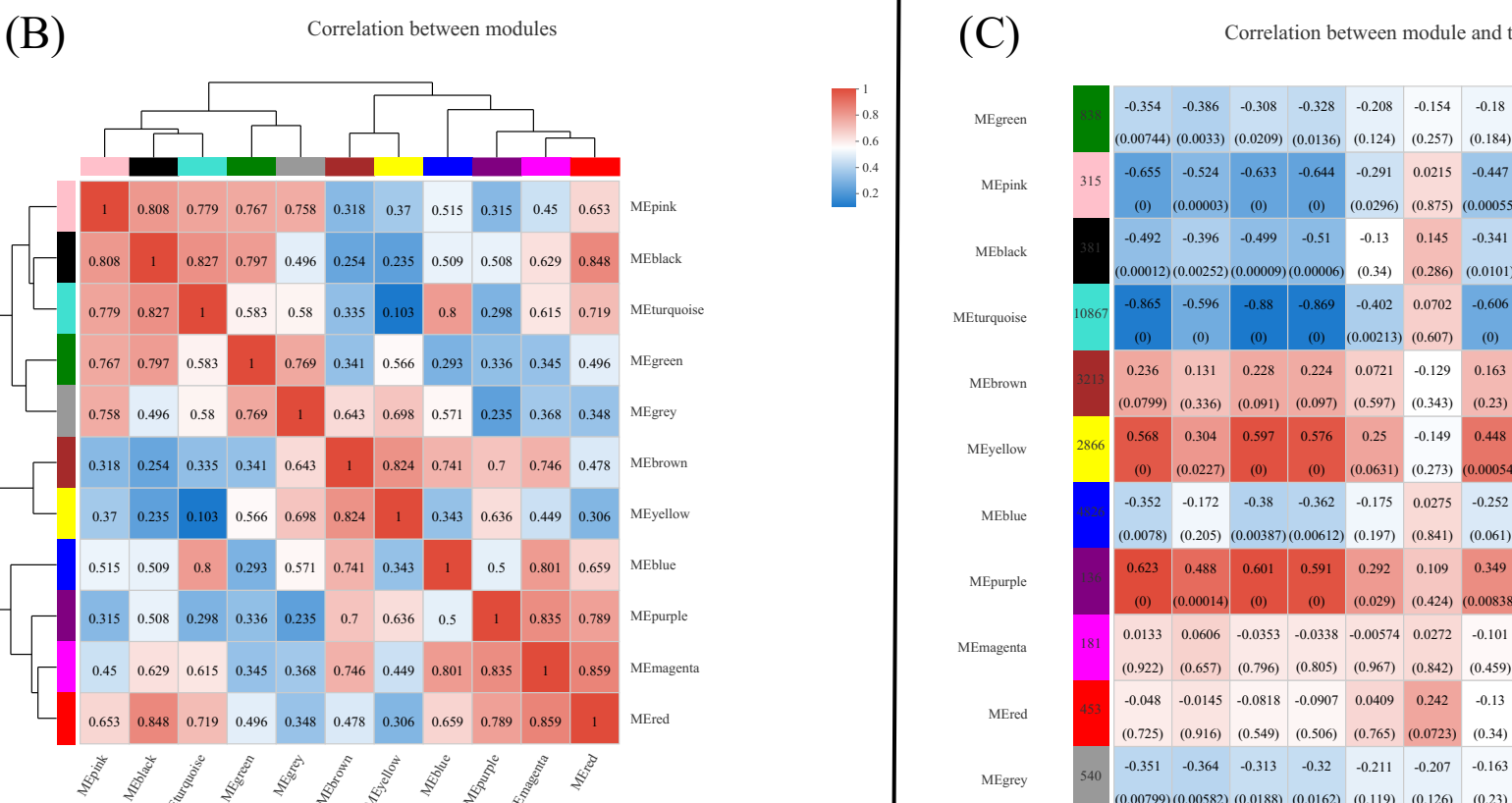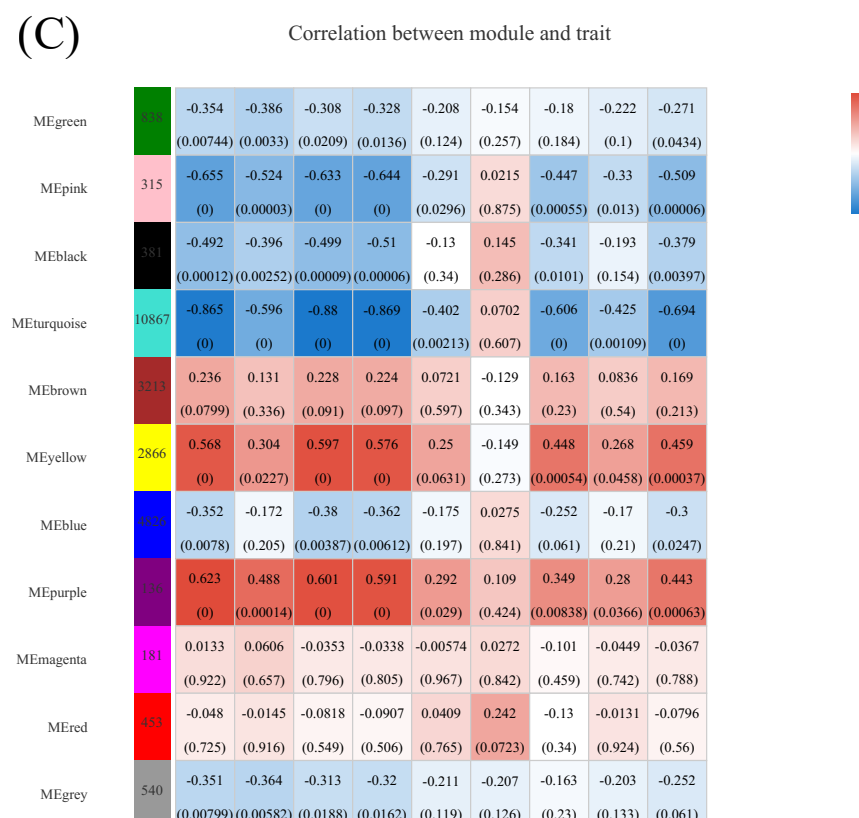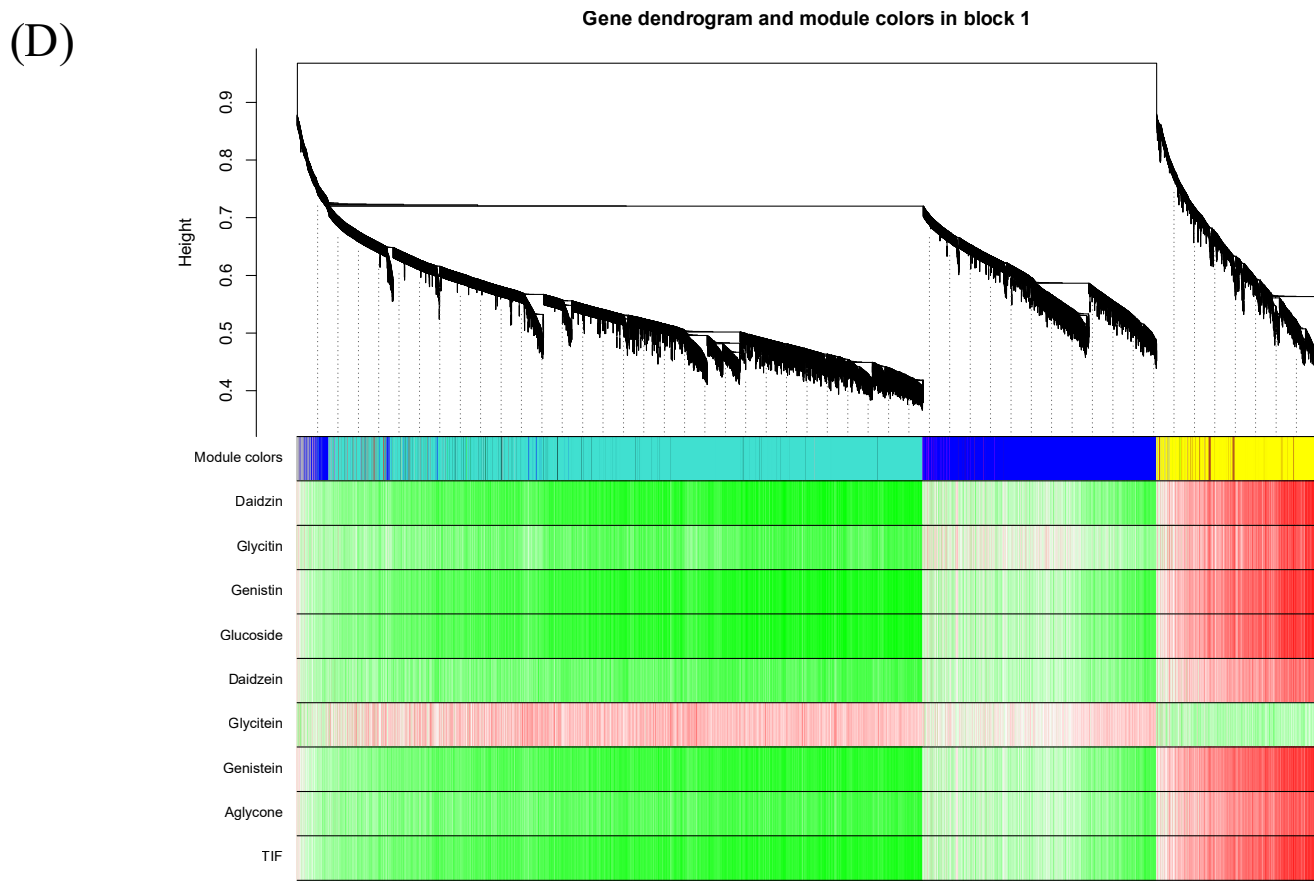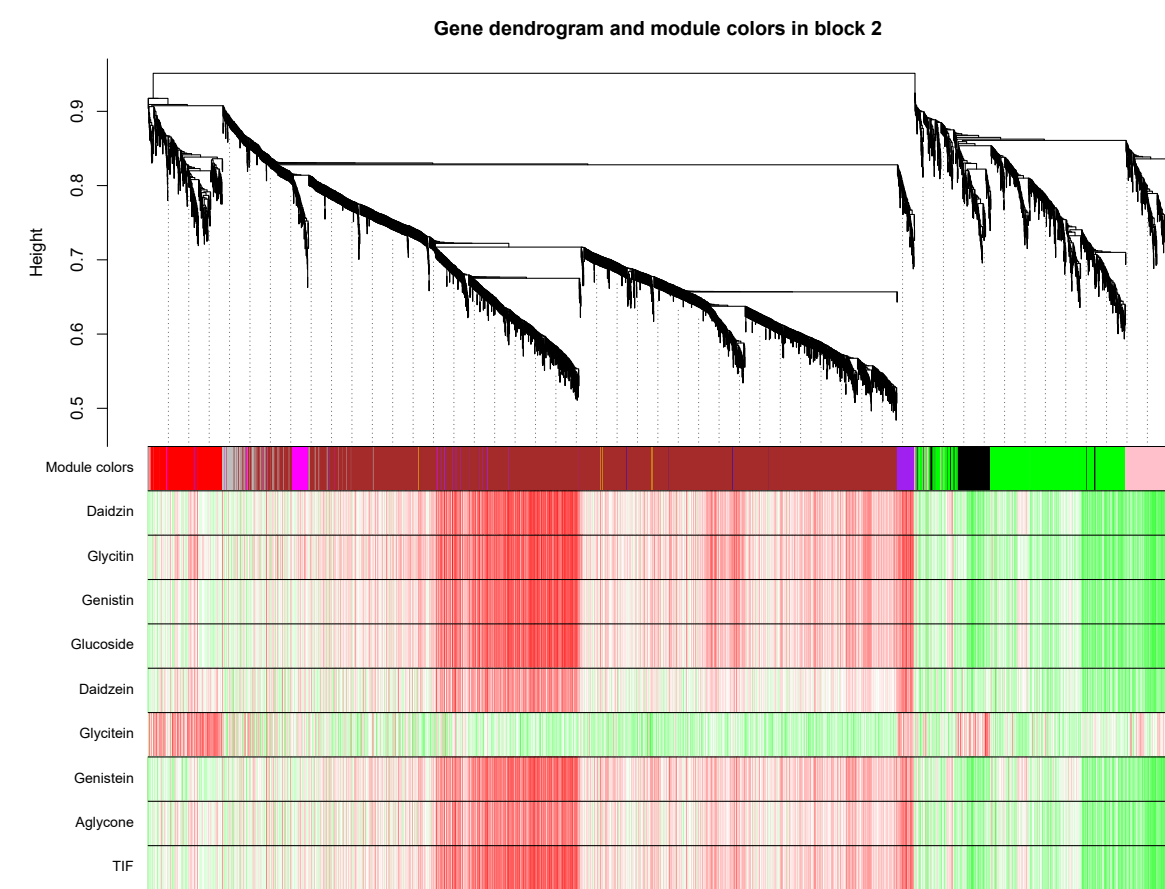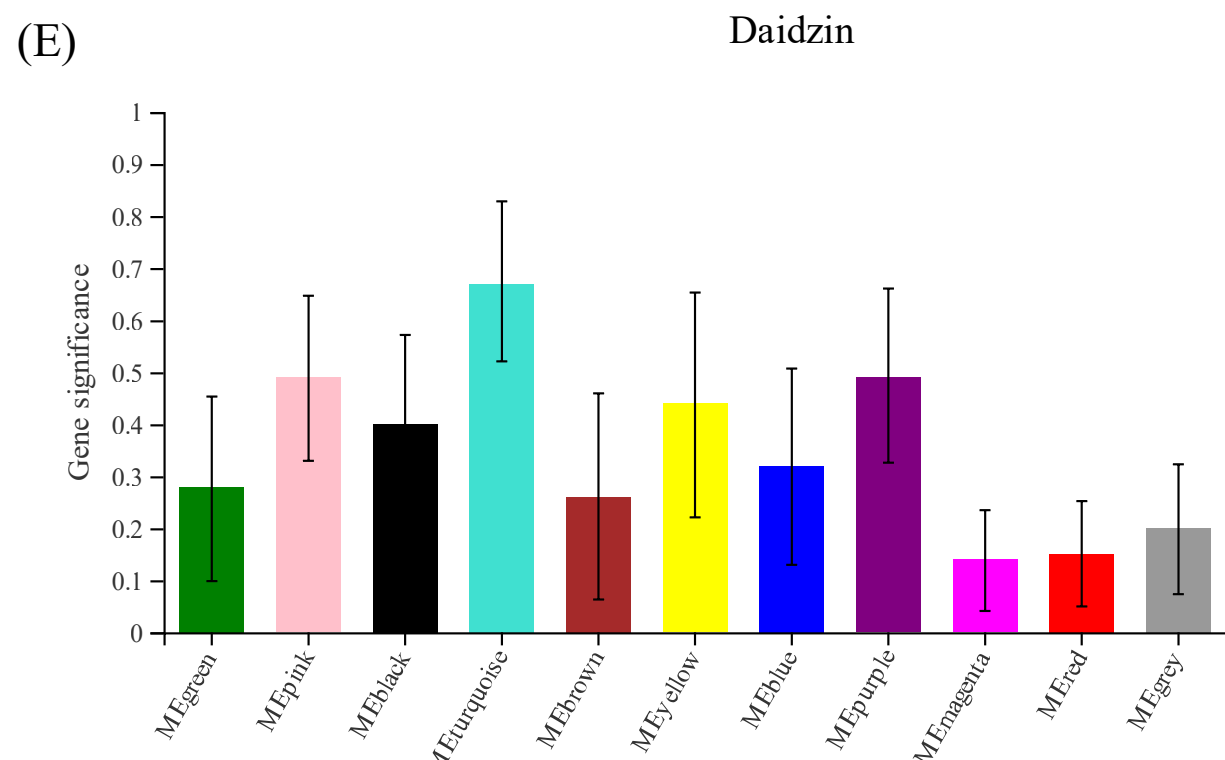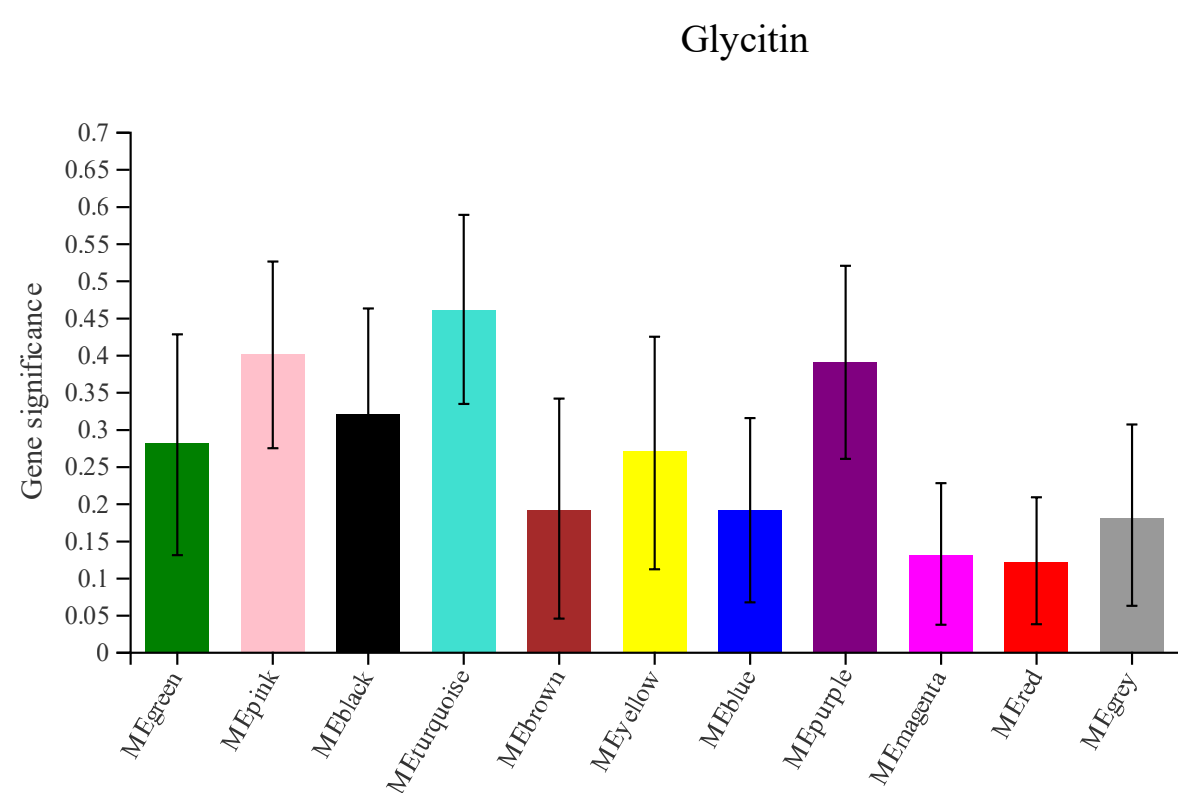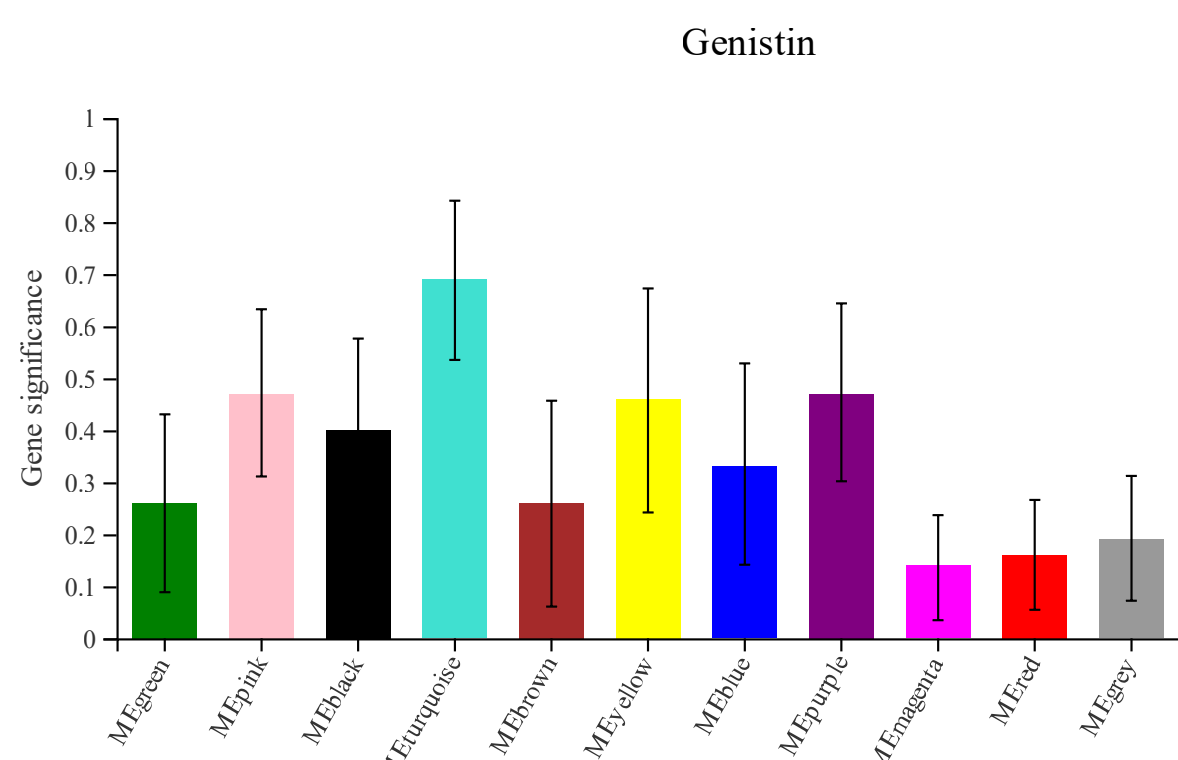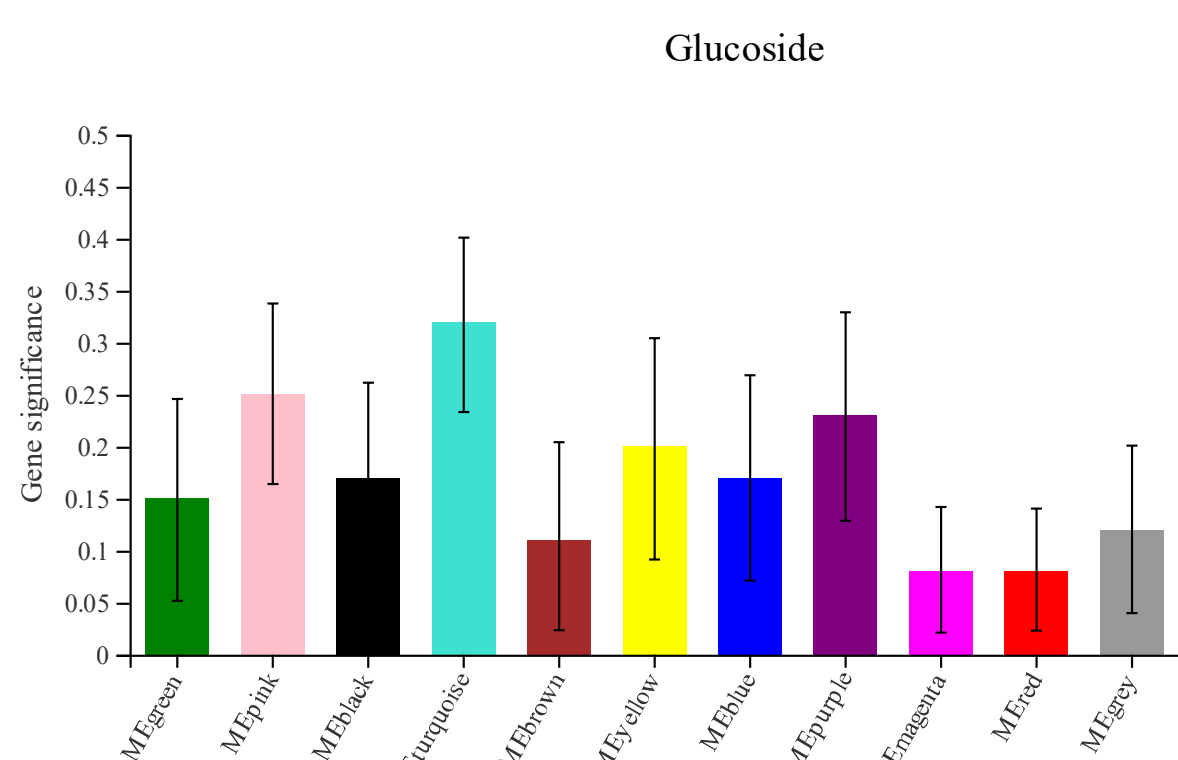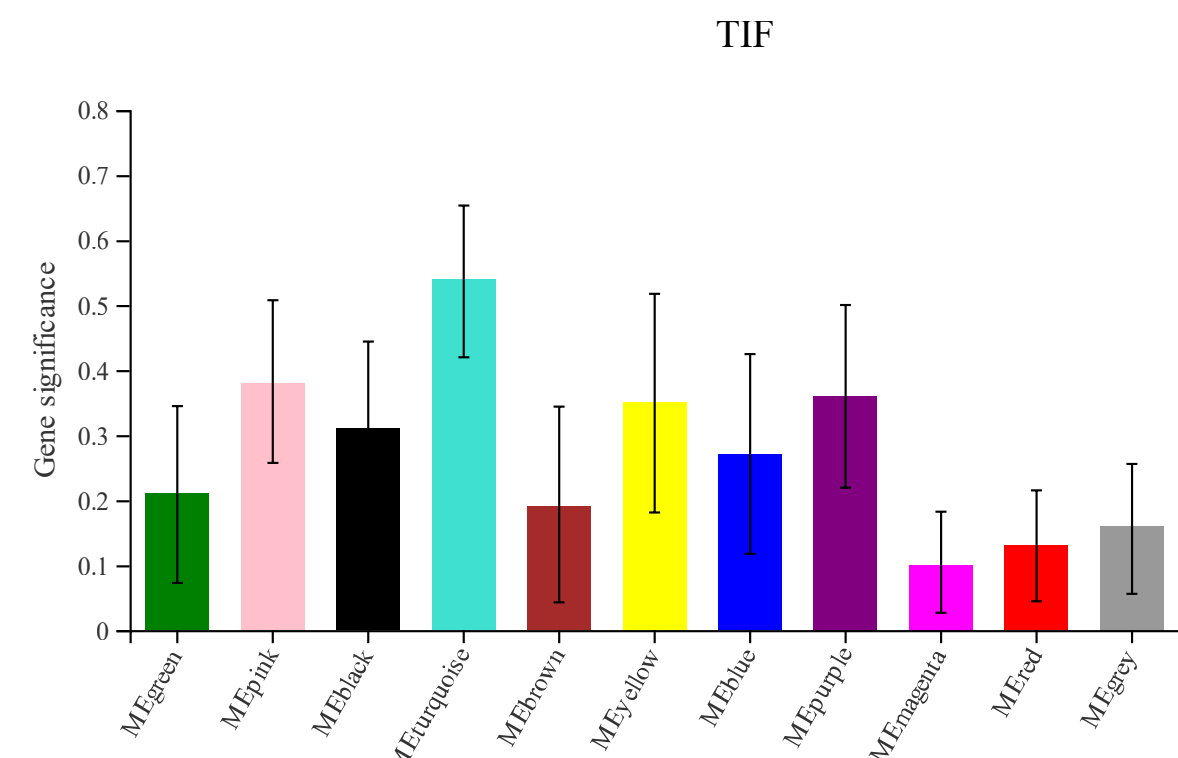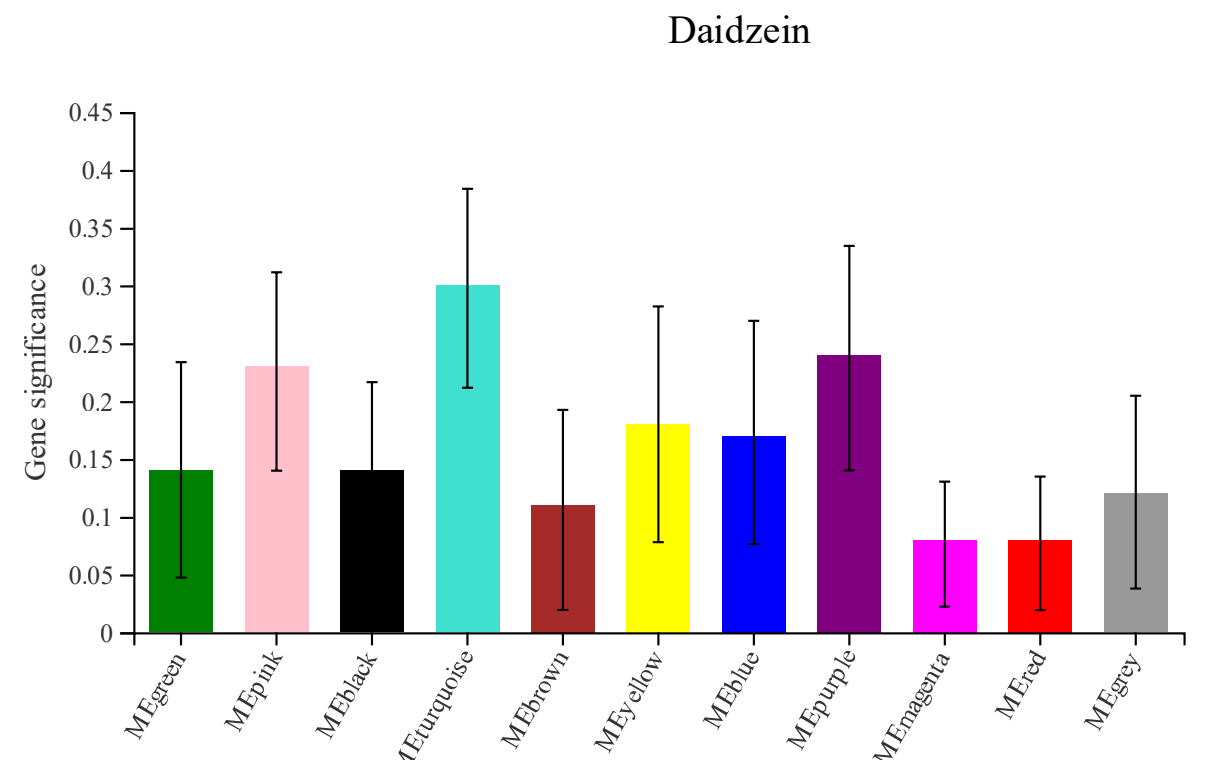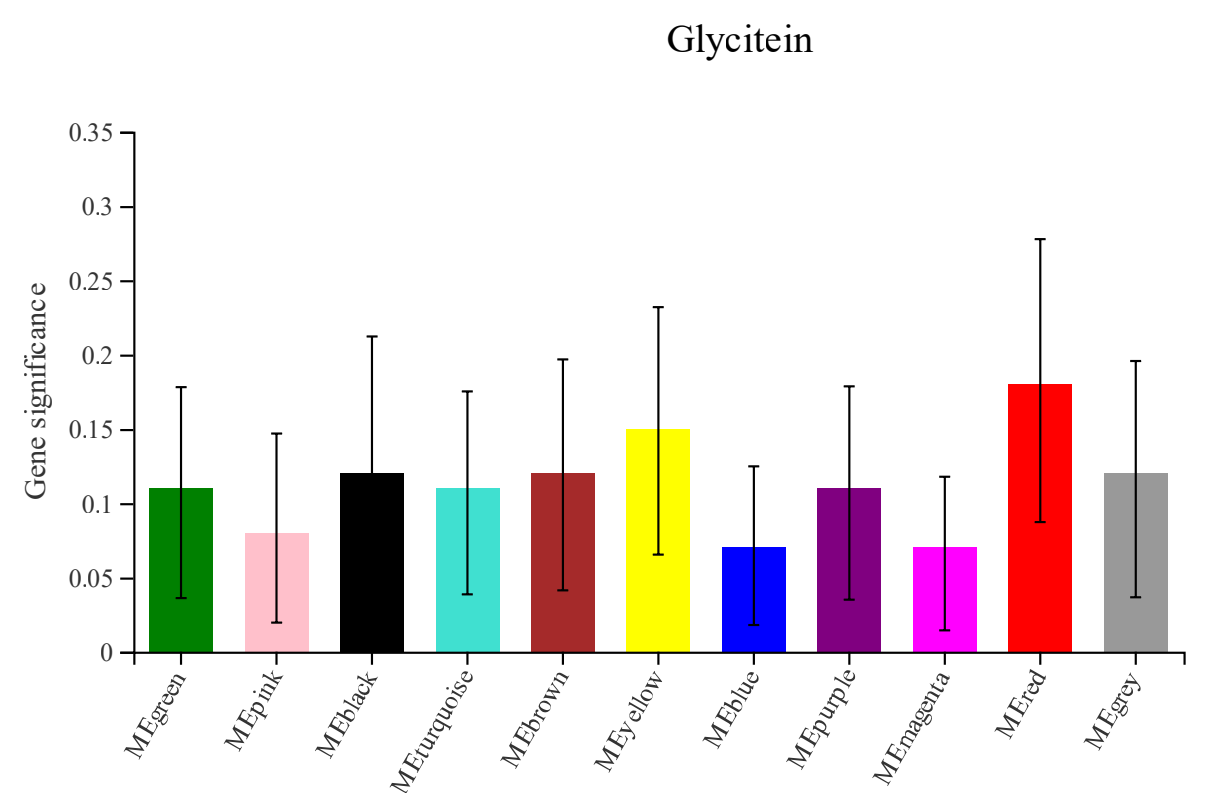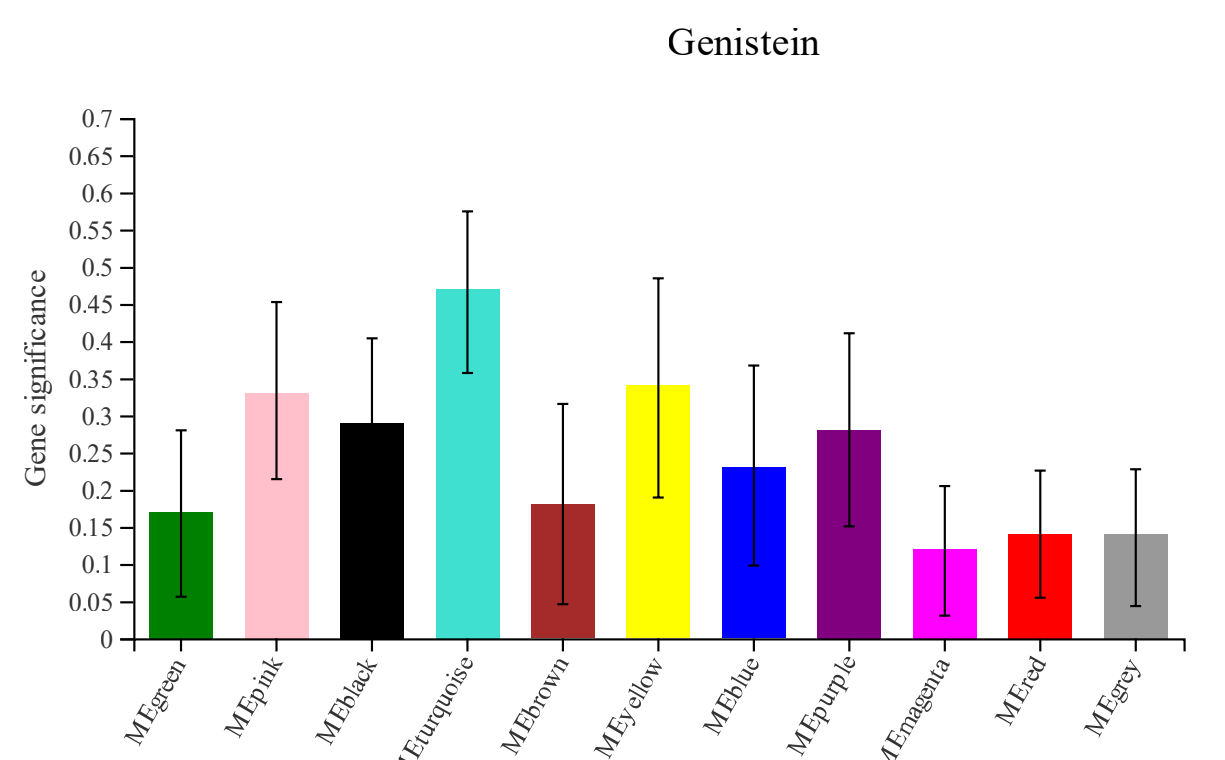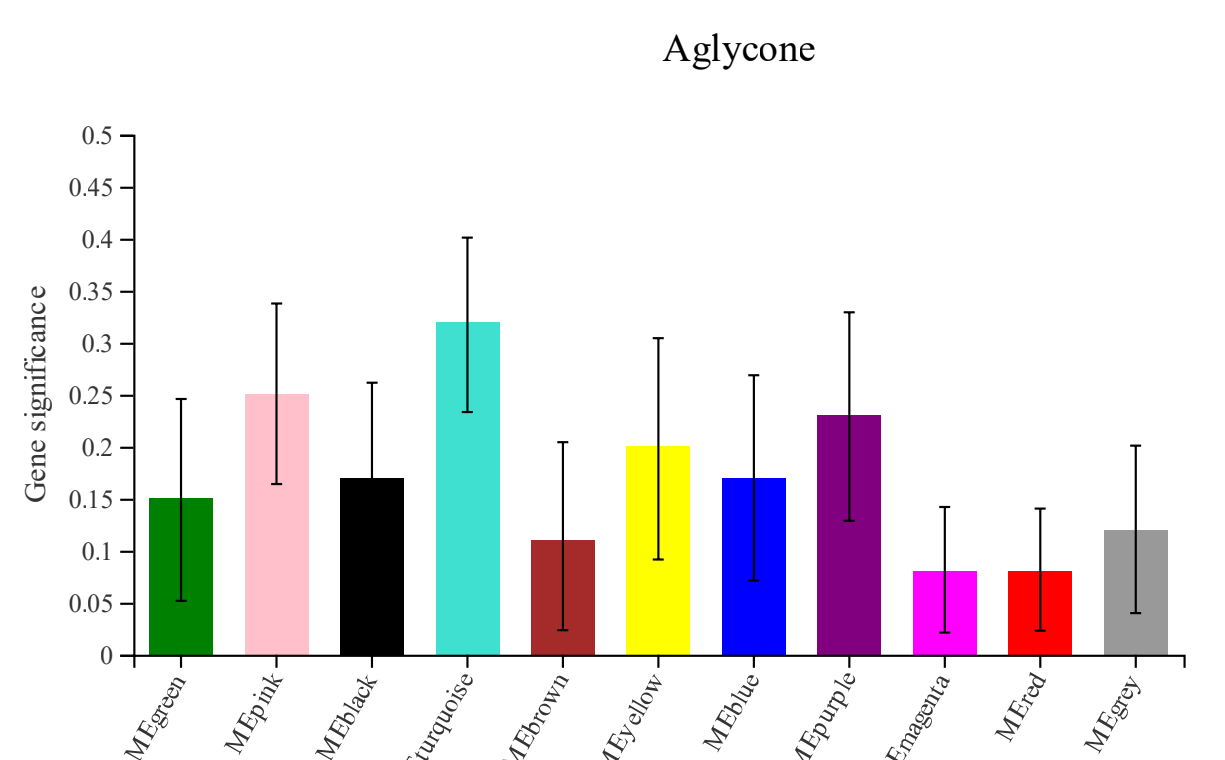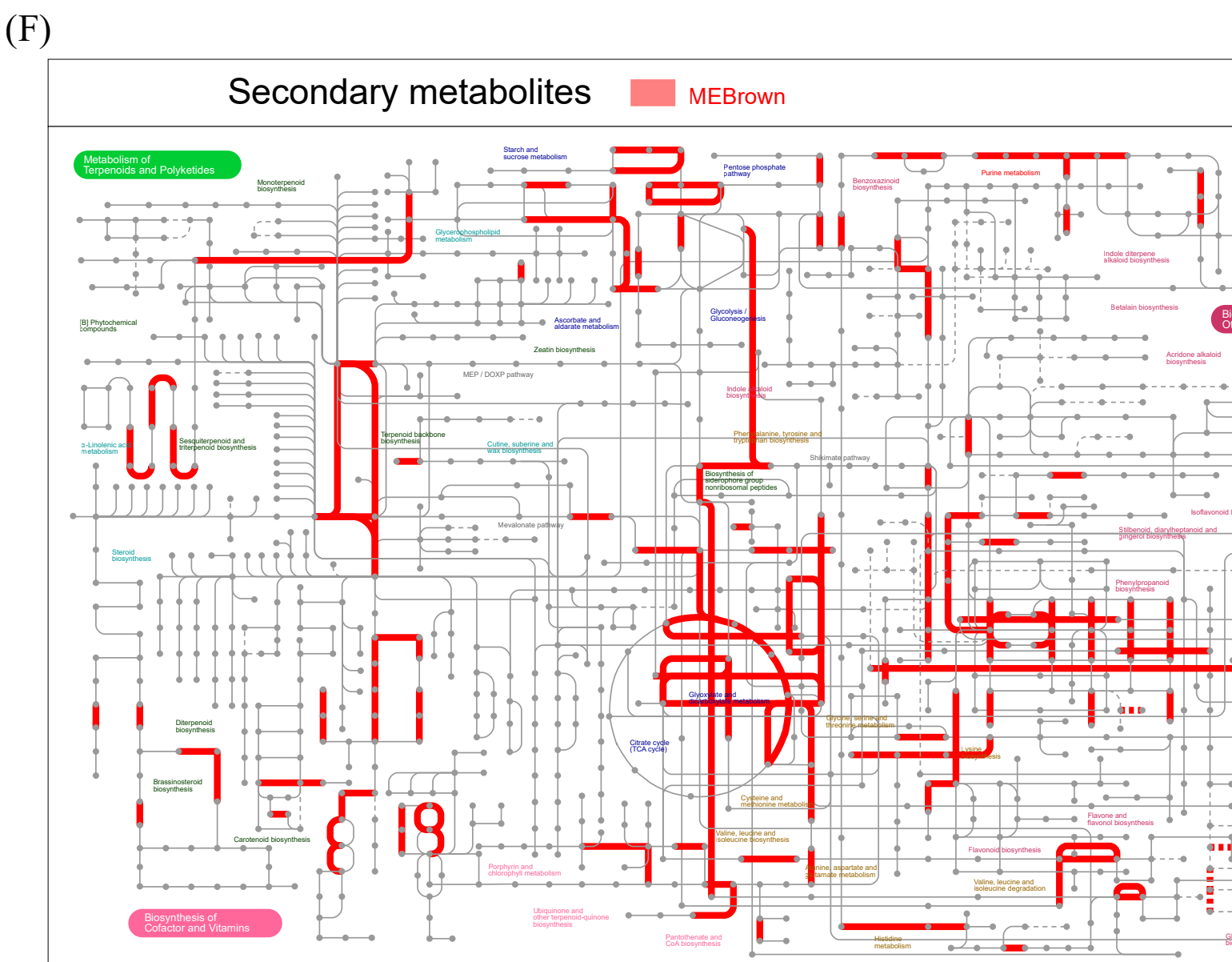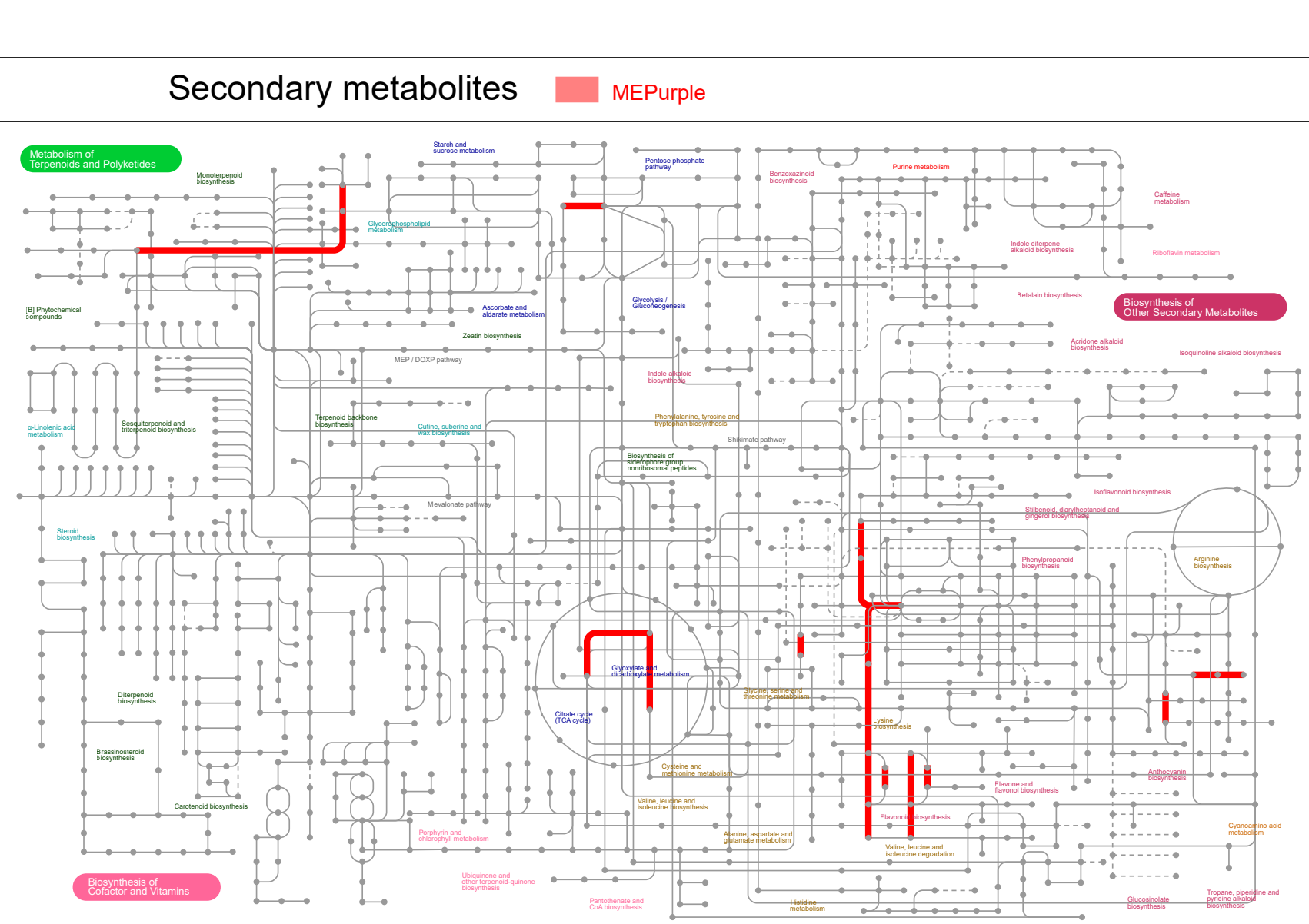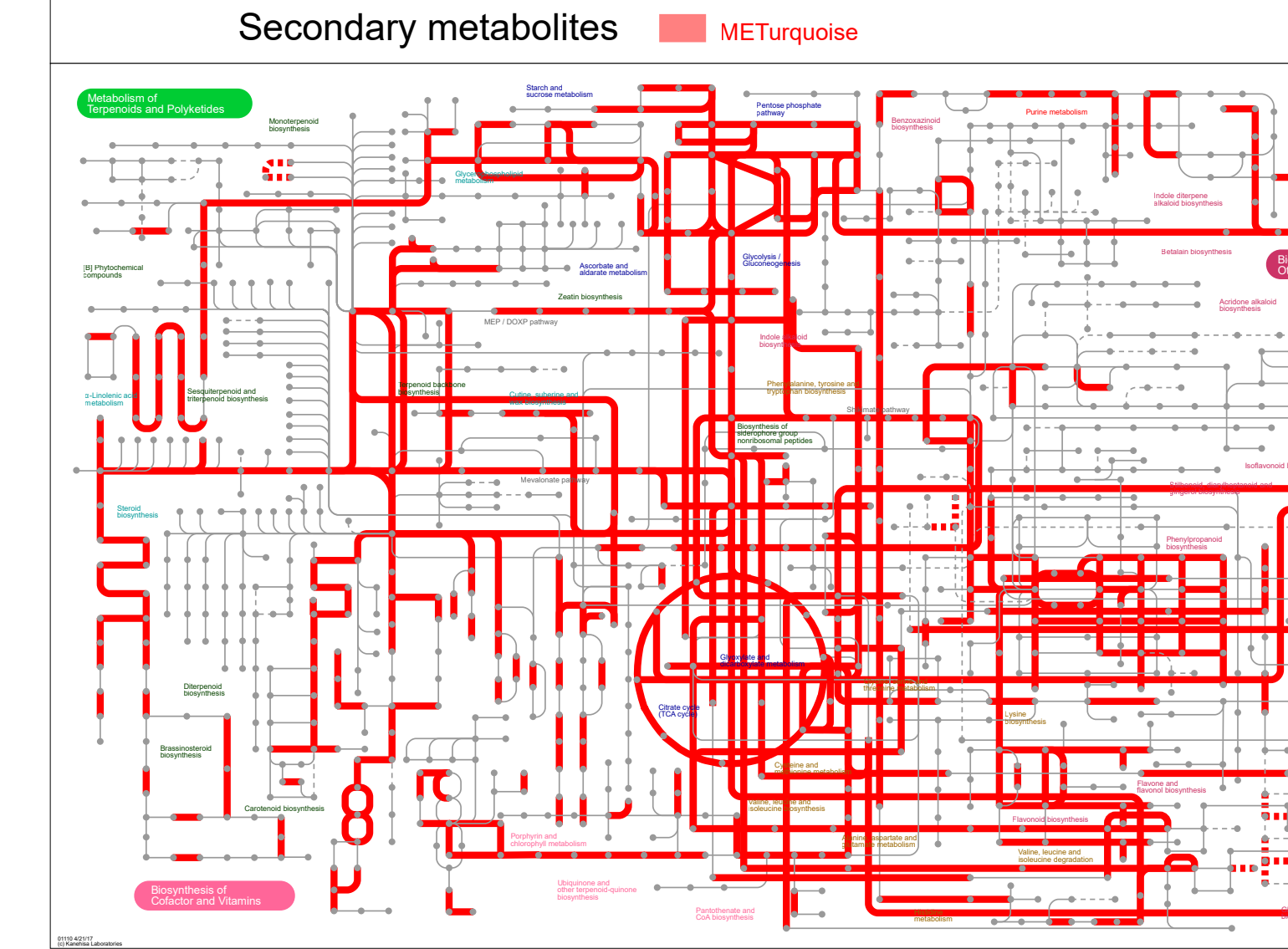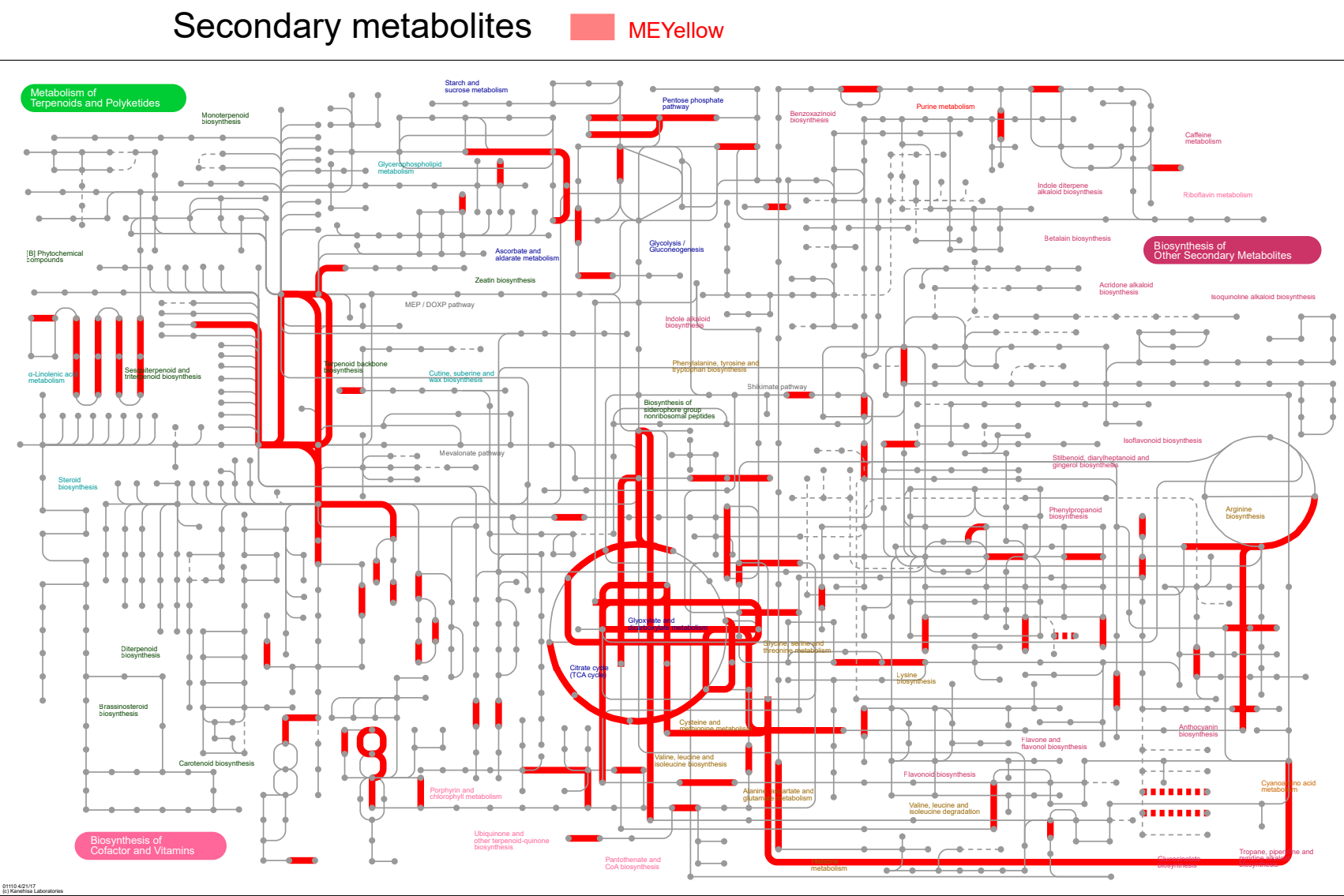

Supplement: Supplementary Figure 5 — Results of WGCNA in this study (A) Gene dendrogram and module colors; (B) Correlation between modules; (C) Correlation between module and trait; (D) Correlation between gene and trait; (E) Module significance (GS) between module and trait; (F) Metabolite pathways of molecular module. [file DataSheet_5.pdf]

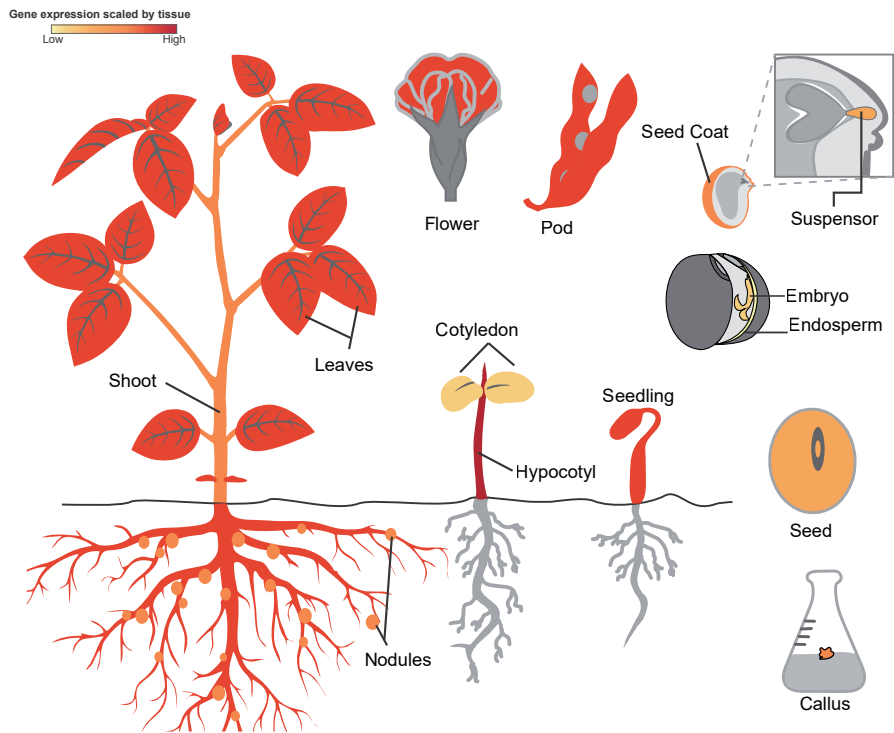

*Gm4CL1* (Glyma.15G001700)

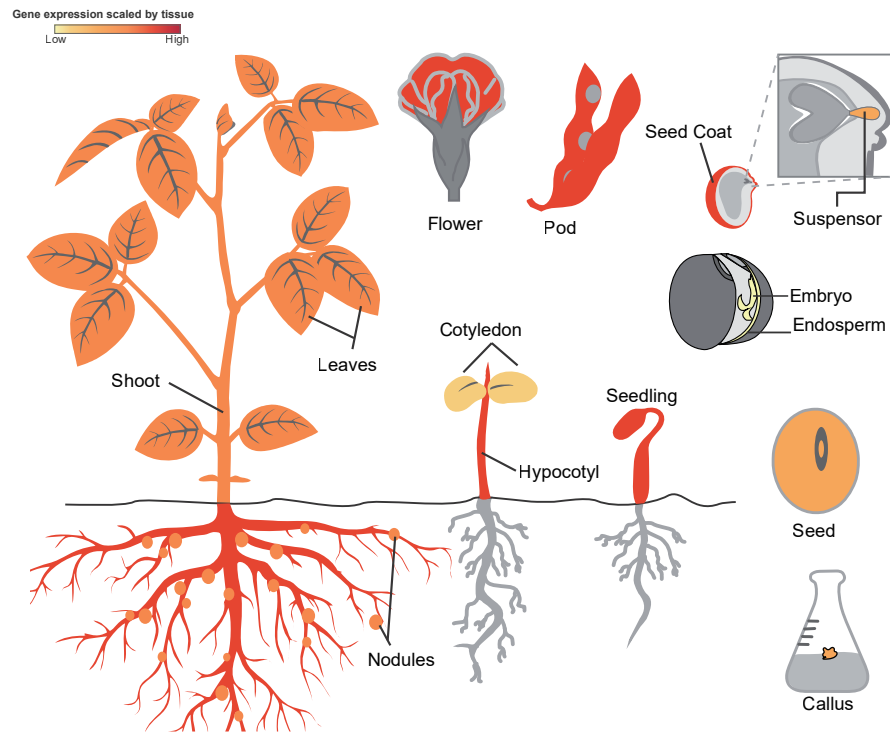

*Gm4CL2* (Glyma.13G372000)

Supplement: Supplementary Figure 11 — Expression level of Gm4CL1 and Gm4CL2 in various tissues. [file DataSheet_11.pdf]
