# Supplementary material for: Developmental dynamic transcriptome and systematic analysis reveal the major genes underlying isoflavone accumulation in soybean
Source: Front Plant Sci. 2023 Mar 7;14:1014349. doi: 10.3389/fpls.2023.1014349 (PMC10027745; doi:10.3389/fpls.2023.1014349)
Supplement: Supplementary Figure 6 — Results of time series analysis. [file DataSheet_6.pdf]

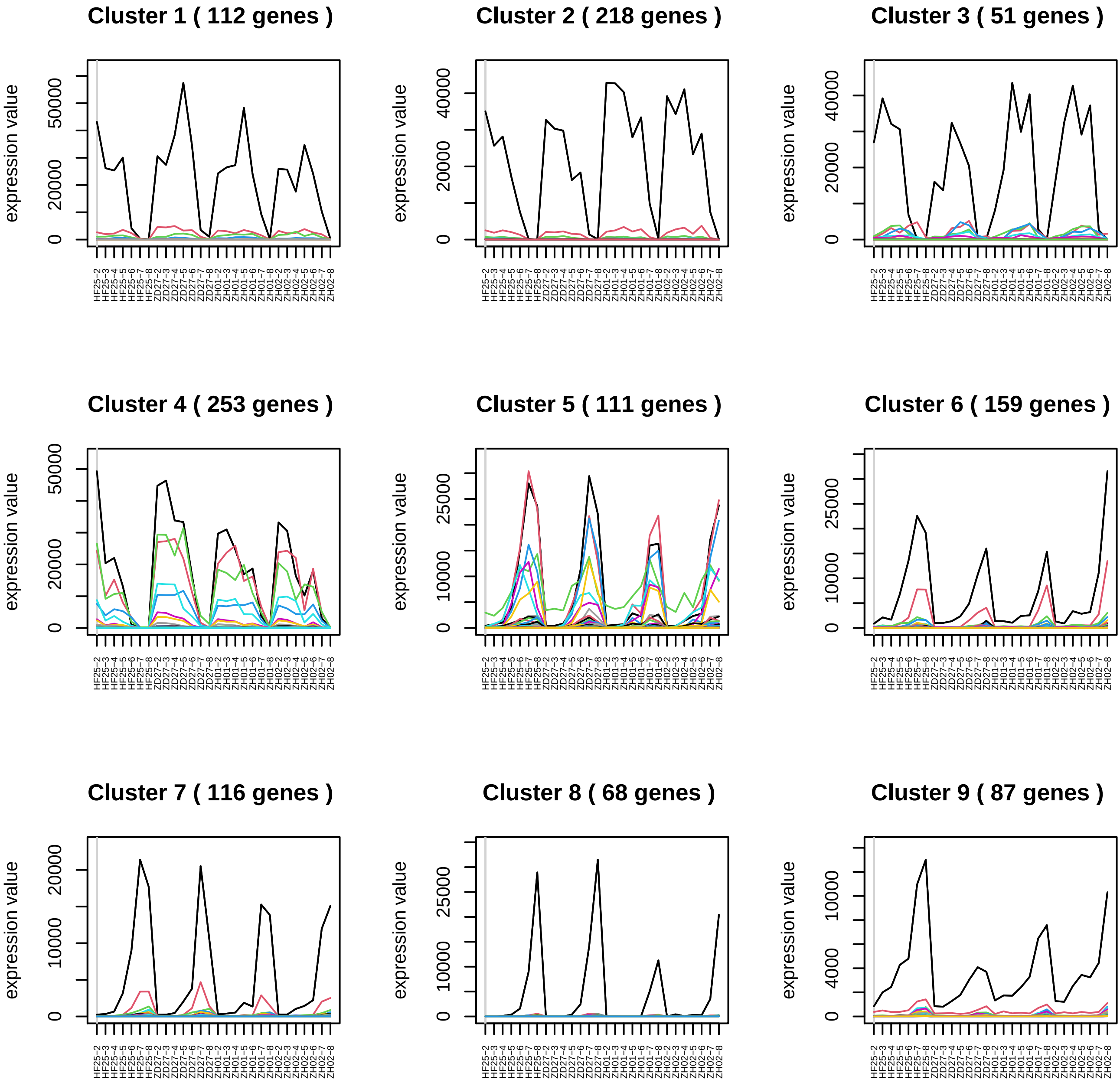
Cluster 1 (112 genes)
expression value
0
20000
50000
Cluster 2 (218 genes)
expression value
0
20000
40000
Cluster 3 (51 genes)
expression value
0
20000
40000
Cluster 4 (253 genes)
expression value
0
20000
50000
Cluster 5 (111 genes)
expression value
0
10000
25000
Cluster 6 (159 genes)
expression value
0
10000
25000
Cluster 7 (116 genes)
expression value
0
10000
20000
Cluster 8 (68 genes)
expression value
0
10000
25000
Cluster 9 (87 genes)
expression value
0
4000
10000
HF25-2 HF25-3 HF25-4 HF25-5 HF25-6 HF25-7 HF25-8 ZD27-2 ZD27-3 ZD27-4 ZD27-5 ZD27-6 ZD27-7 ZD27-8 ZH01-2 ZH01-3 ZH01-4 ZH01-5 ZH01-6 ZH01-7 ZH01-8 ZH02-2 ZH02-3 ZH02-4 ZH02-5 ZH02-6 ZH02-7 ZH02-8

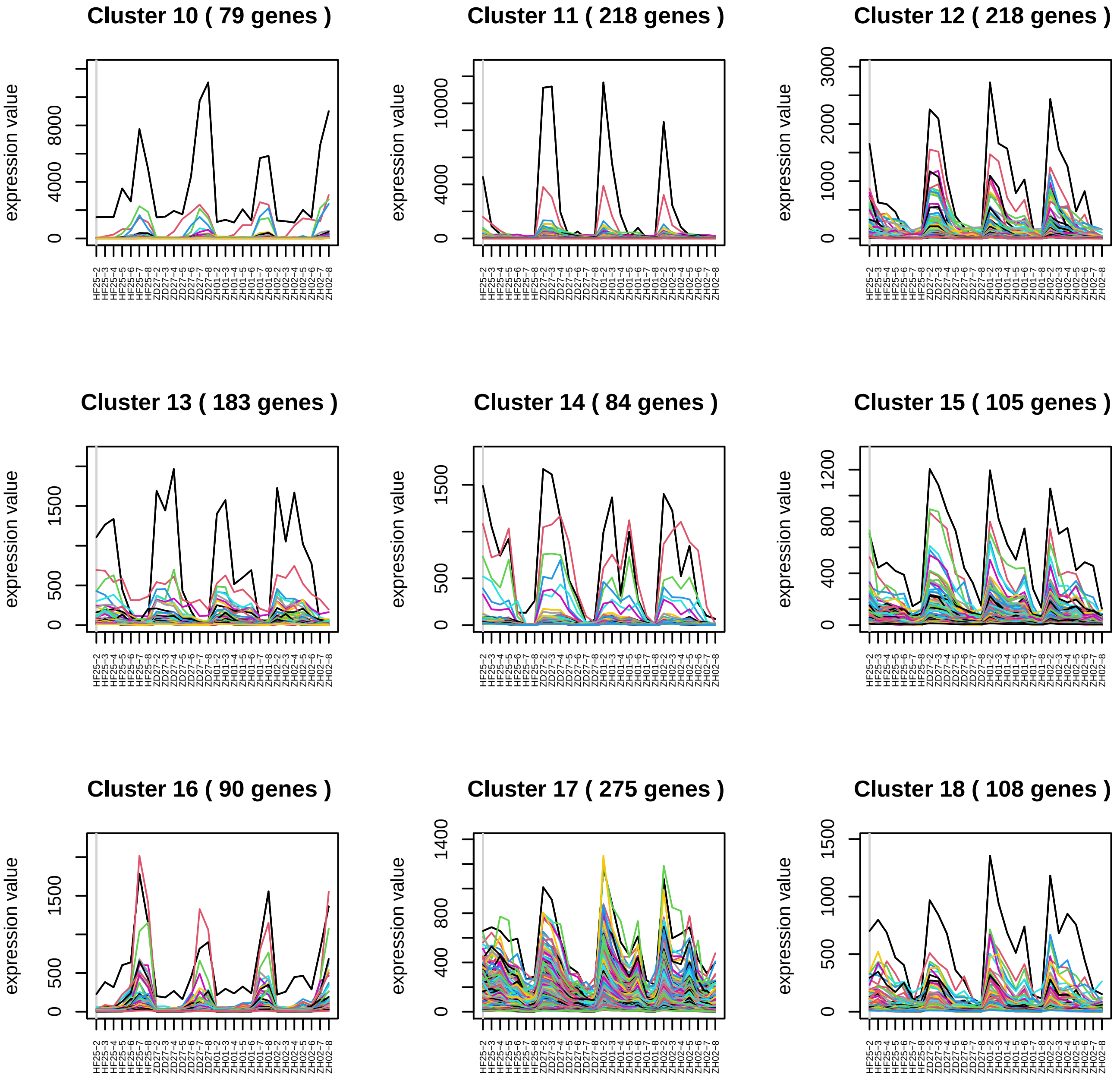
Cluster 10 (79 genes)
Cluster 11 (218 genes)
Cluster 12 (218 genes)
Cluster 13 (183 genes)
Cluster 14 (84 genes)
Cluster 15 (105 genes)
Cluster 16 (90 genes)
Cluster 17 (275 genes)
Cluster 18 (108 genes)
expression value
HF25-2 HF25-3 HF25-4 HF25-5 HF25-6 HF25-7 HF25-8
ZD27-2 ZD27-3 ZD27-4 ZD27-5 ZD27-6 ZD27-7 ZD27-8
ZH01-2 ZH01-3 ZH01-4 ZH01-5 ZH01-6 ZH01-7 ZH01-8
ZH02-2 ZH02-3 ZH02-4 ZH02-5 ZH02-6 ZH02-7 ZH02-8

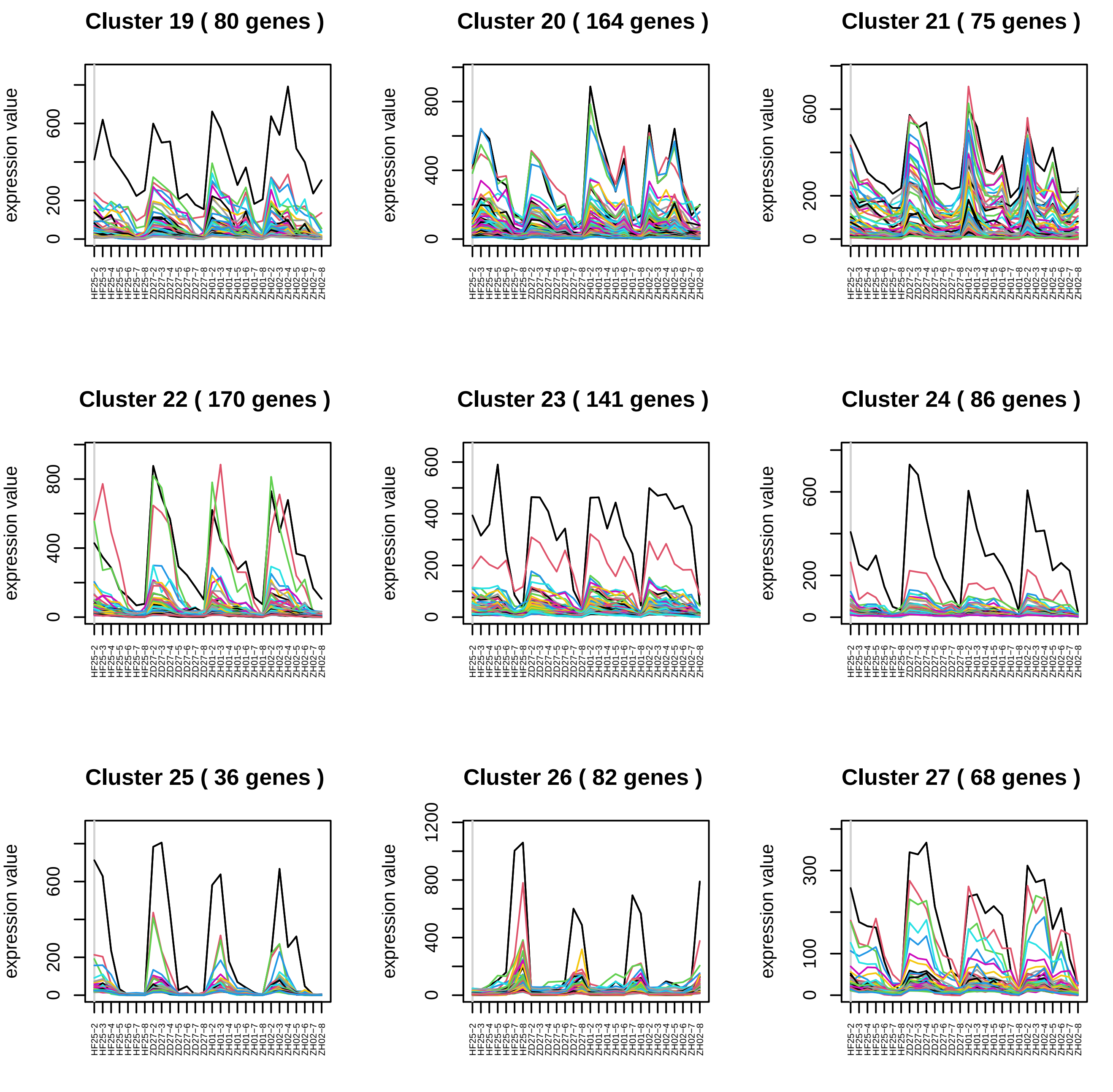
Cluster 19 (80 genes)
Cluster 20 (164 genes)
Cluster 21 (75 genes)
Cluster 22 (170 genes)
Cluster 23 (141 genes)
Cluster 24 (86 genes)
Cluster 25 (36 genes)
Cluster 26 (82 genes)
Cluster 27 (68 genes)
expression value
HF25-2 HF25-3 HF25-4 HF25-5 HF25-6 HF25-7 HF25-8 ZD27-2 ZD27-3 ZD27-4 ZD27-5 ZD27-6 ZD27-7 ZD27-8 ZH01-2 ZH01-3 ZH01-4 ZH01-5 ZH01-6 ZH01-7 ZH01-8 ZH02-2 ZH02-3 ZH02-4 ZH02-5 ZH02-6 ZH02-7 ZH02-8

Cluster 28 (32 genes)

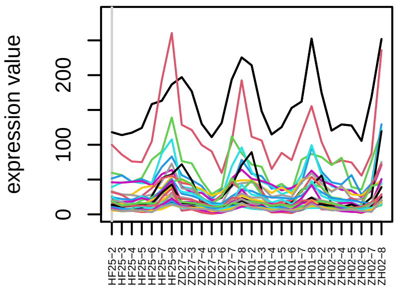

Cluster 29 (73 genes)

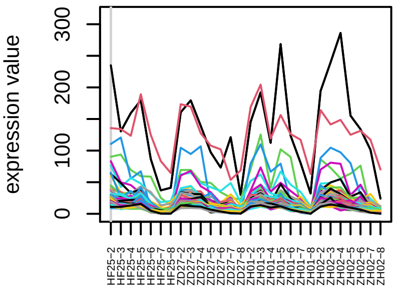

Cluster 30 (58 genes)

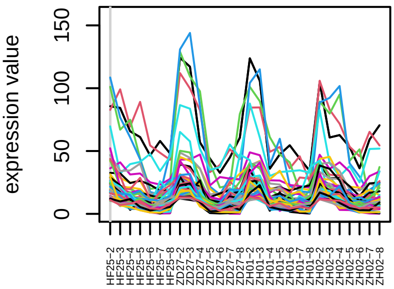

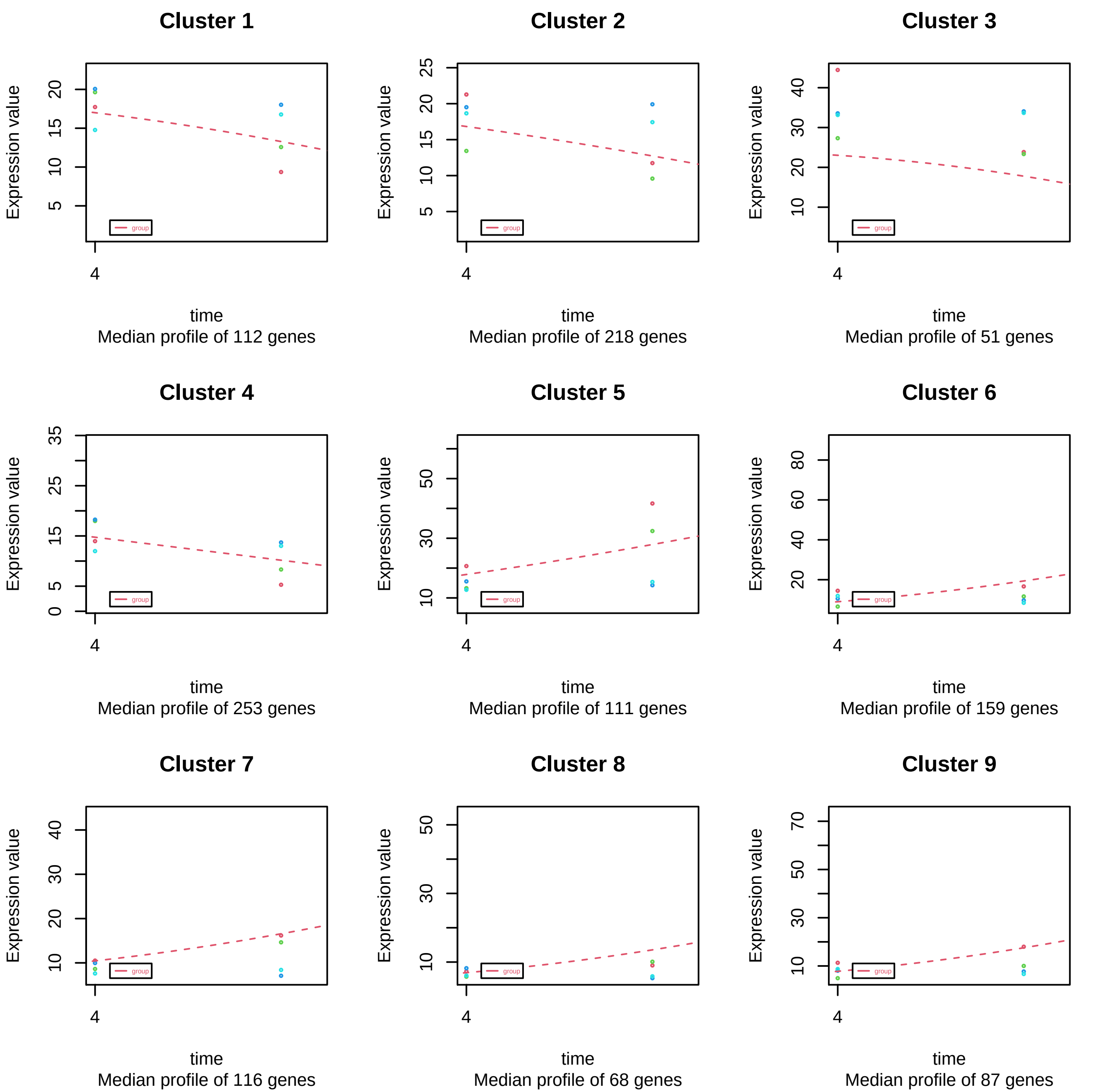
Cluster 1
Expression value
5
10
15
20
group
4
time
Median profile of 112 genes
Cluster 2
Expression value
5
10
15
20
25
group
4
time
Median profile of 218 genes
Cluster 3
Expression value
10
20
30
40
group
4
time
Median profile of 51 genes
Cluster 4
Expression value
0
5
15
25
35
group
4
time
Median profile of 253 genes
Cluster 5
Expression value
10
30
50
group
4
time
Median profile of 111 genes
Cluster 6
Expression value
20
40
60
80
group
4
time
Median profile of 159 genes
Cluster 7
Expression value
10
20
30
40
group
4
time
Median profile of 116 genes
Cluster 8
Expression value
10
30
50
group
4
time
Median profile of 68 genes
Cluster 9
Expression value
10
30
50
70
group
4
time
Median profile of 87 genes

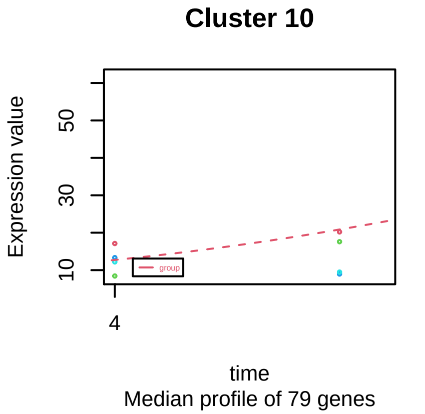
Cluster 10
Expression value
10
30
50
4
group
time
Median profile of 79 genes

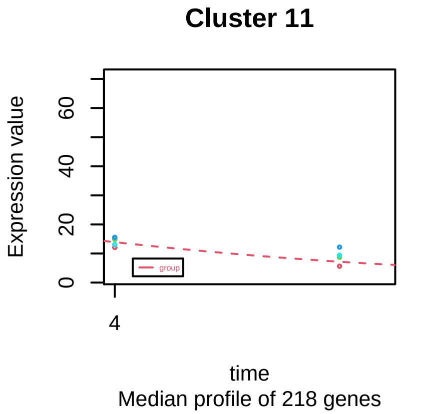
Cluster 11
Expression value
0
20
40
60
4
group
time
Median profile of 218 genes

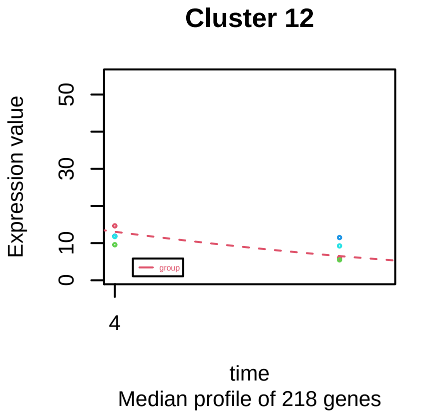
Cluster 12
Expression value
0
10
30
50
4
group
time
Median profile of 218 genes

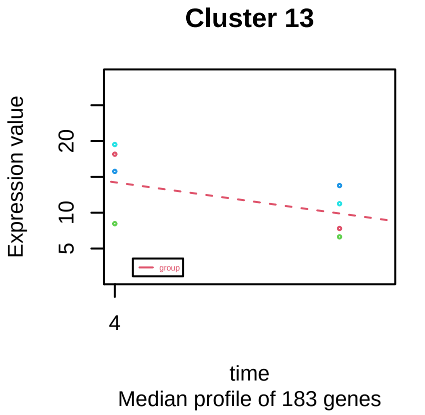
Cluster 13
Expression value
5
10
20
4
group
time
Median profile of 183 genes

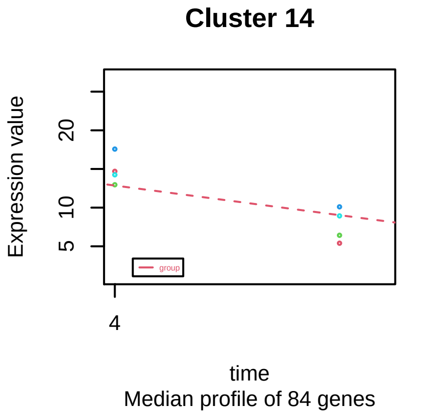
Cluster 14
Expression value
5
10
20
4
group
time
Median profile of 84 genes

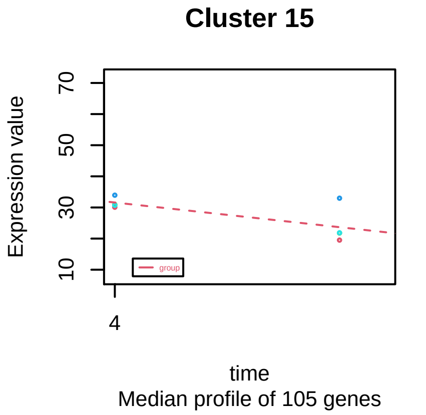
Cluster 15
Expression value
10
30
50
70
4
group
time
Median profile of 105 genes

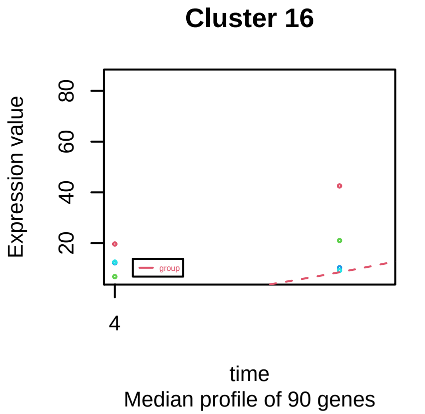
Cluster 16
Expression value
20
40
60
80
4
group
time
Median profile of 90 genes

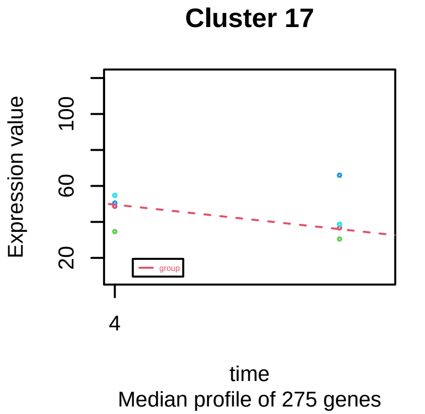
Cluster 17
Expression value
20
60
100
4
group
time
Median profile of 275 genes

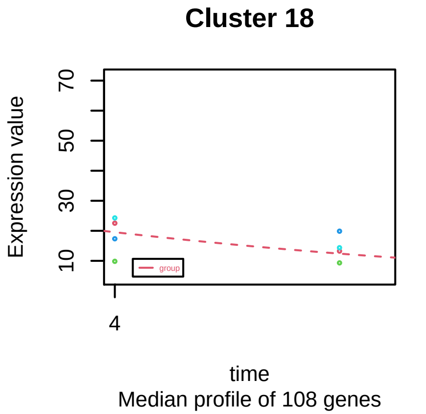
Cluster 18
Expression value
10
30
50
70
4
group
time
Median profile of 108 genes

Cluster 19

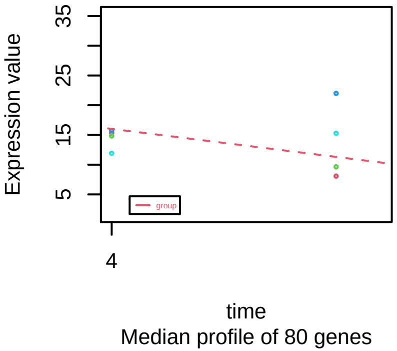

Cluster 20

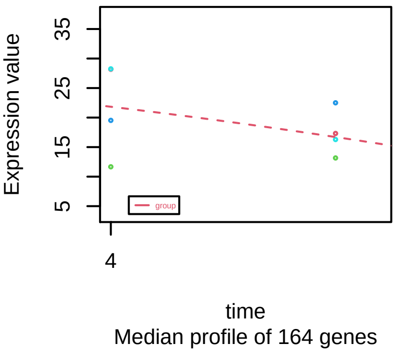

Cluster 21

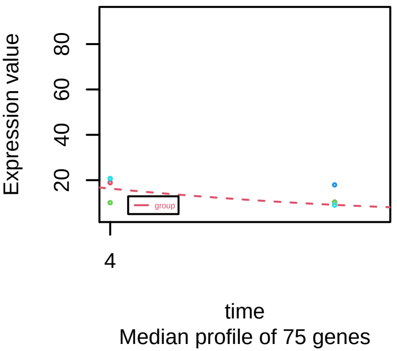

Cluster 22

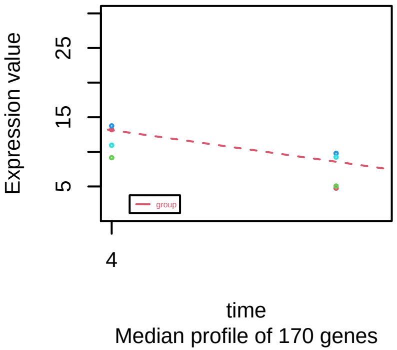

Cluster 23

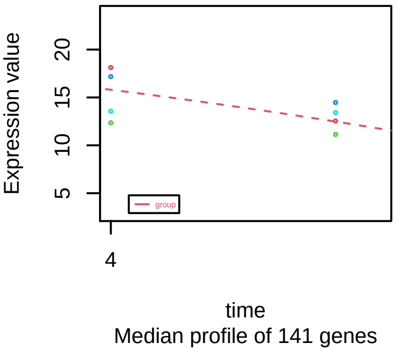

Cluster 24

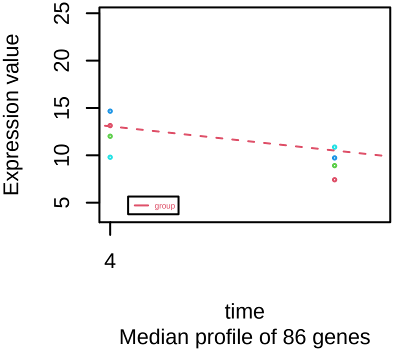

Cluster 25

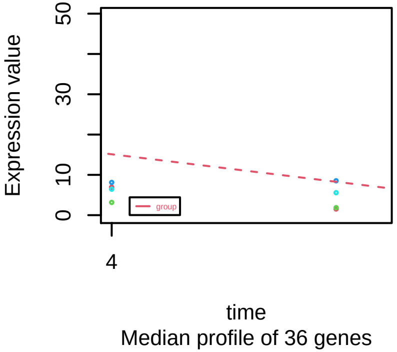

Cluster 26

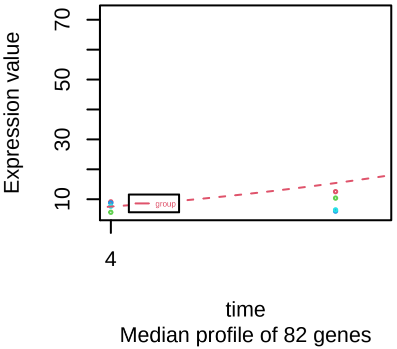

Cluster 27

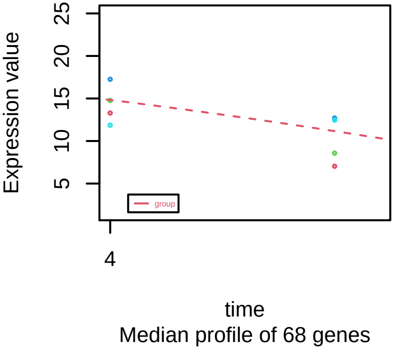

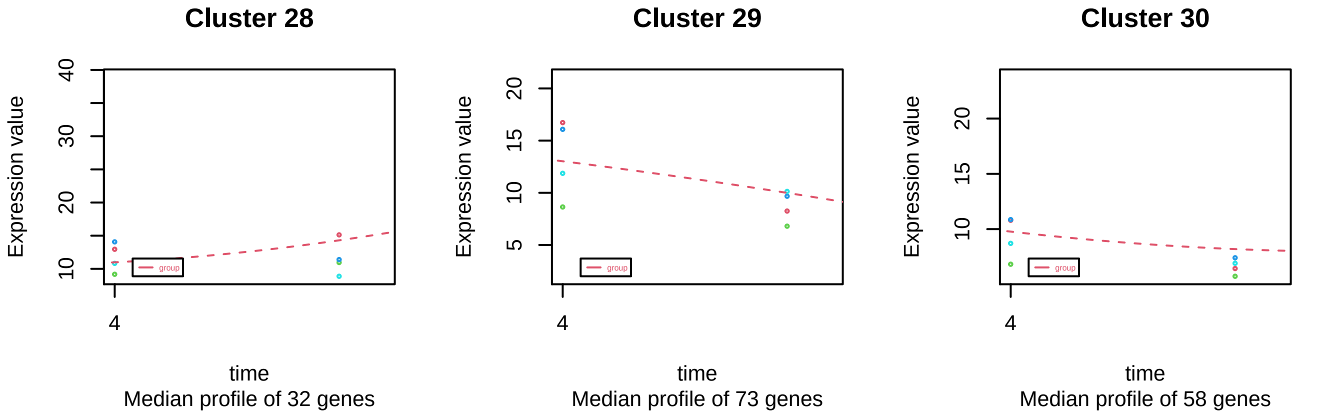

Cluster 28
Expression value
10
20
30
40
4
group
time
Median profile of 32 genes
Cluster 29
Expression value
5
10
15
20
4
group
time
Median profile of 73 genes
Cluster 30
Expression value
10
15
20
4
group
time
Median profile of 58 genes
