# Supplementary material for: Developmental dynamic transcriptome and systematic analysis reveal the major genes underlying isoflavone accumulation in soybean
Source: Front Plant Sci. 2023 Mar 7;14:1014349. doi: 10.3389/fpls.2023.1014349 (PMC10027745; doi:10.3389/fpls.2023.1014349)

(A) DE analysis

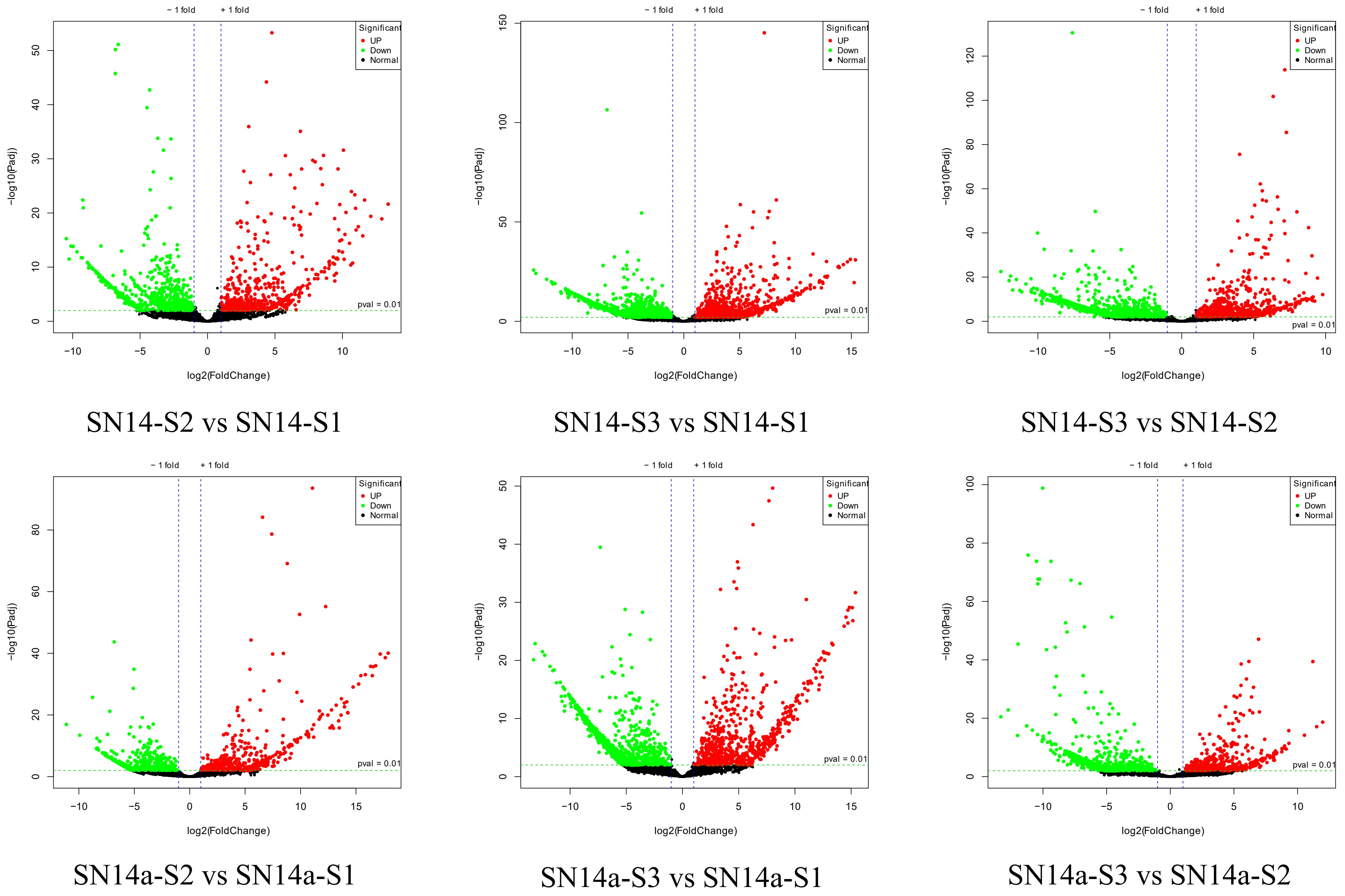

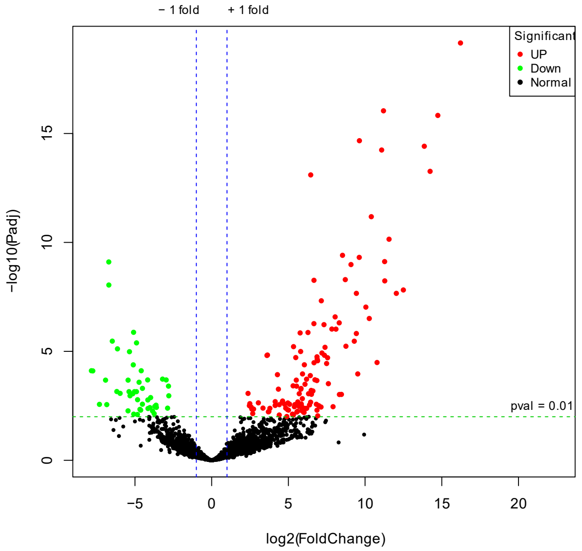

SN14b-S2 vs SN14b-S1

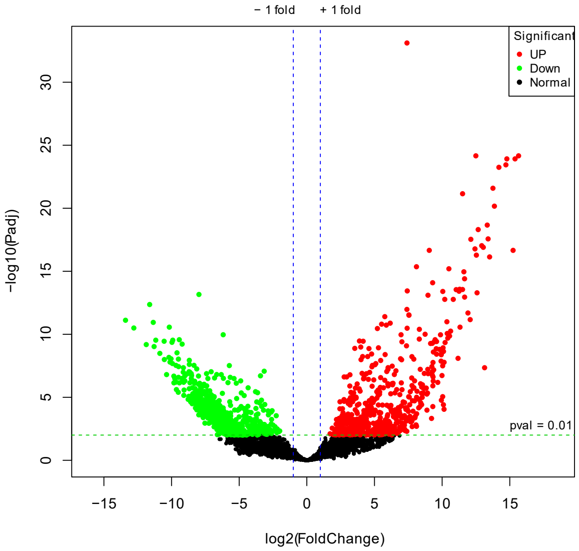

SN14b-S3 vs SN14b-S1

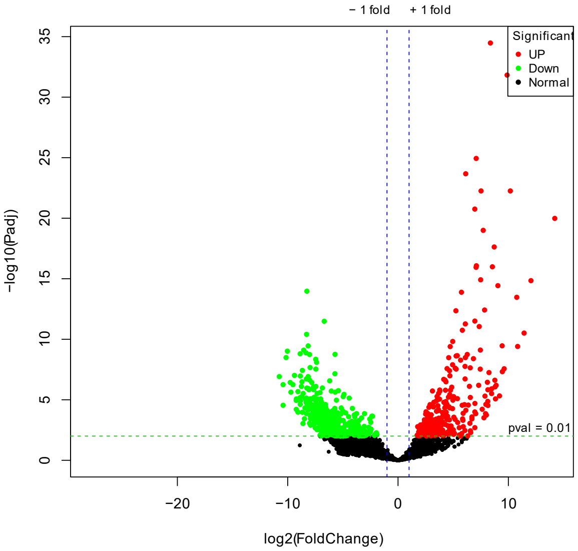

SN14b-S3 vs SN14b-S2

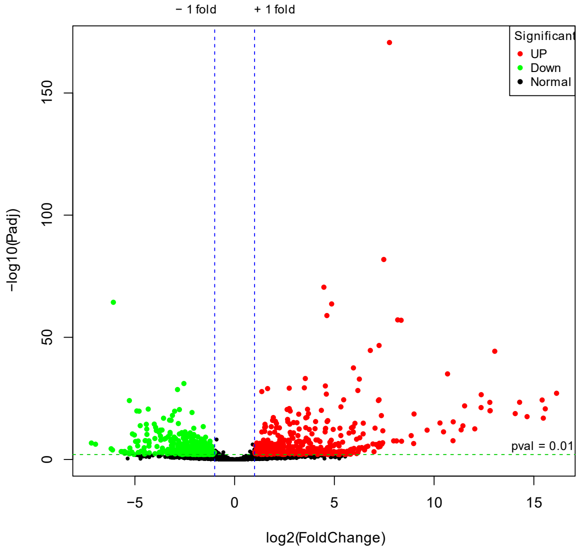

SN14c-S2 vs SN14c-S1

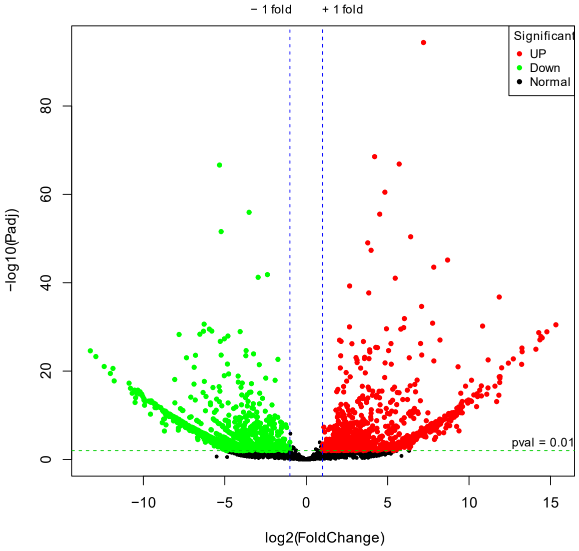

SN14c-S3 vs SN14c-S1

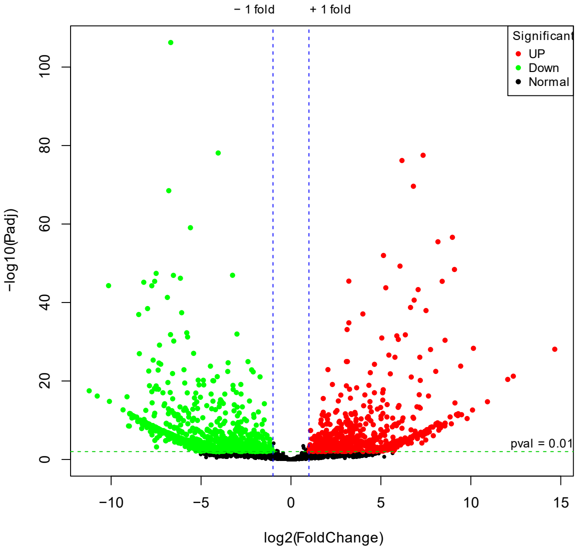

SN14c-S3 vs SN14c-S2

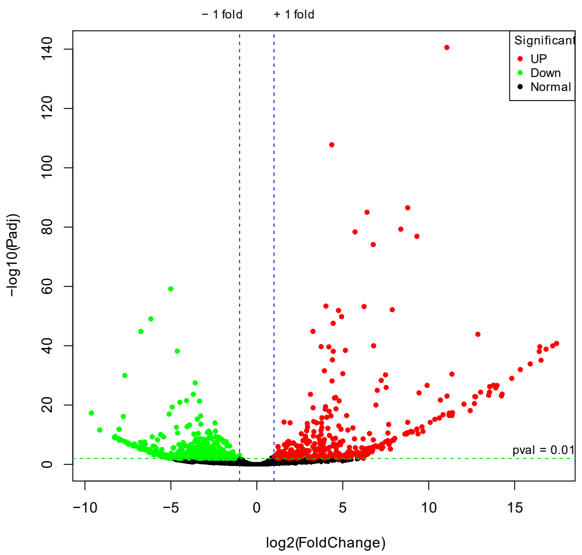

SN14d-S2 vs SN14d-S1

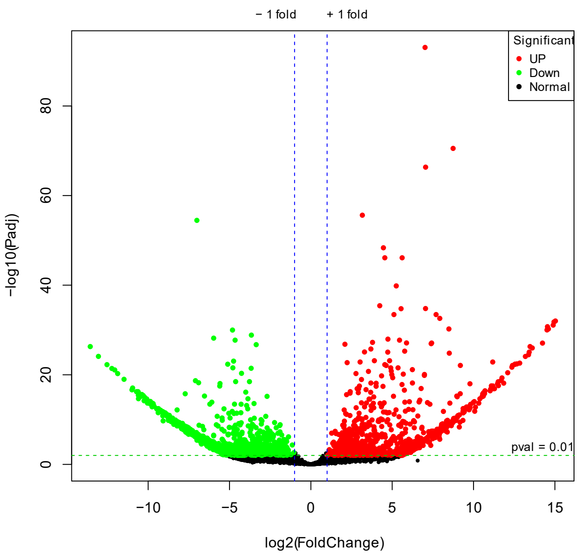

SN14d-S3 vs SN14d-S1

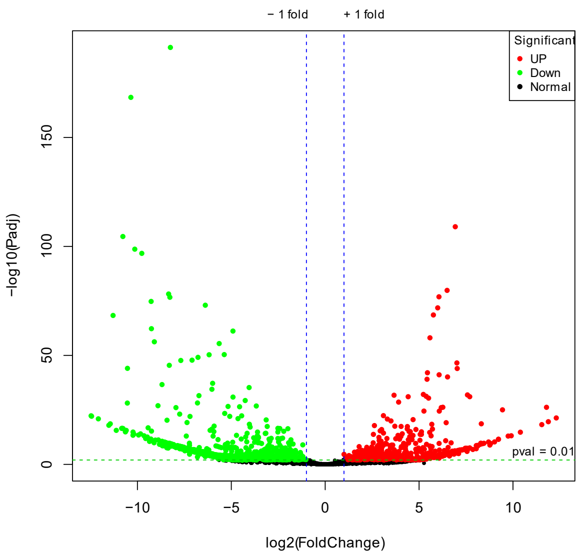

SN14d-S3 vs SN14d-S2

(B) WGCNA

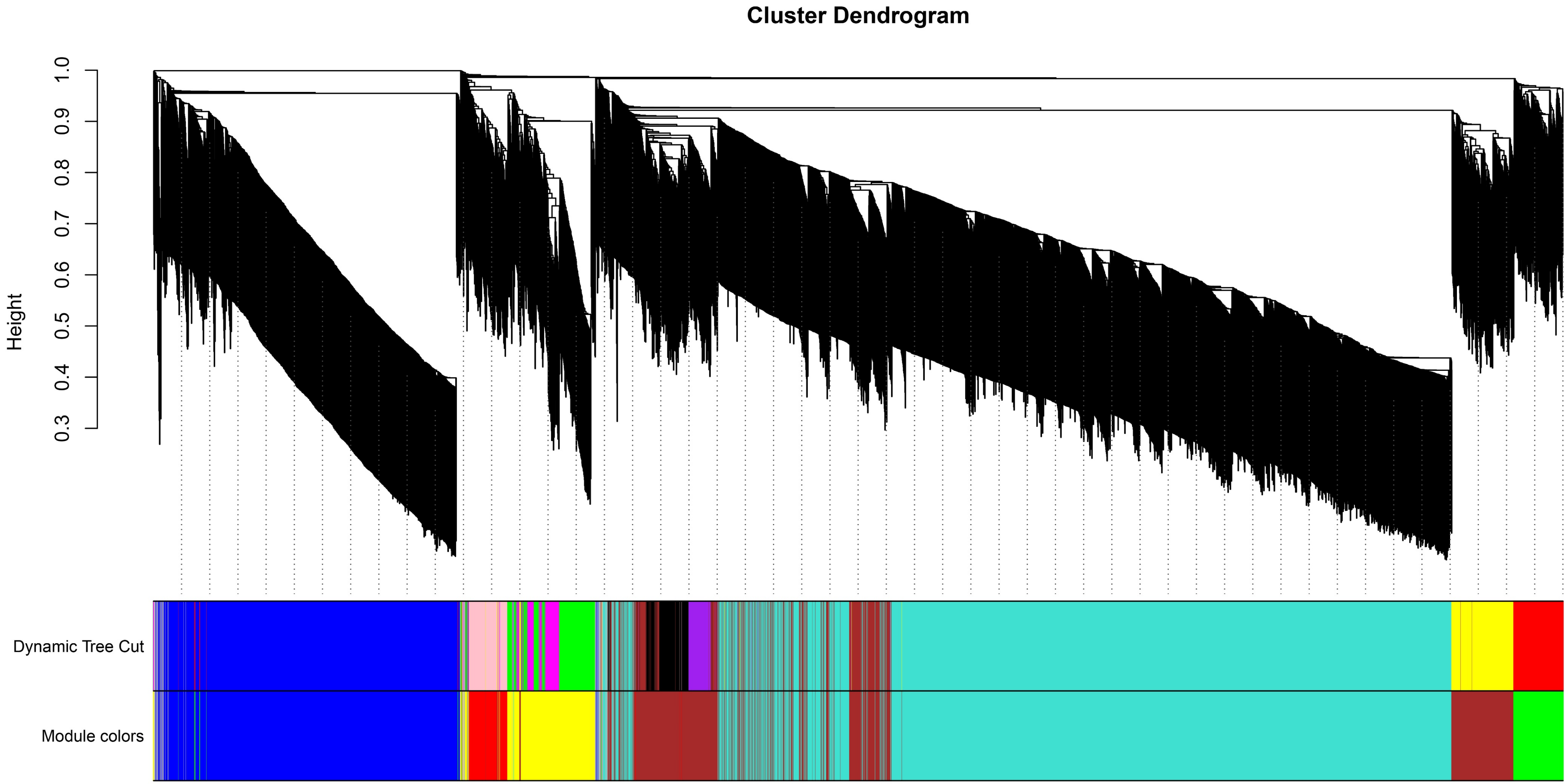
Cluster Dendrogram
Height
1.0
0.9
0.8
0.7
0.6
0.5
0.4
0.3
Dynamic Tree Cut
Module colors

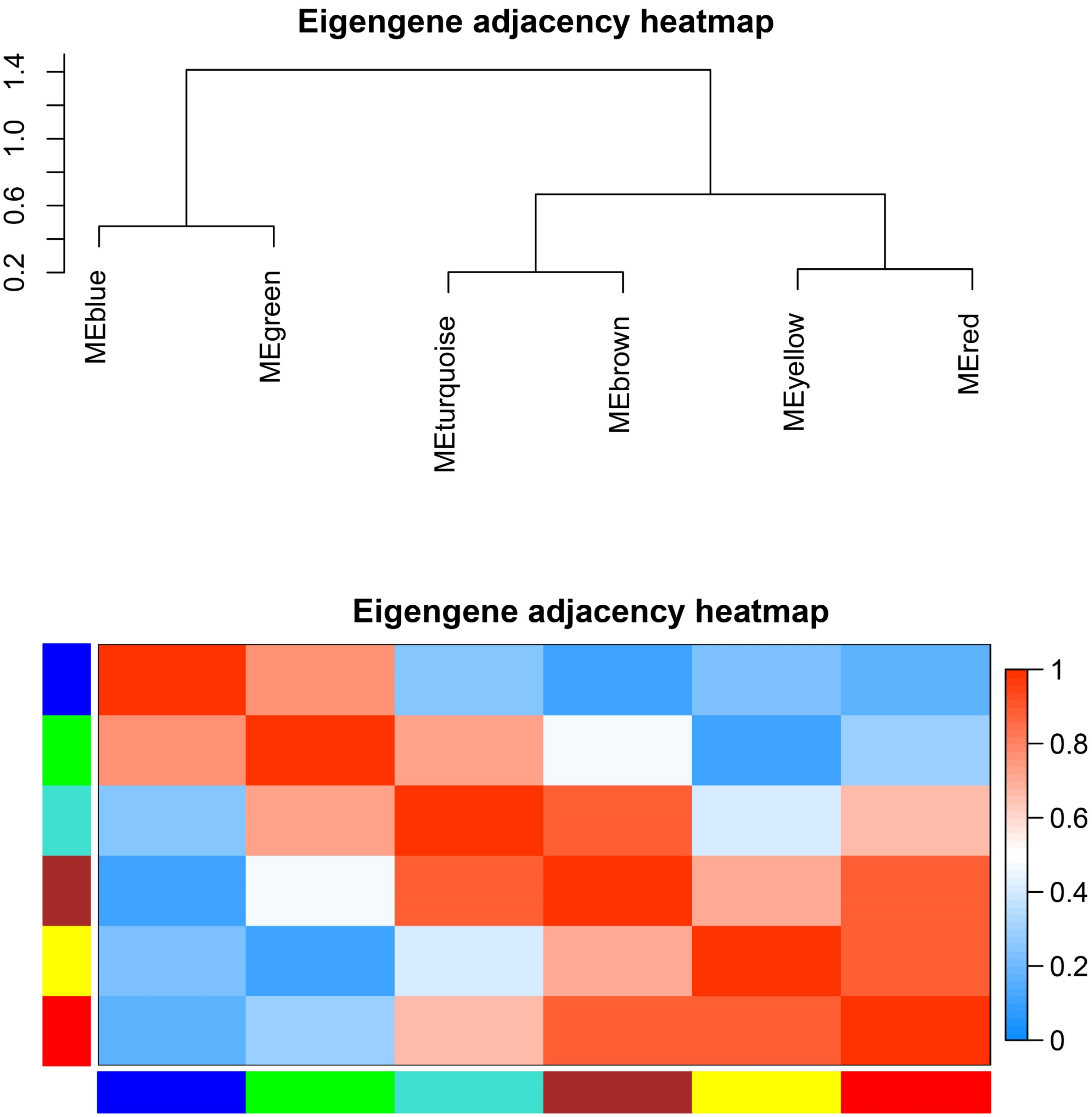
Eigengene adjacency heatmap
0.2
0.6
1.0
1.4
MEblue
MEgreen
MEturquoise
MEbrown
MEyellow
MEred
Eigengene adjacency heatmap
1
0.8
0.6
0.4
0.2
0

Secondary metabolites

G1_Red

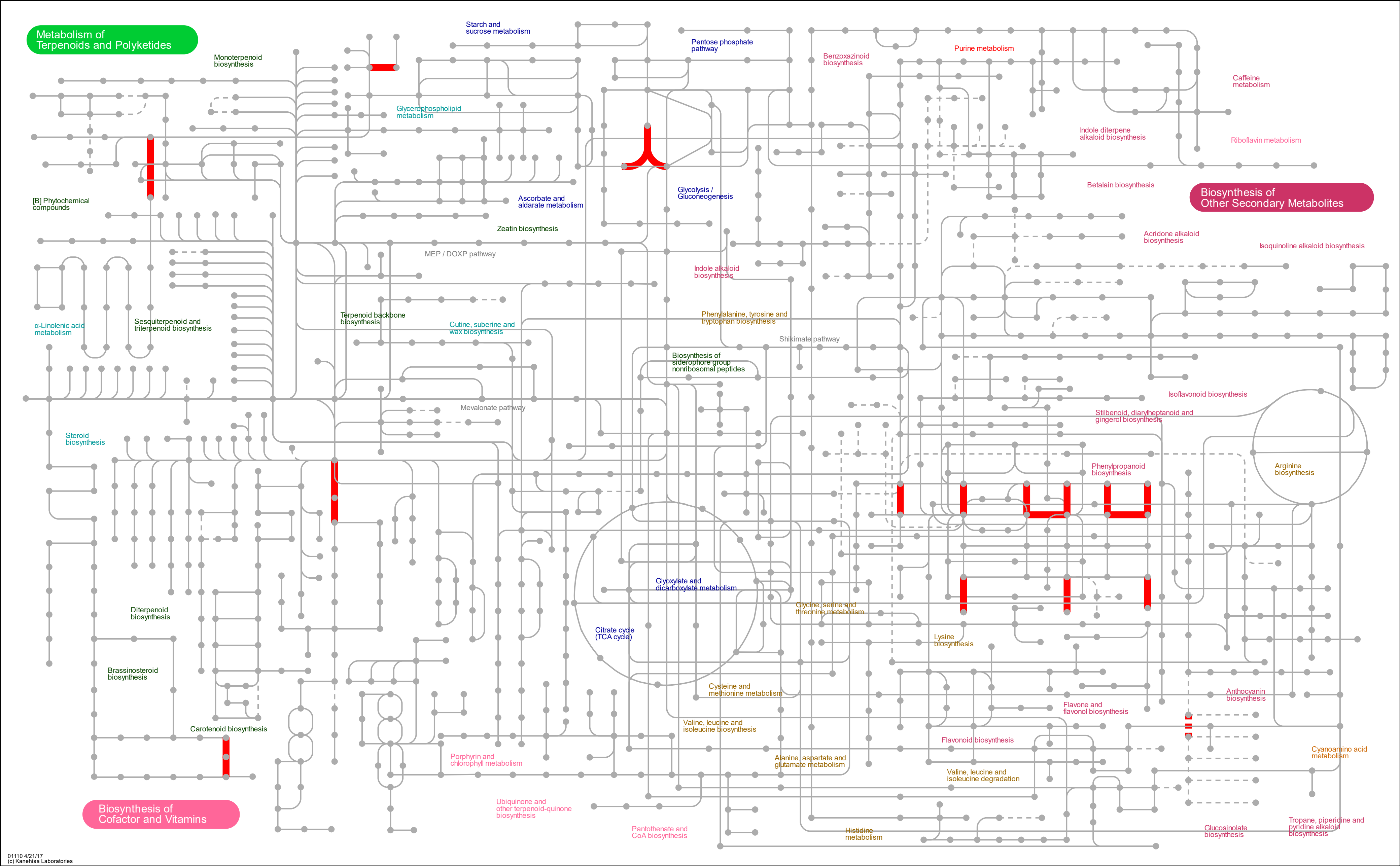

Secondary metabolites

G1_Turquoise

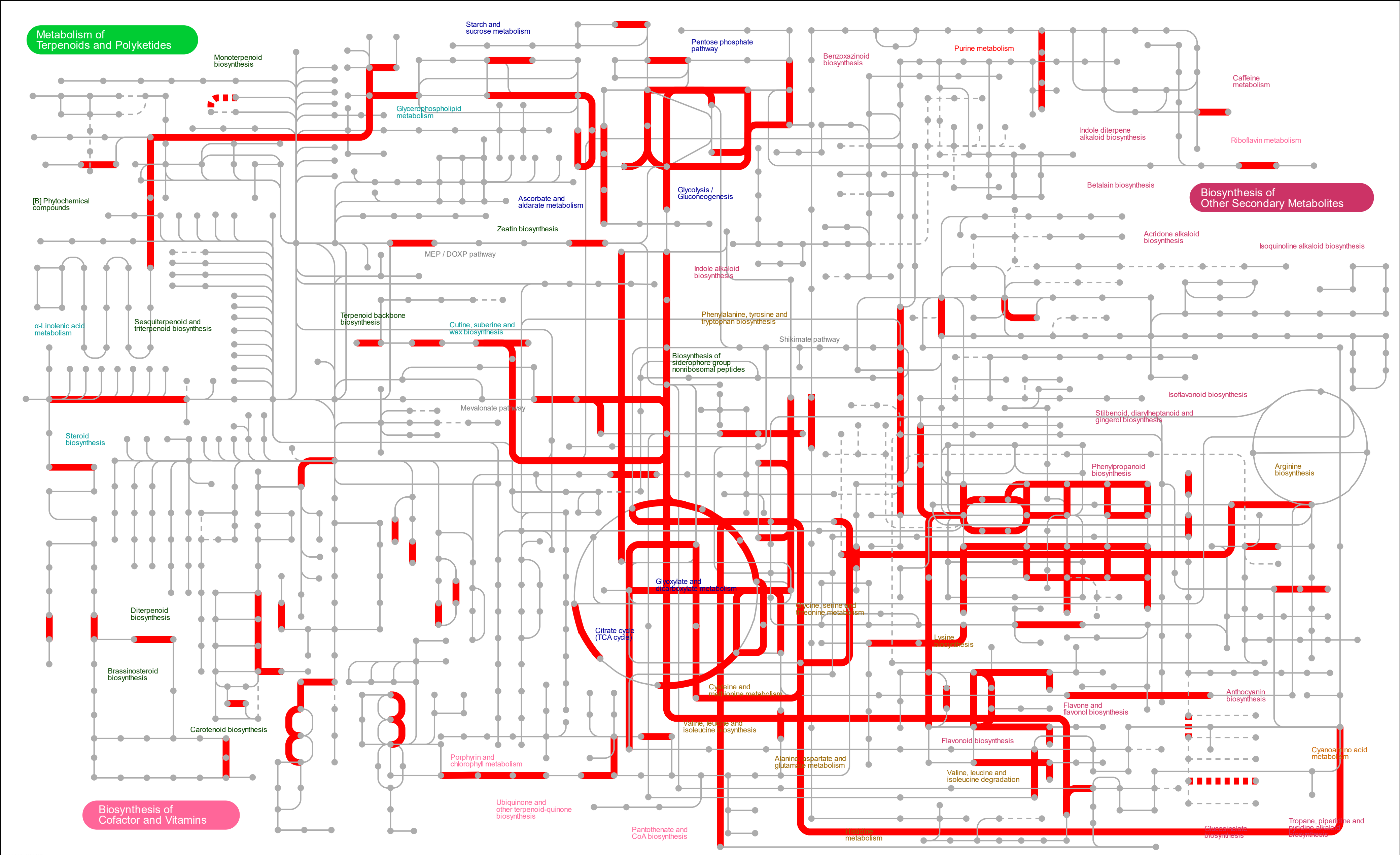

01110 4/21/17
(c) Kanehisa Laboratories

Secondary metabolites

G1_Yellow

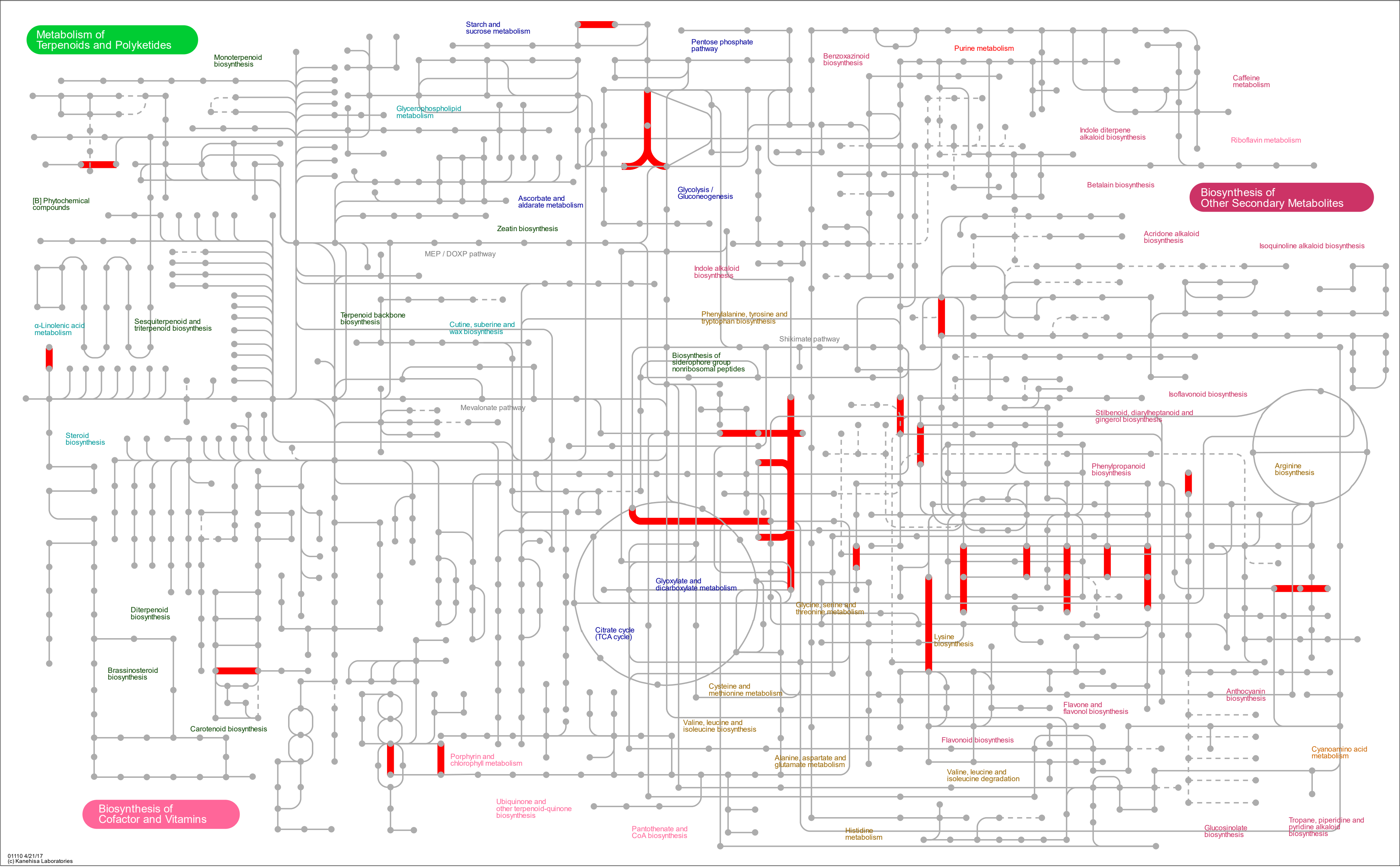

01110 4/21/17
(c) Kanehisa Laboratories

(C) Time Series analysis

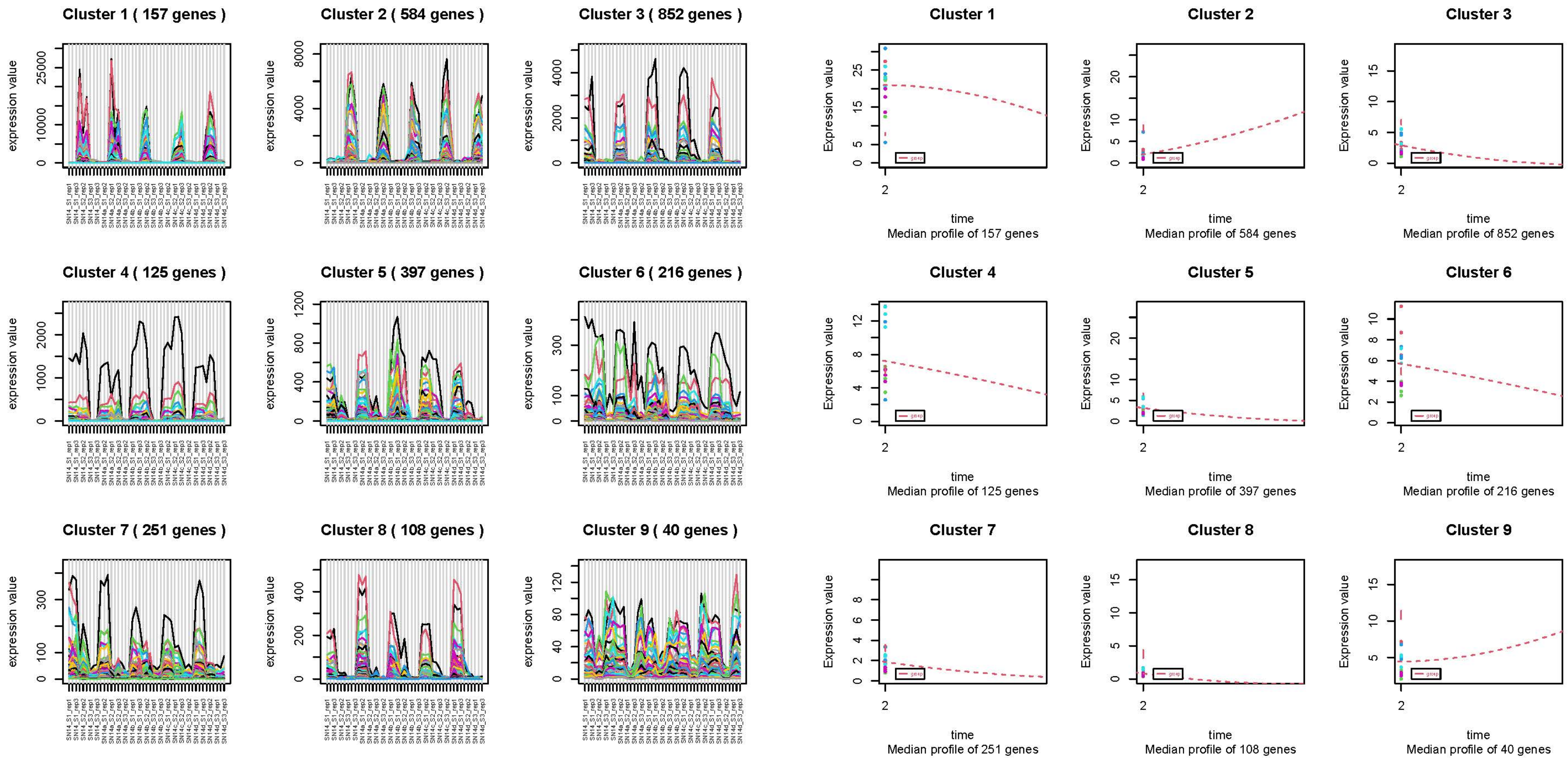

Supplement: Supplementary Figure 7 — Transcriptome analysis of G1 meta-data. [file DataSheet_7.pdf]
