# Supplementary material for: Developmental dynamic transcriptome and systematic analysis reveal the major genes underlying isoflavone accumulation in soybean
Source: Front Plant Sci. 2023 Mar 7;14:1014349. doi: 10.3389/fpls.2023.1014349 (PMC10027745; doi:10.3389/fpls.2023.1014349)

(A) DE analysis

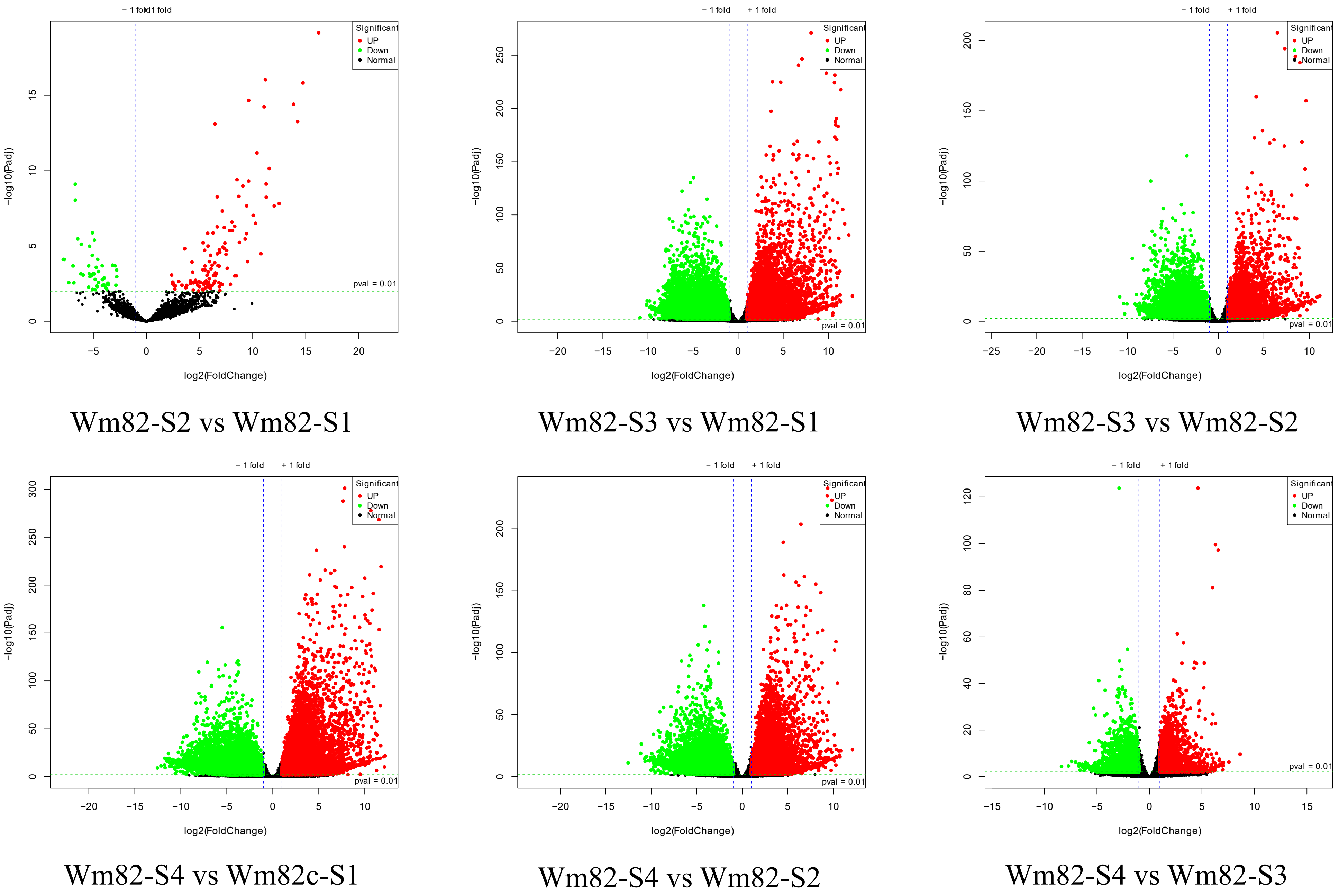

(B) WGCNA

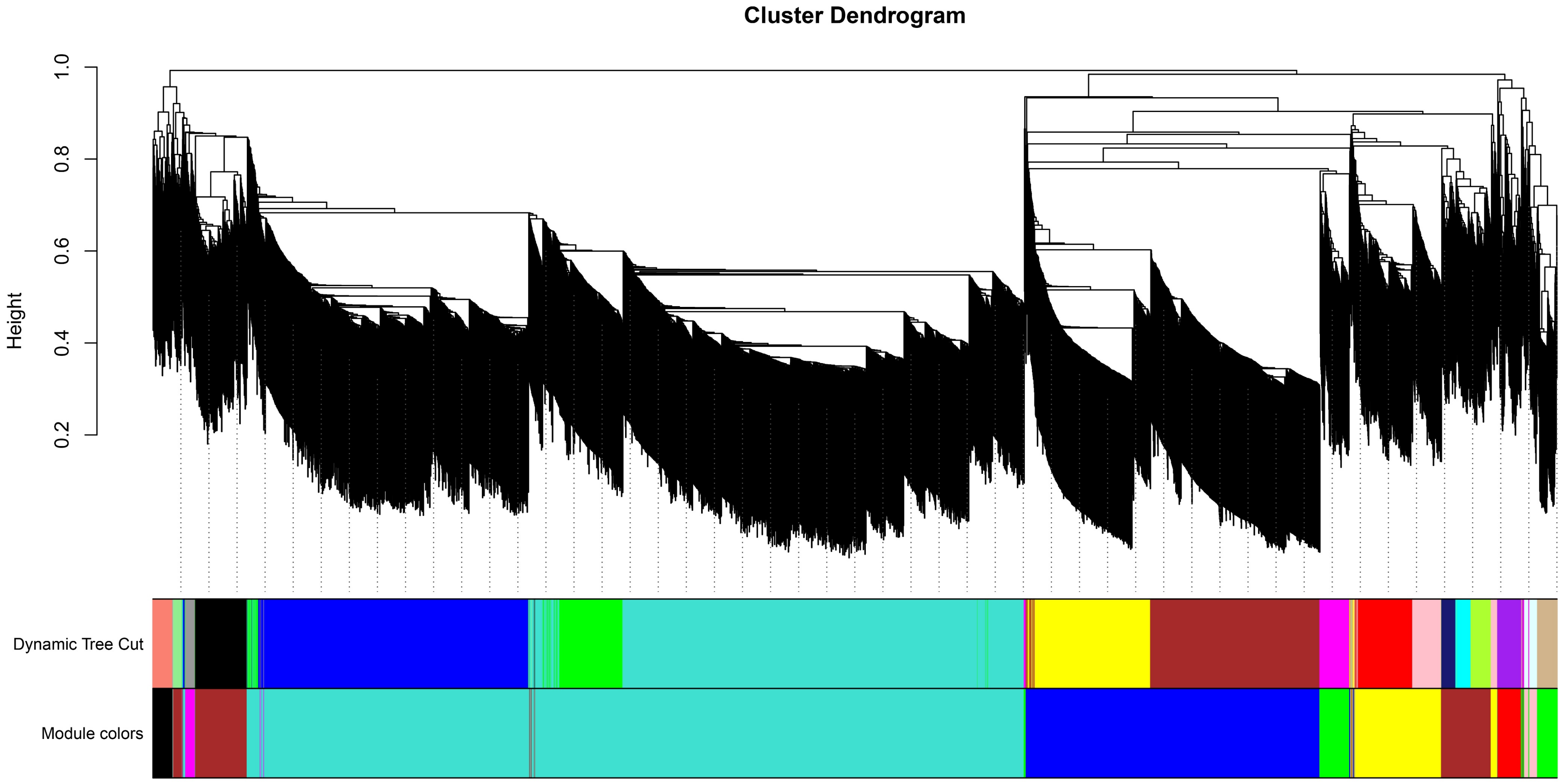
Cluster Dendrogram
Height
1.0
0.8
0.6
0.4
0.2
Dynamic Tree Cut
Module colors

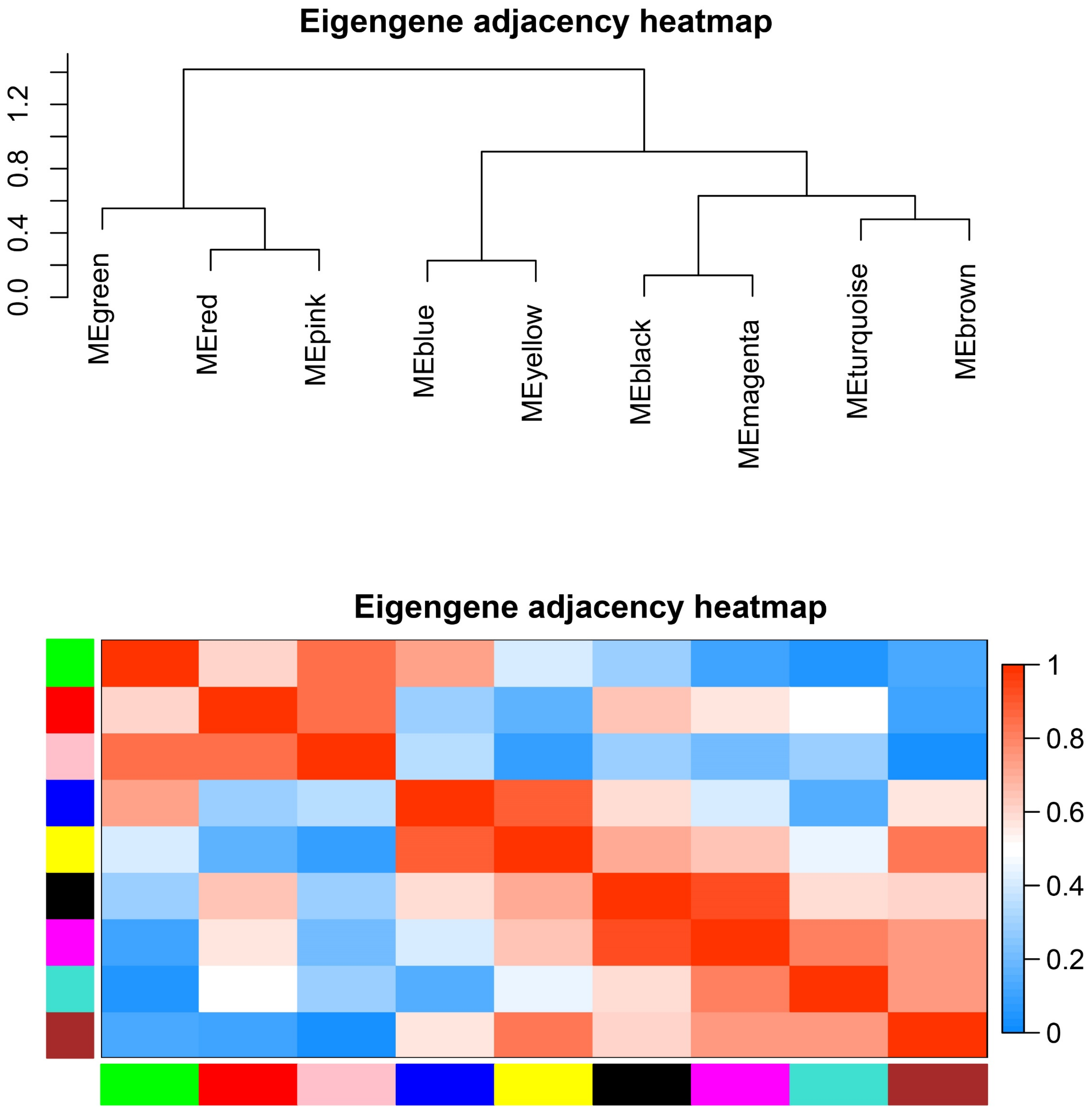
Eigengene adjacency heatmap
0.0
0.4
0.8
1.2
MEgreen
MEred
MEpink
MEblue
MEyellow
MEblack
MEmagenta
MEturquoise
MEbrown
Eigengene adjacency heatmap
0
0.2
0.4
0.6
0.8
1

Secondary metabolites

G2_Blue

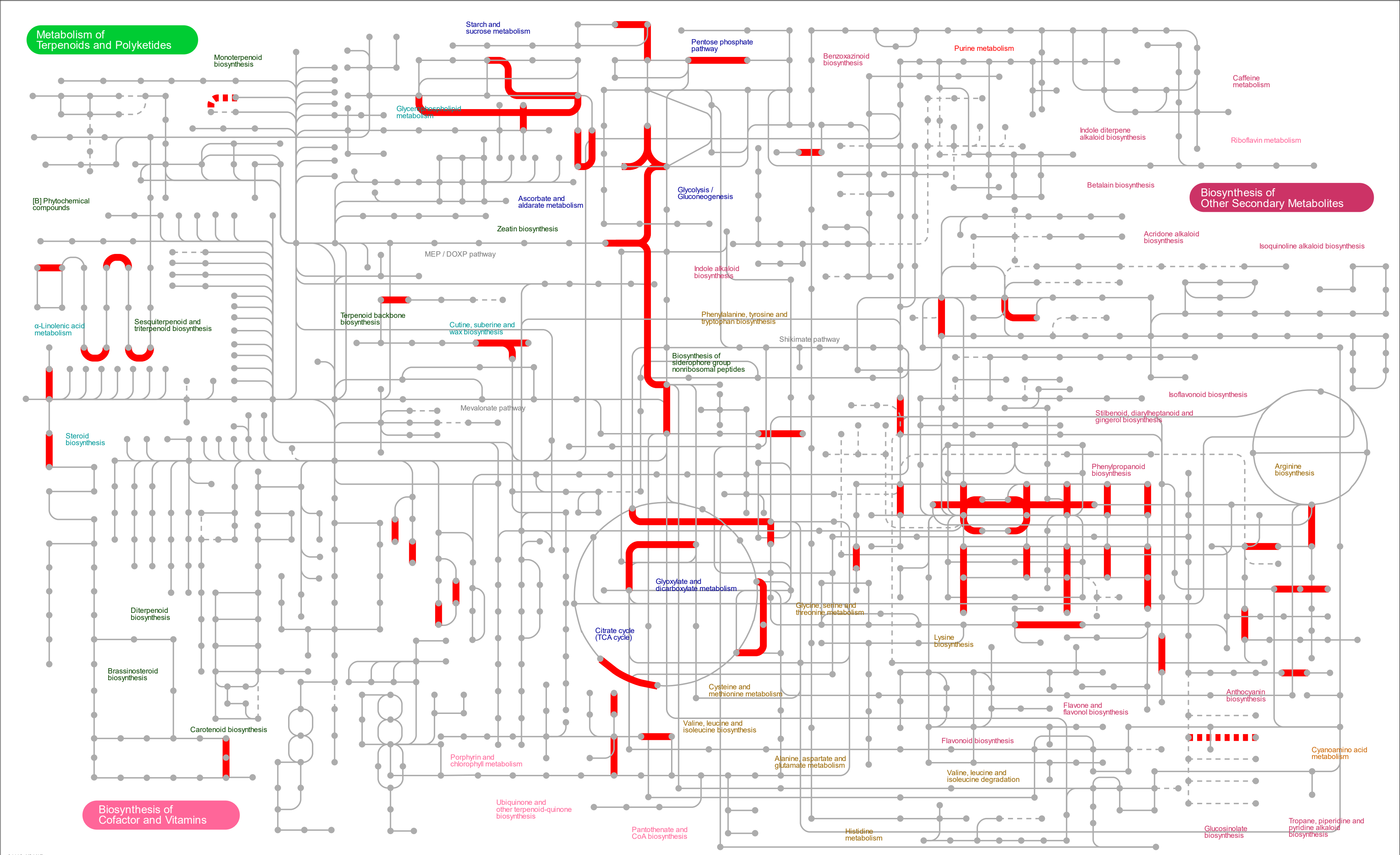

01110 4/21/17

(c) Kanehisa Laboratories

(C) Time Series analysis

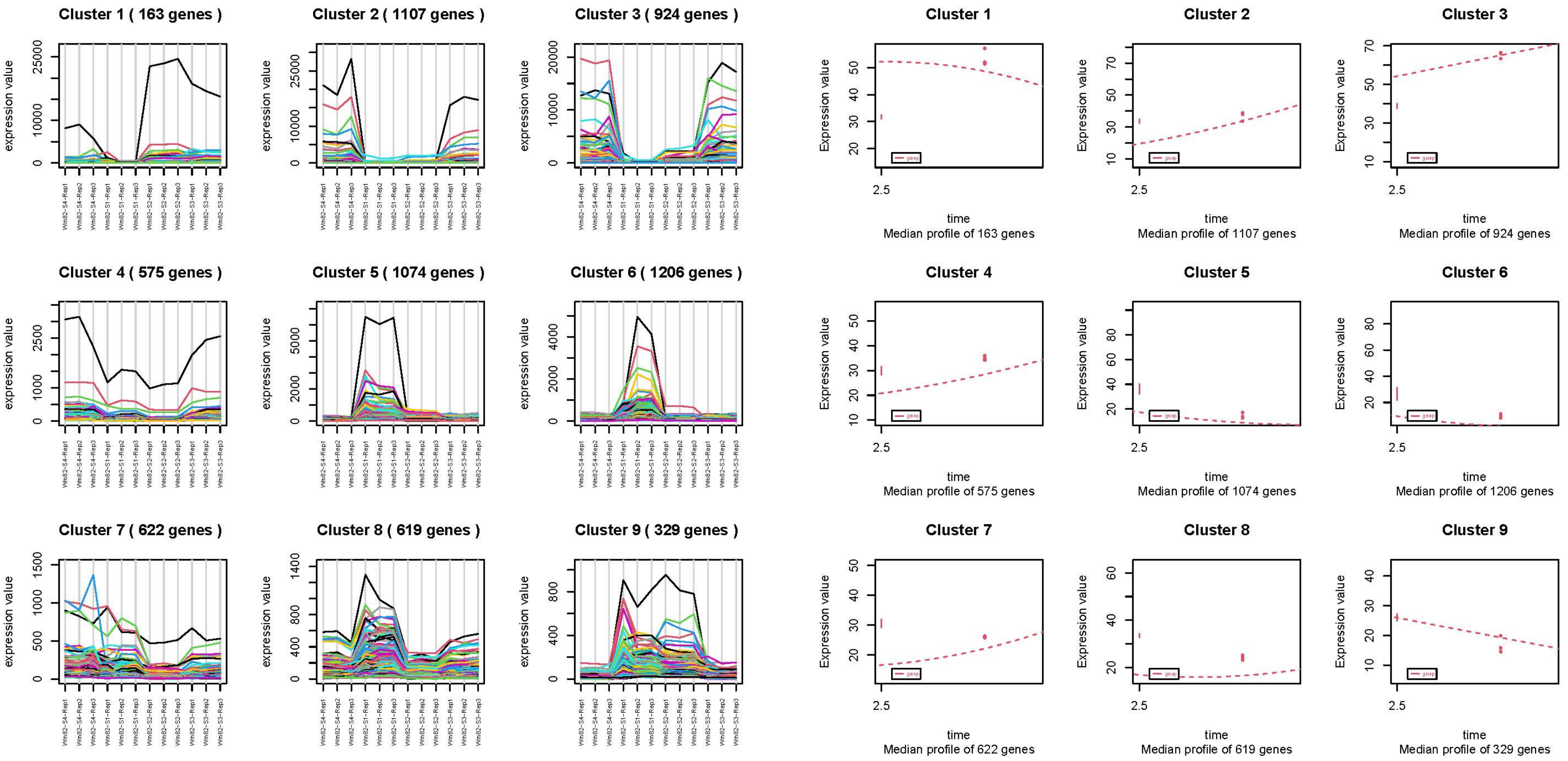

Supplement: Supplementary Figure 8 — Transcriptome analysis of G2 meta-data. [file DataSheet_8.pdf]
