# Supplementary material for: Developmental dynamic transcriptome and systematic analysis reveal the major genes underlying isoflavone accumulation in soybean
Source: Front Plant Sci. 2023 Mar 7;14:1014349. doi: 10.3389/fpls.2023.1014349 (PMC10027745; doi:10.3389/fpls.2023.1014349)

(A) DE analysis

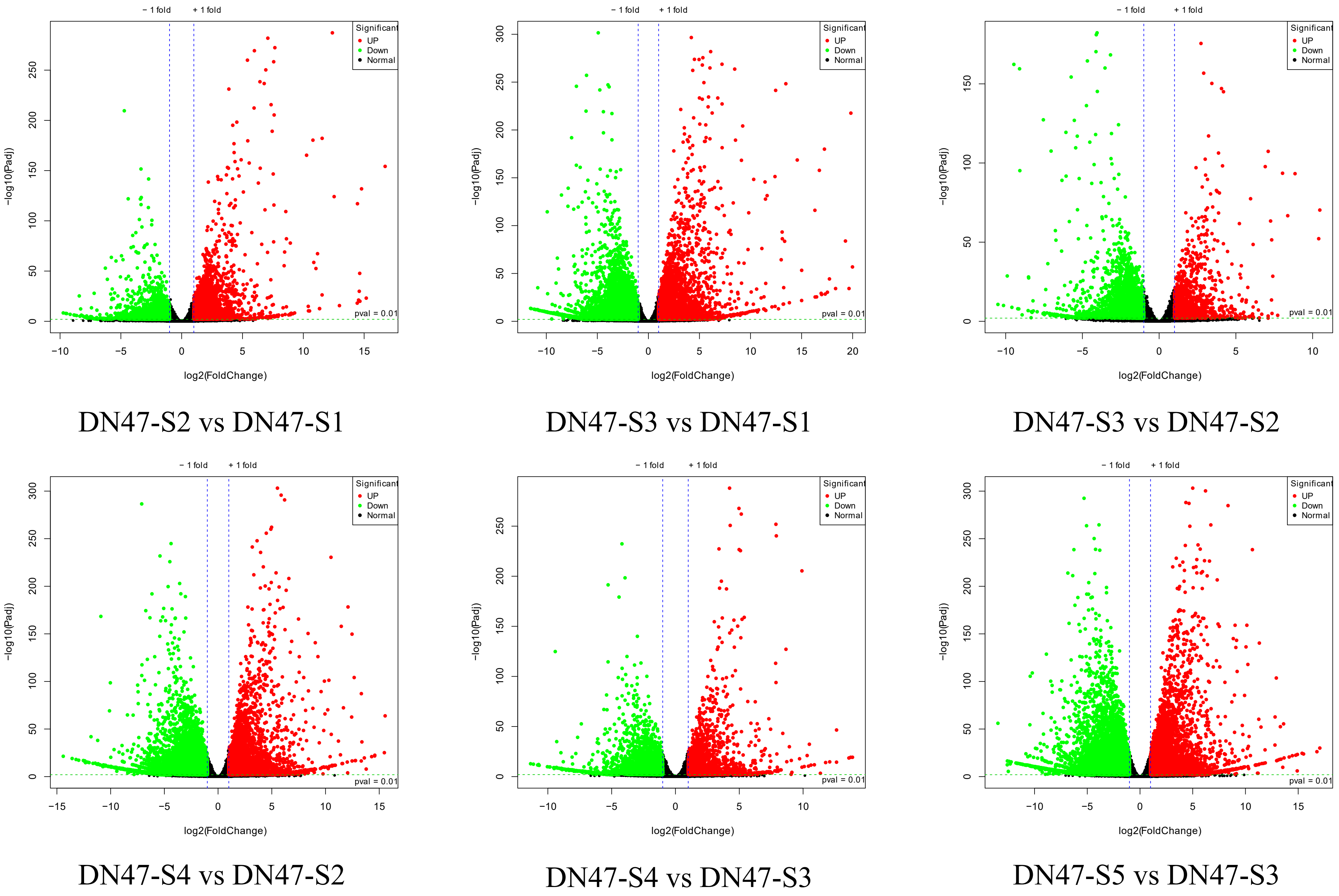

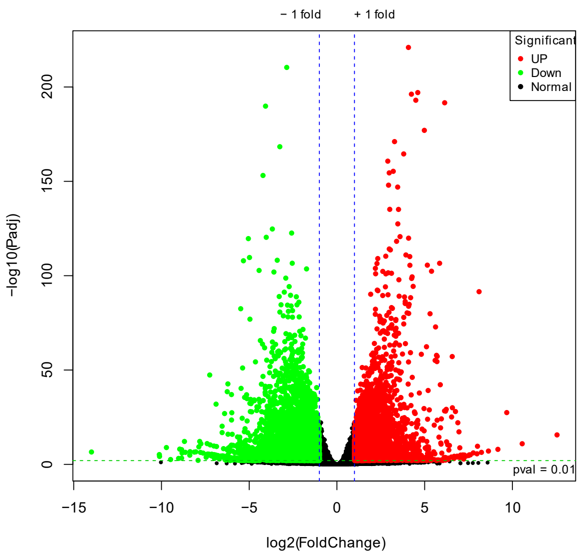

DN47-S5 vs DN47-S4

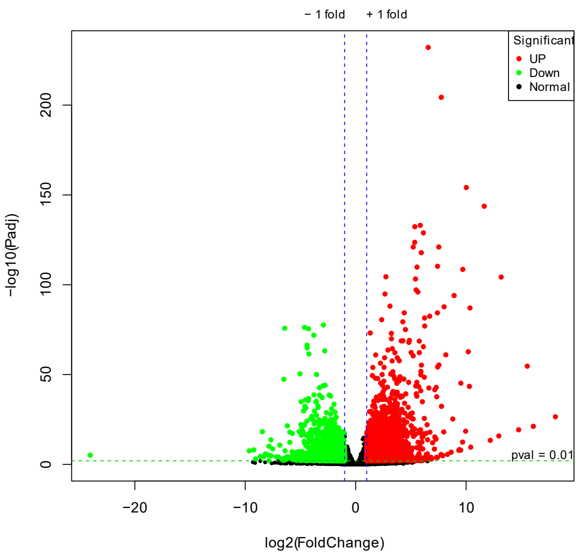

DN47n-S2 vs DN47n-S1

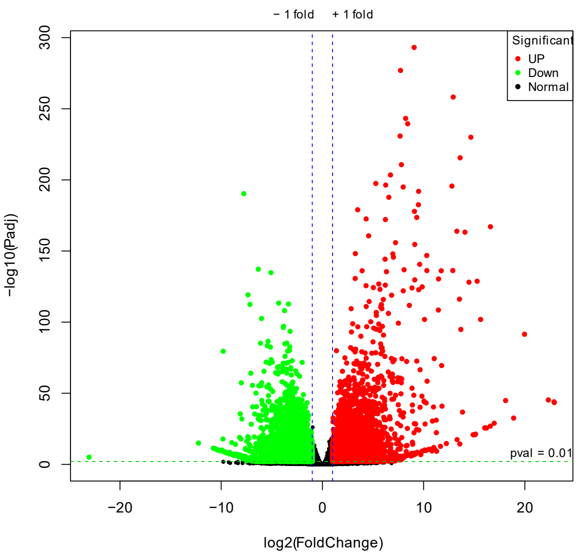

DN47n-S3 vs DN47n-S1

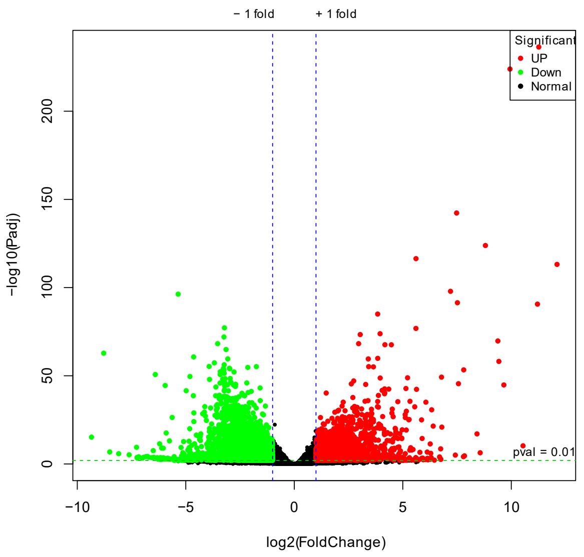

DN47n-S3 vs DN47n-S2

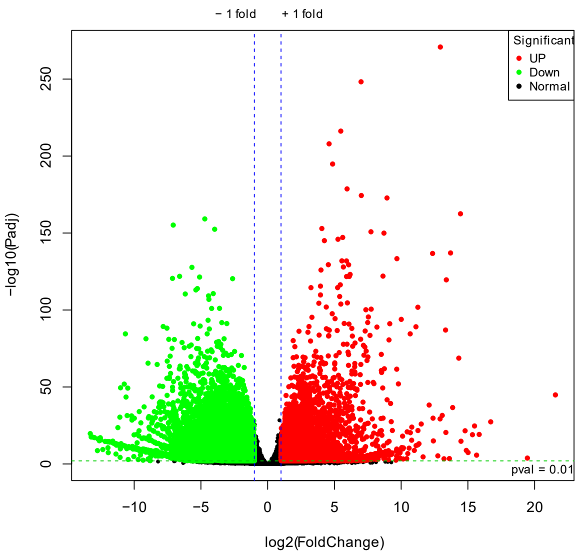

DN47n-S4 vs DN47n-S2

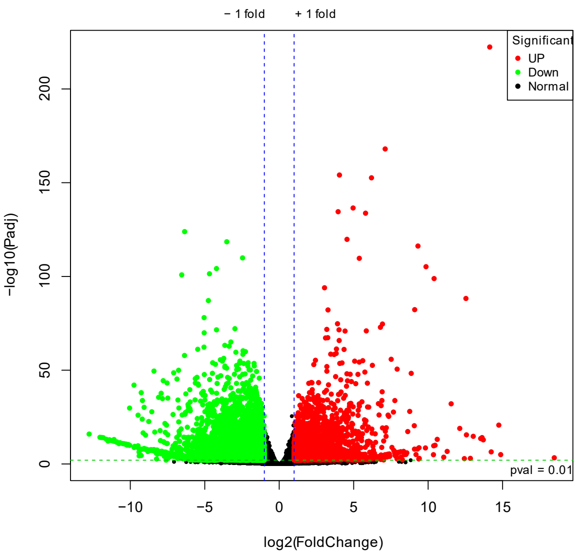

DN47n-S4 vs DN47n-S3

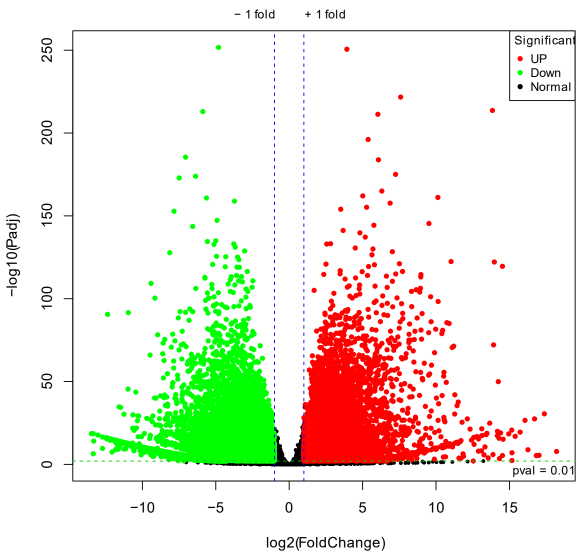

DN47n-S5 vs DN47n-S3

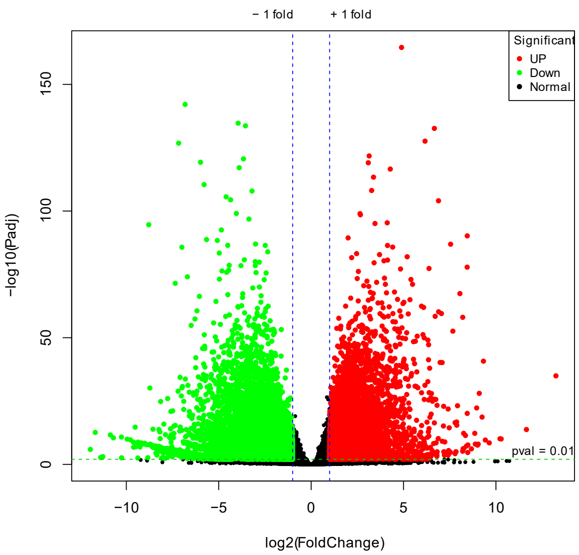

DN47n-S5 vs DN47n-S4

(B) WGCNA

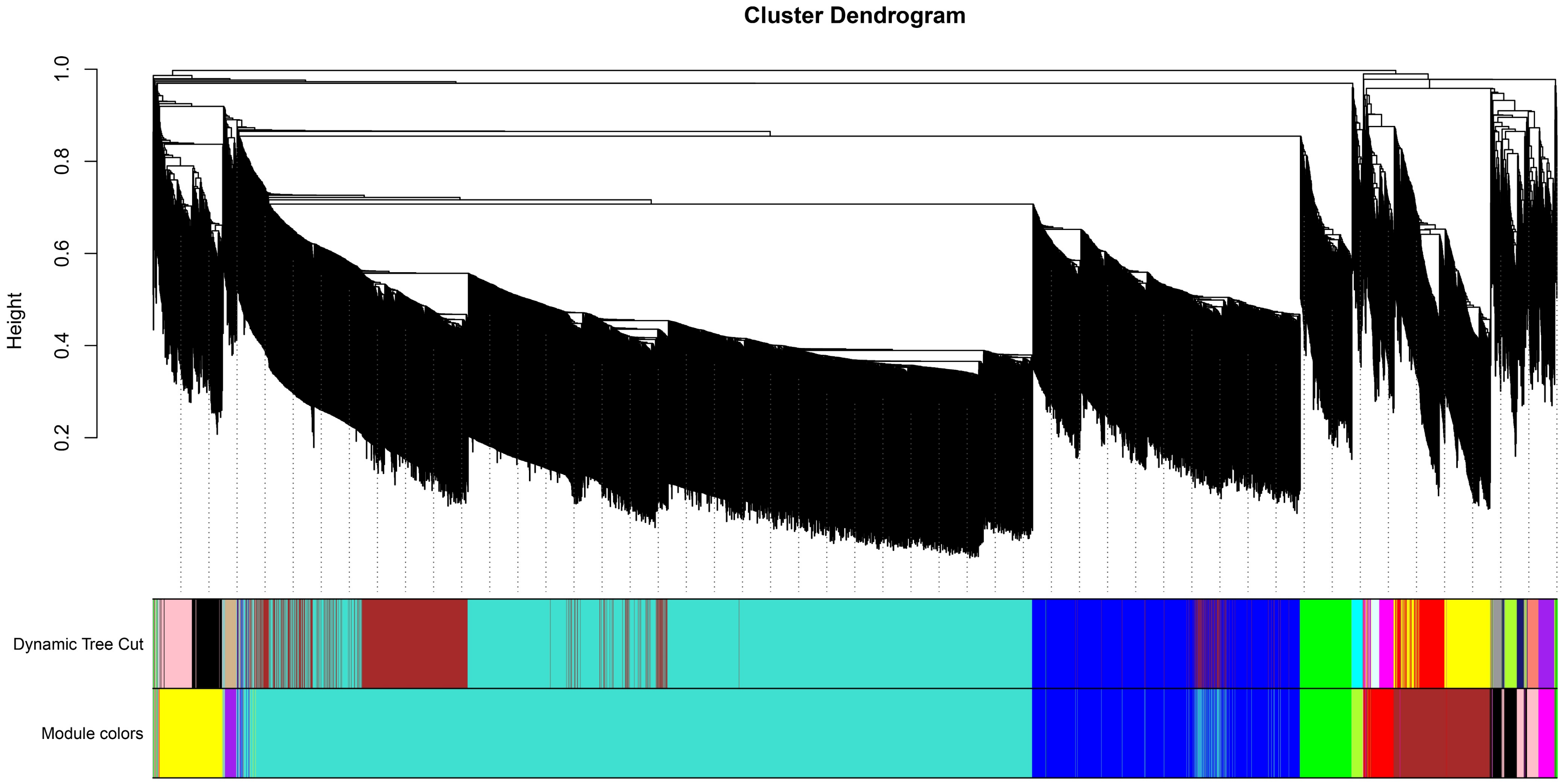
Cluster Dendrogram
Height
1.0
0.8
0.6
0.4
0.2
Dynamic Tree Cut
Module colors

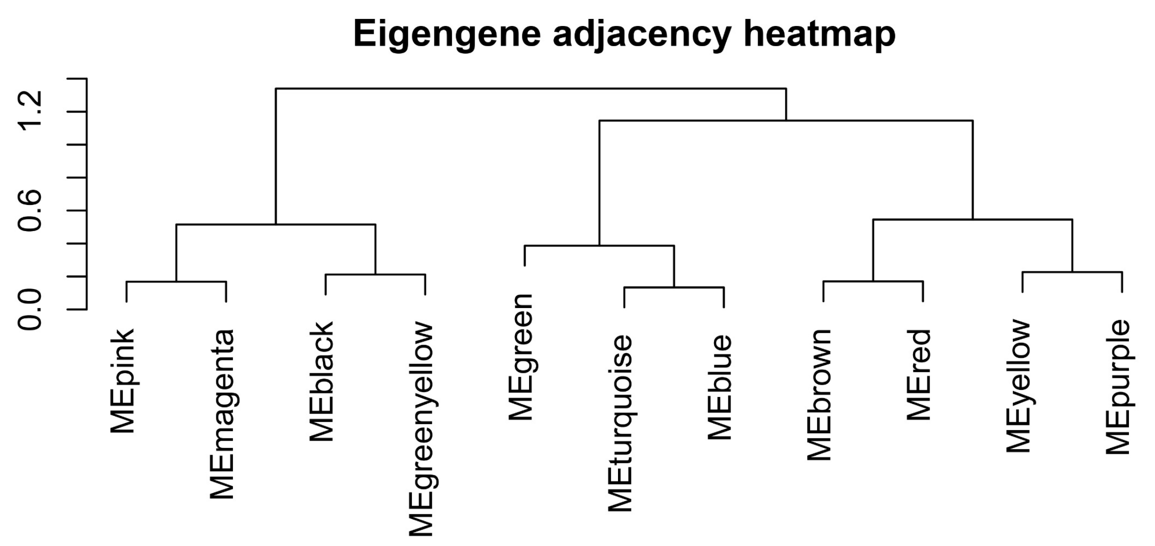

Eigengene adjacency heatmap
0.0
0.6
1.2
MEpink
MEmagenta
MEblack
MEgreenyellow
MEgreen
MEturquoise
MEblue
MEbrown
MEred
MEyellow
MEpurple

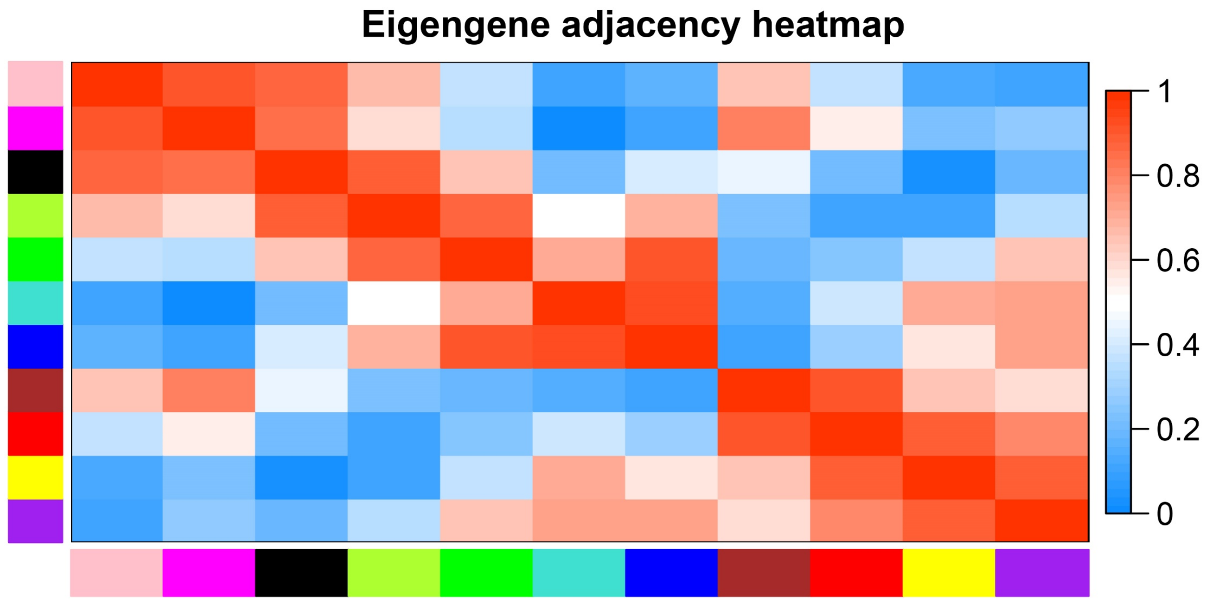

Eigengene adjacency heatmap
1
0.8
0.6
0.4
0.2
0

Secondary metabolites

G3_Blue

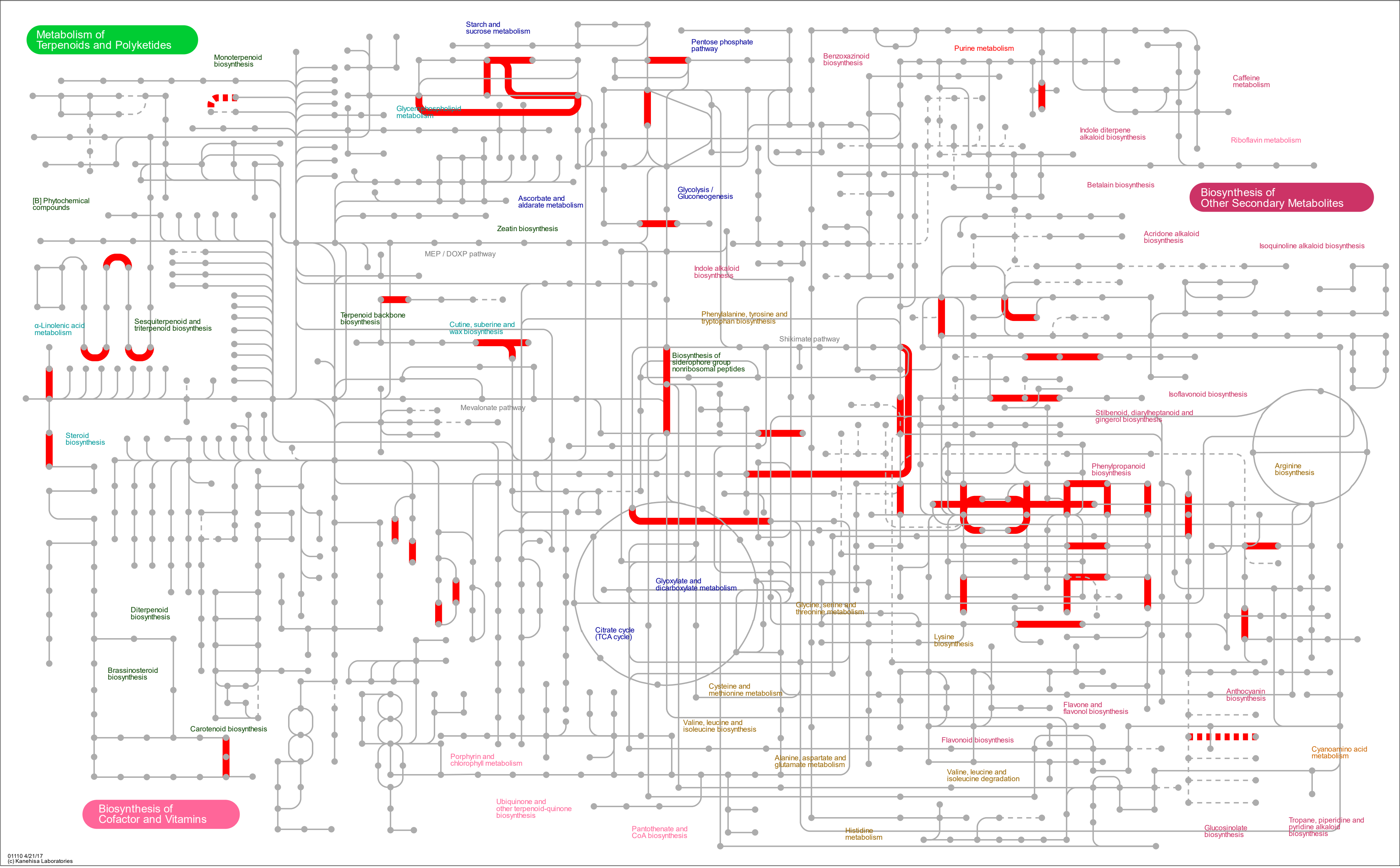

Secondary metabolites

G3_black

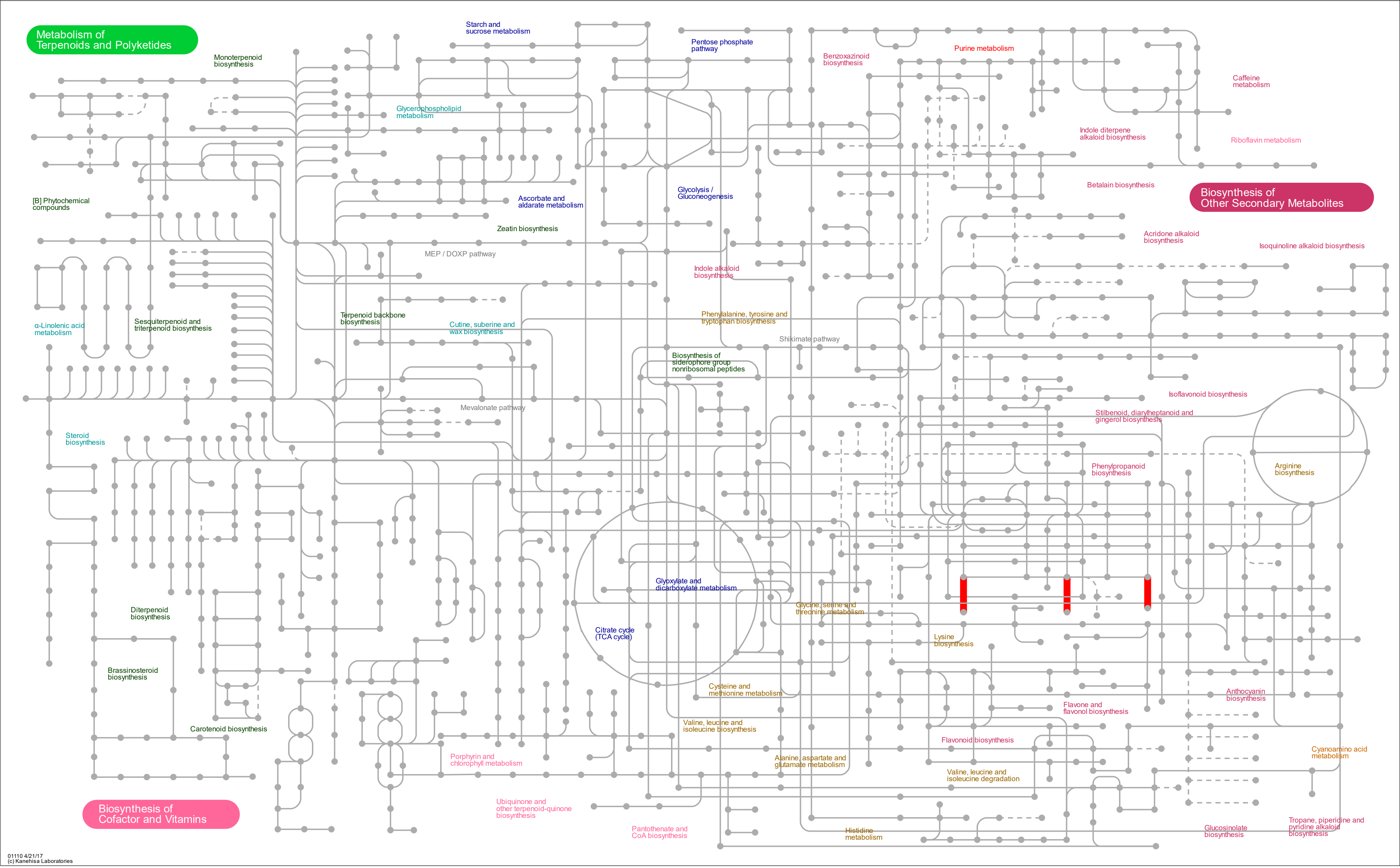

(C) Time Series analysis

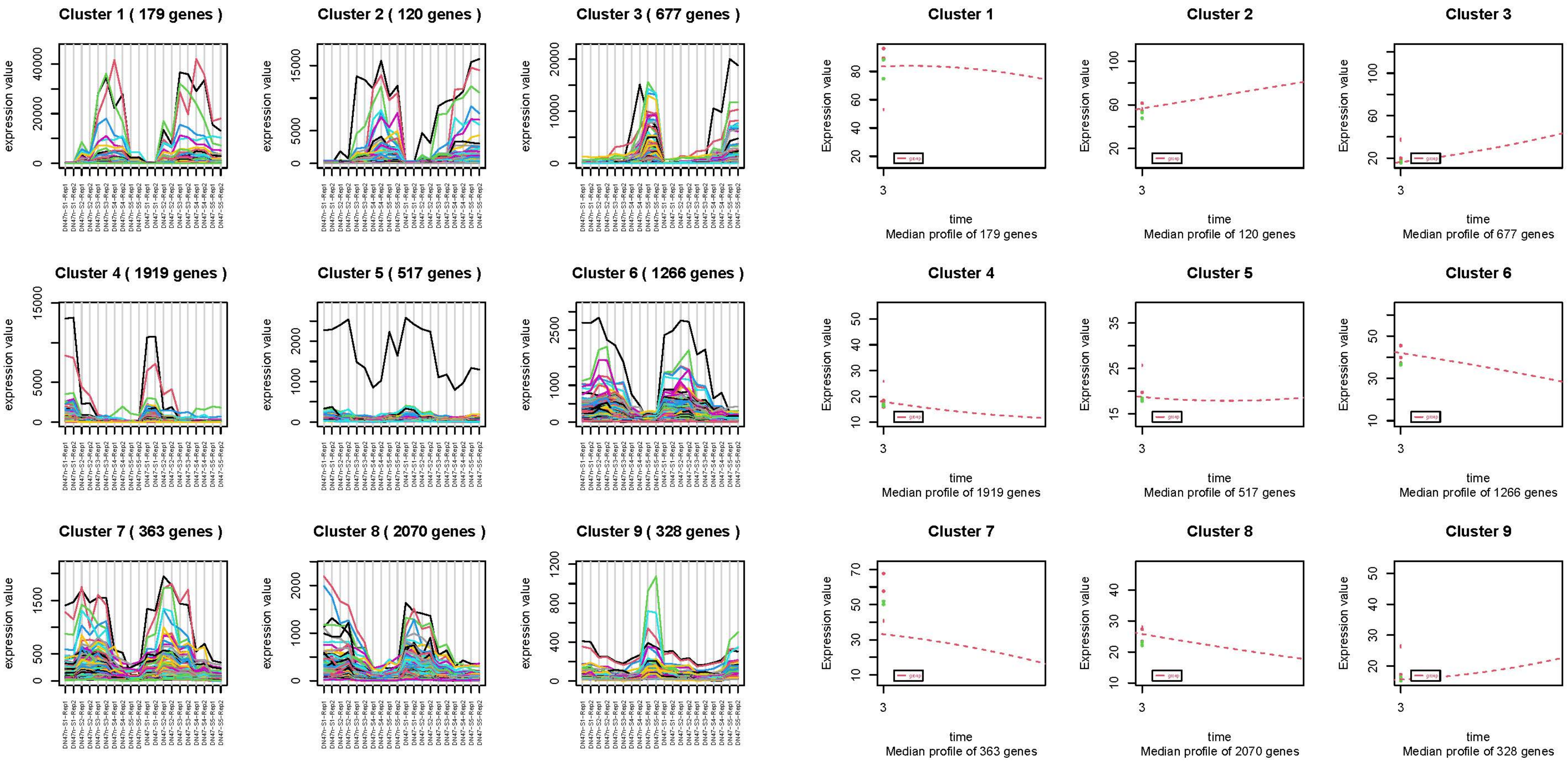

Supplement: Supplementary Figure 9 — Transcriptome analysis of G3 meta-data. [file DataSheet_9.pdf]
