# Supplementary material for: Developmental dynamic transcriptome and systematic analysis reveal the major genes underlying isoflavone accumulation in soybean
Source: Front Plant Sci. 2023 Mar 7;14:1014349. doi: 10.3389/fpls.2023.1014349 (PMC10027745; doi:10.3389/fpls.2023.1014349)

(A) DE analysis

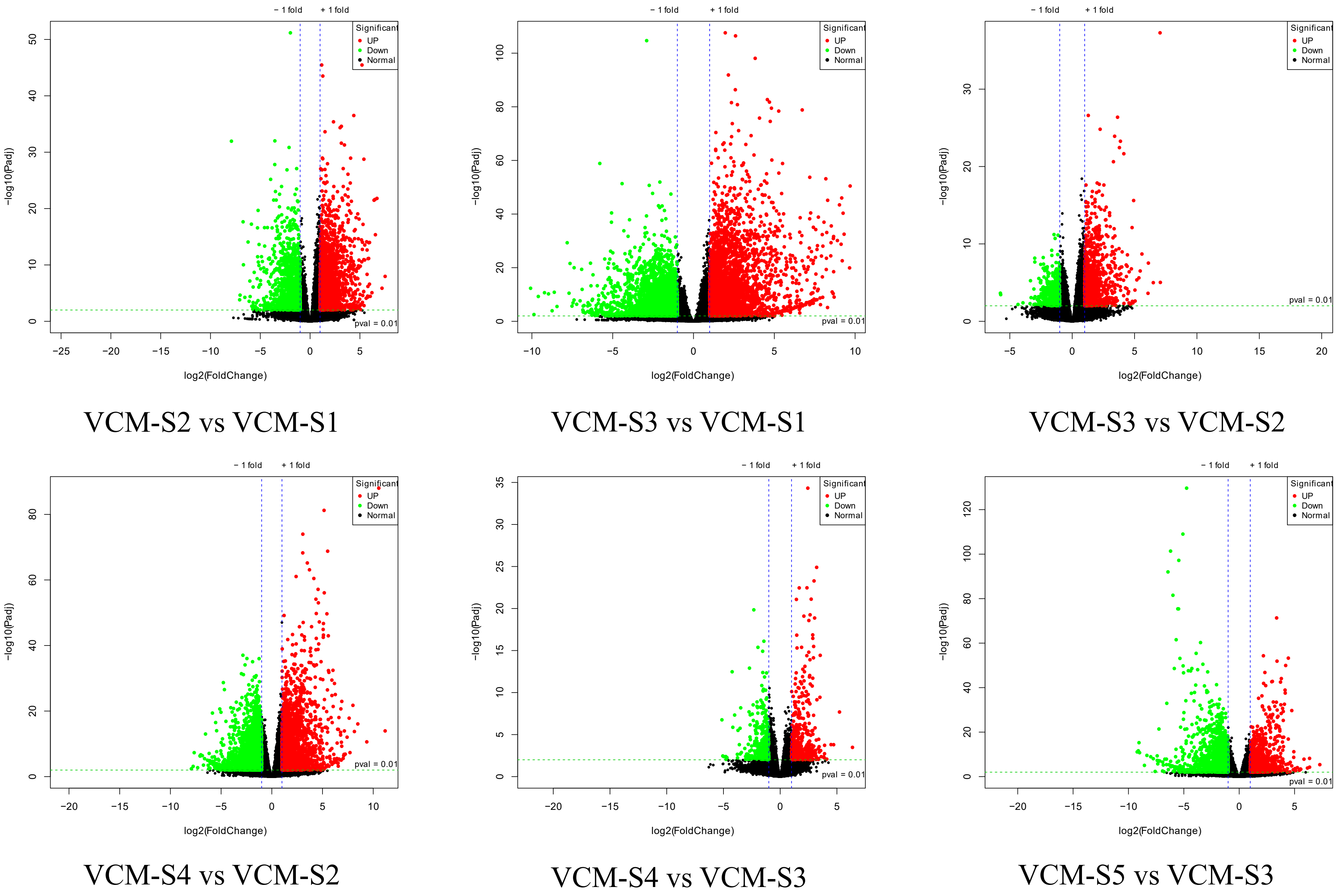

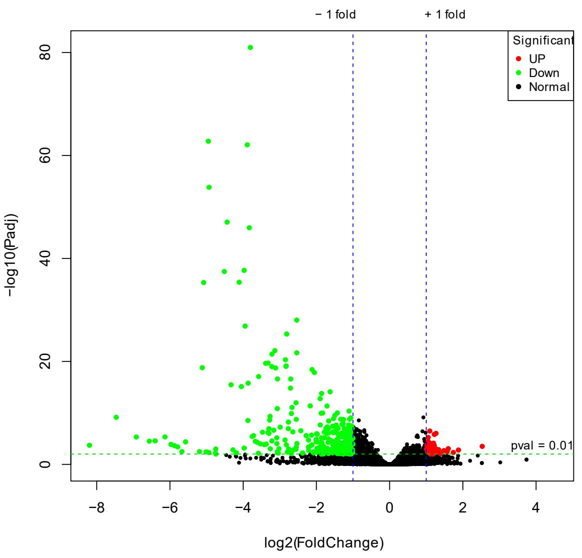

VCM-S5 VS VCM-S4

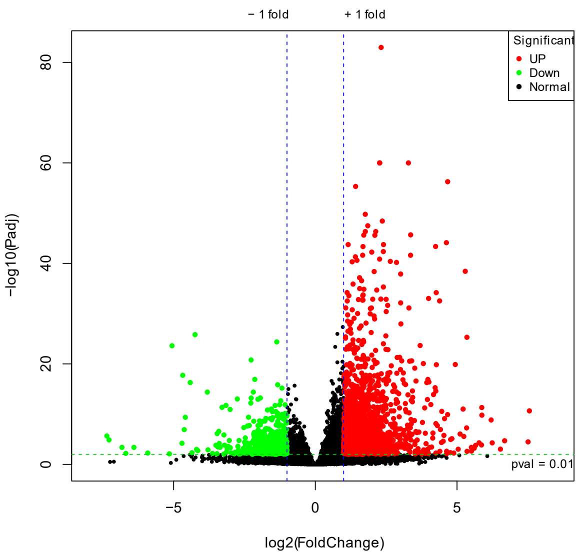

vcm-S2 vs vcm-S1

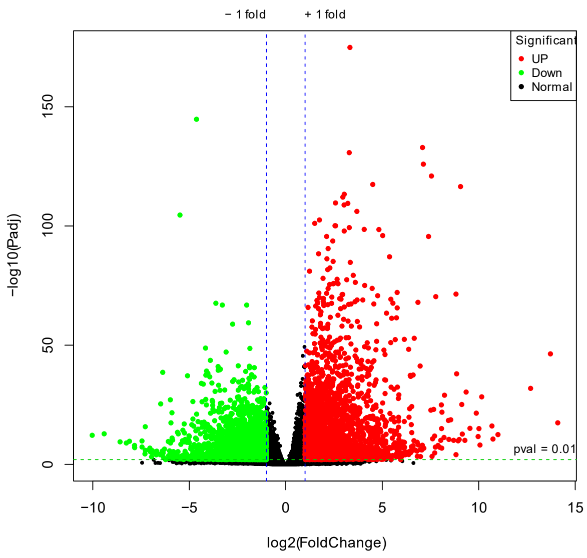

vcm-S3 vs vcm-S1

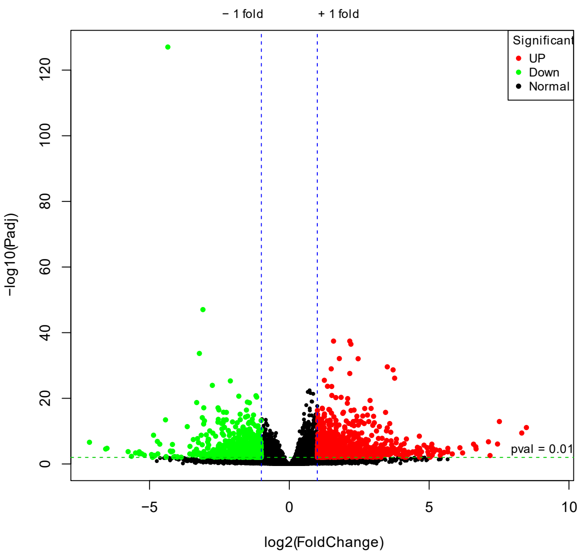

vcm-S3 vs vcm-S2

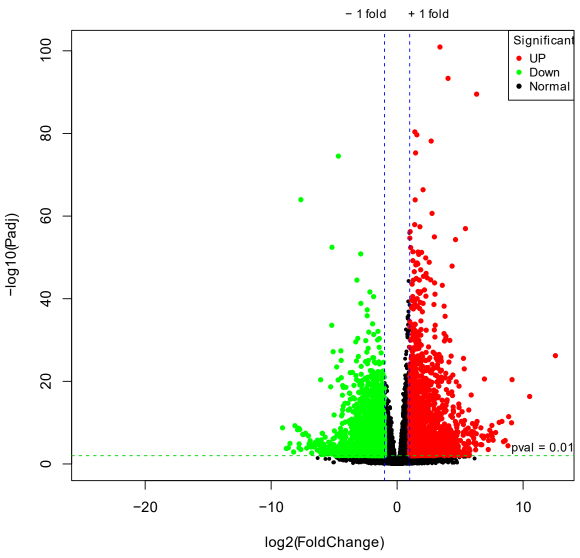

vcm-S4 vs vcm-S2

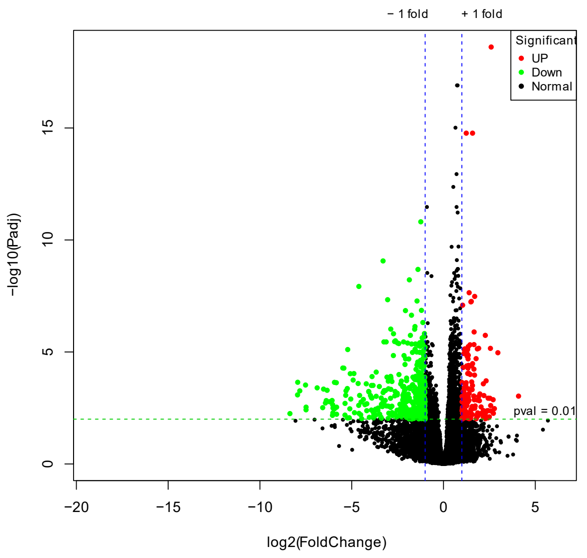

vcm-S4 vs vcm-S3

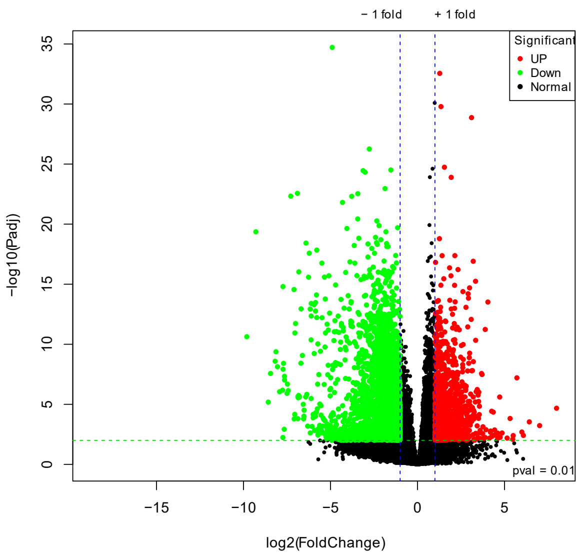

vcm-S5 vs vcm-S3

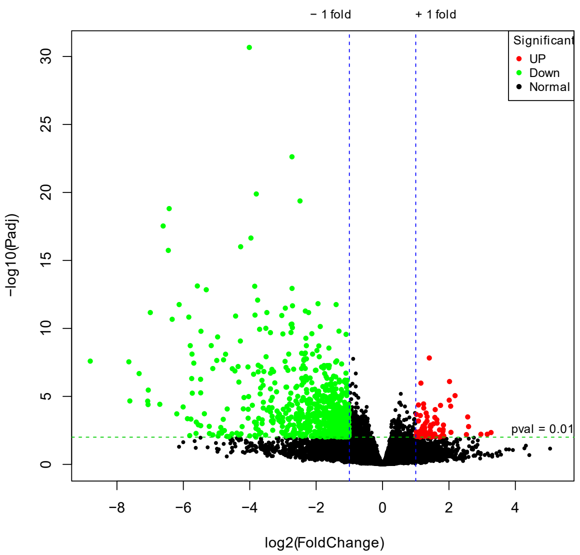

vcm-S5 vs vcm-S4

(B) WGCNA of VCM

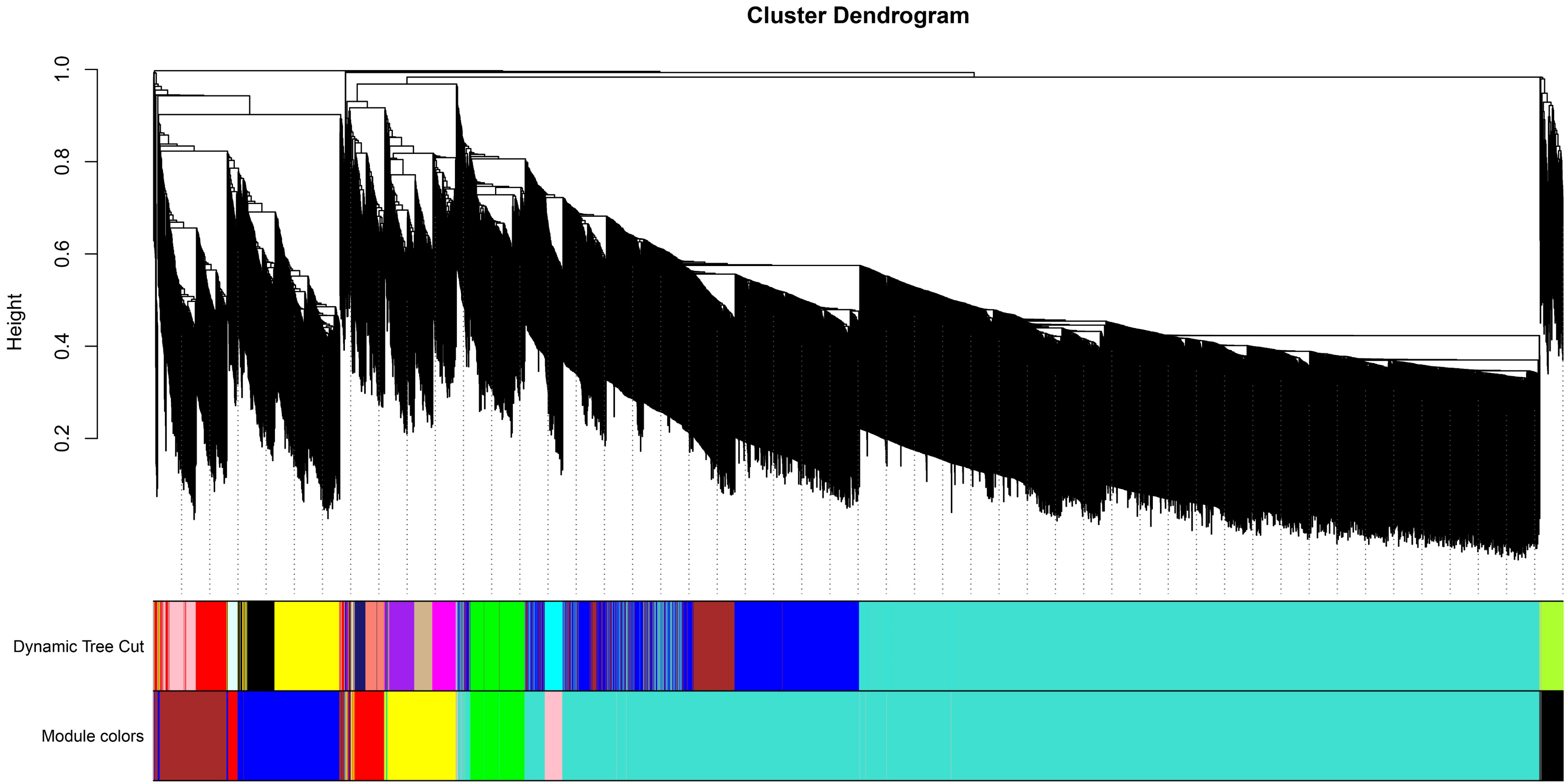

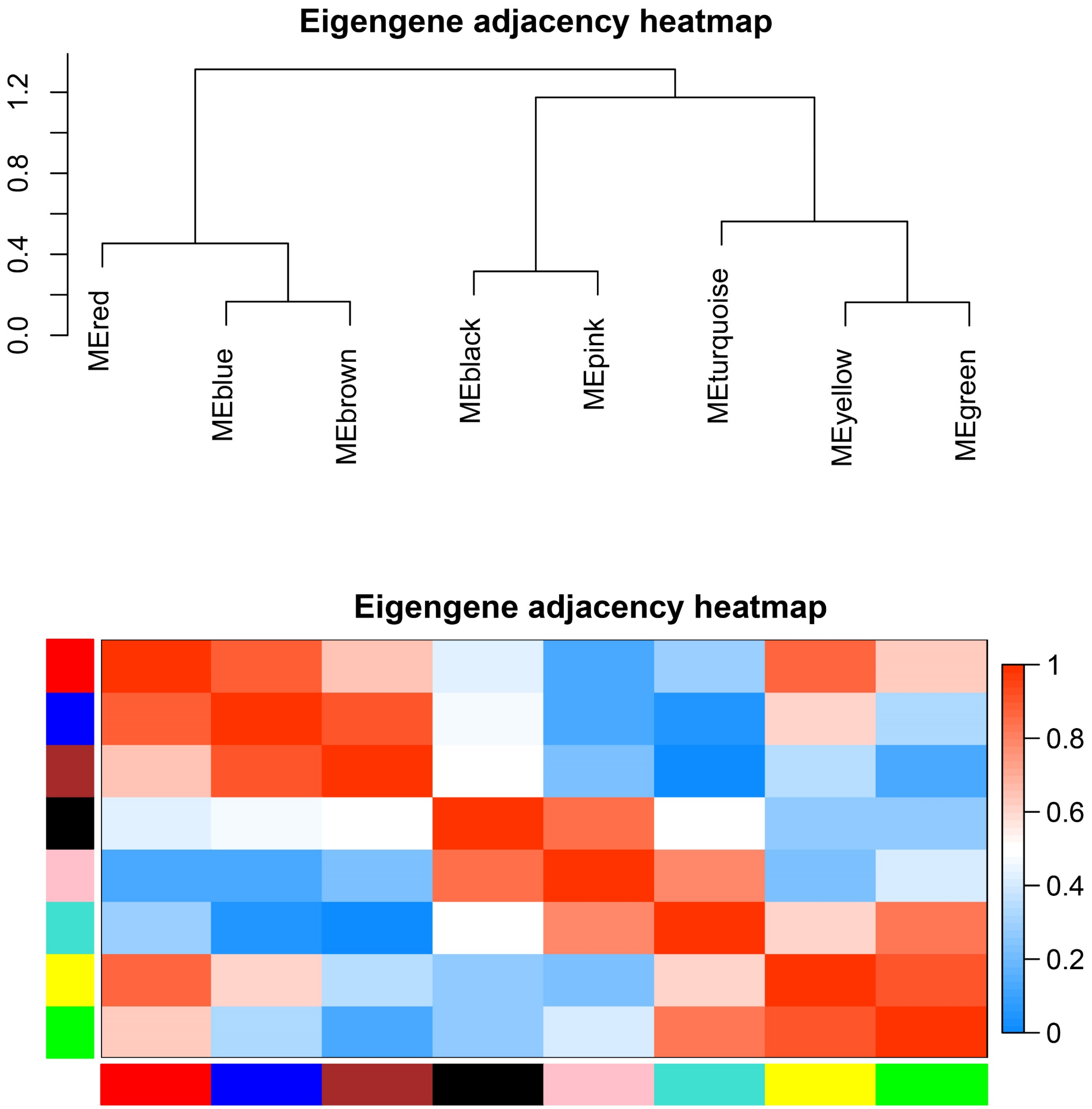

Eigengene adjacency heatmap
0.0
0.4
0.8
1.2
MEred
MEblue
MEbrown
MEblack
MEpink
MEturquoise
MEyellow
MEgreen
Eigengene adjacency heatmap
1
0.8
0.6
0.4
0.2
0

Secondary metabolites

G4_VCM_Turquoise

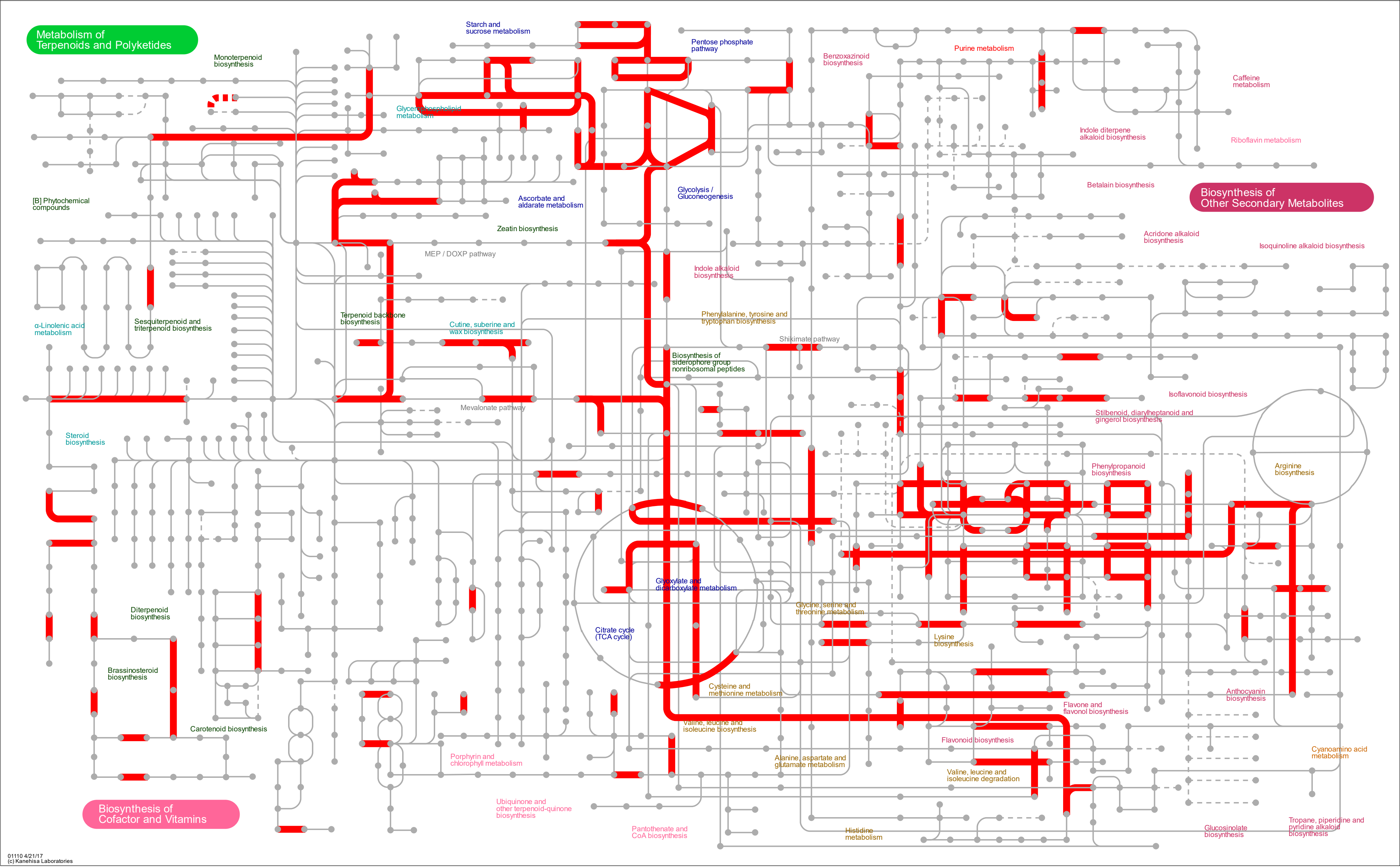

Secondary metabolites

G4_VCM_Green

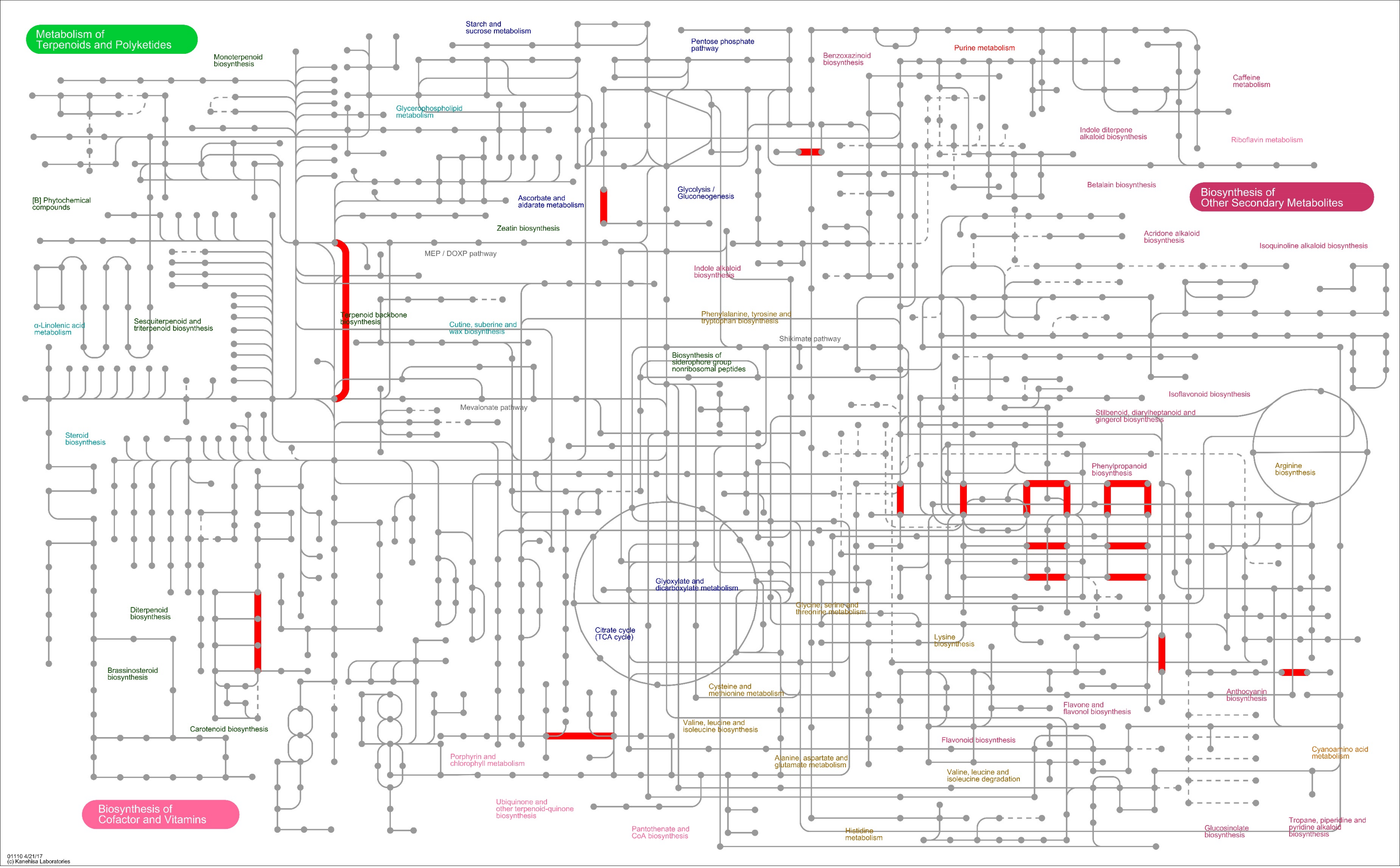

01110 4/21/17
(c) Kanehisa Laboratories

(C) WGCNA of VCm

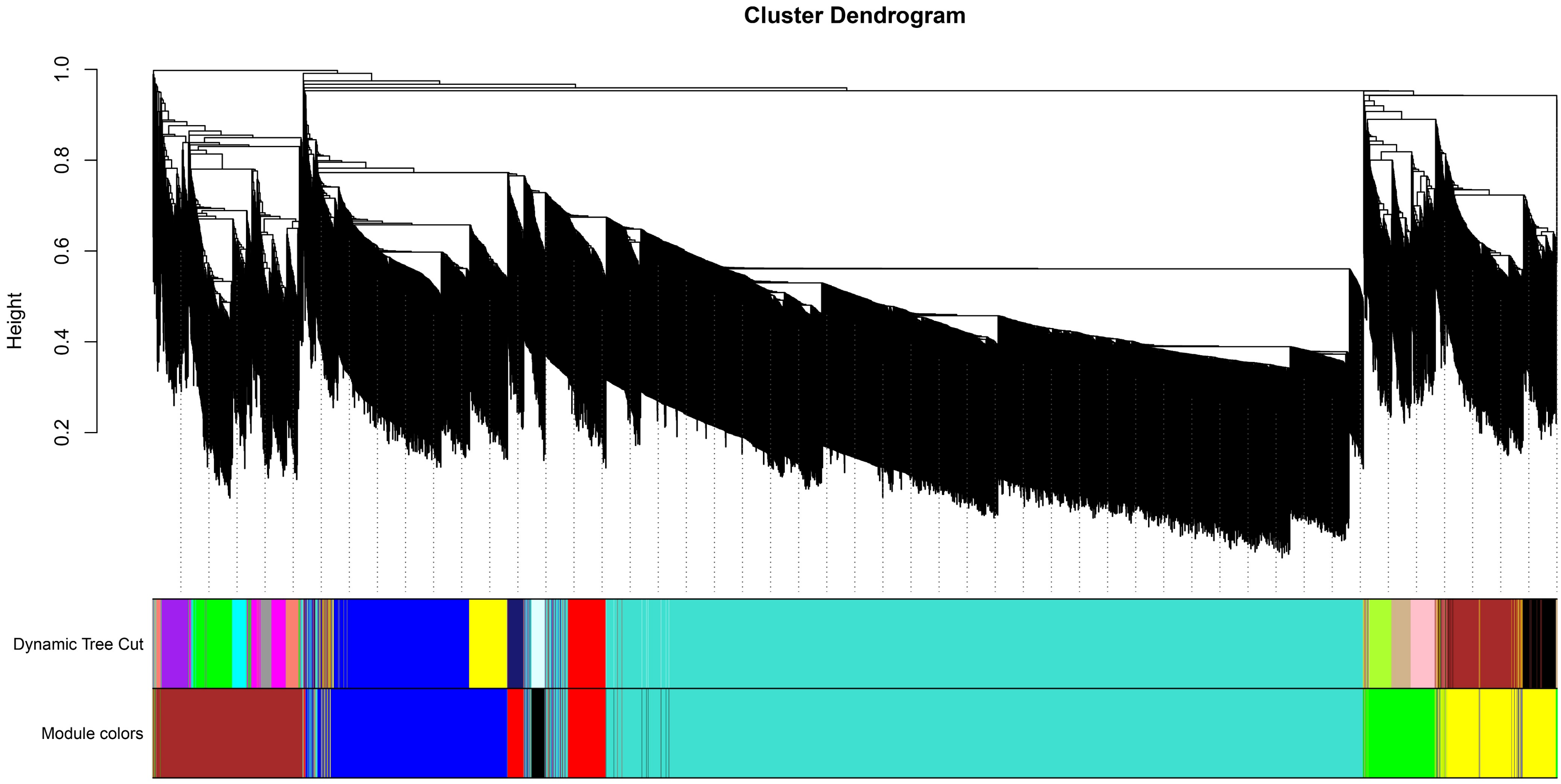

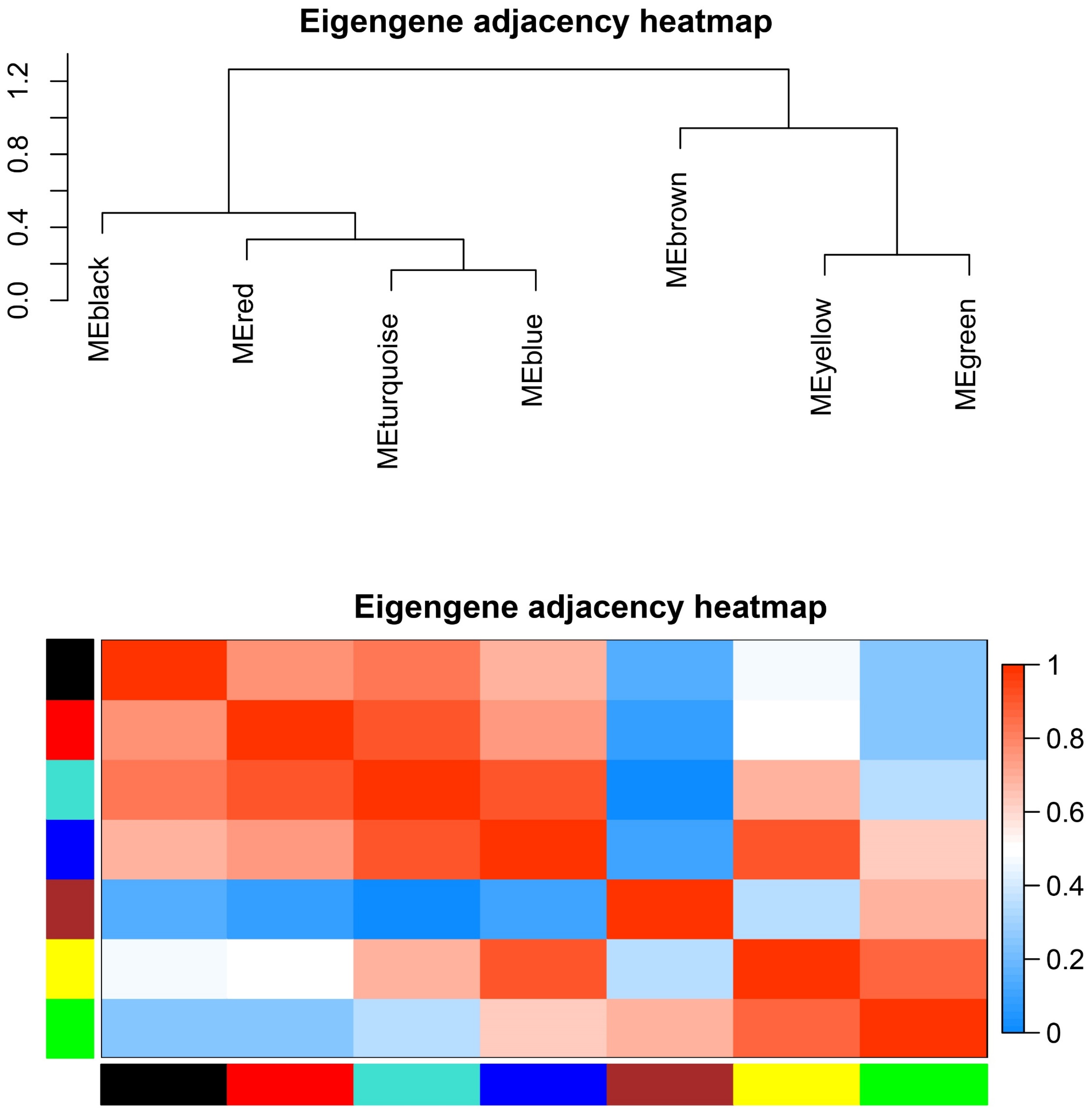

Eigengene adjacency heatmap
0.0
0.4
0.8
1.2
MEblack
MEred
MEturquoise
MEblue
MEbrown
MEyellow
MEgreen
Eigengene adjacency heatmap
1
0.8
0.6
0.4
0.2
0

Secondary metabolites

G4_VCm_Blue

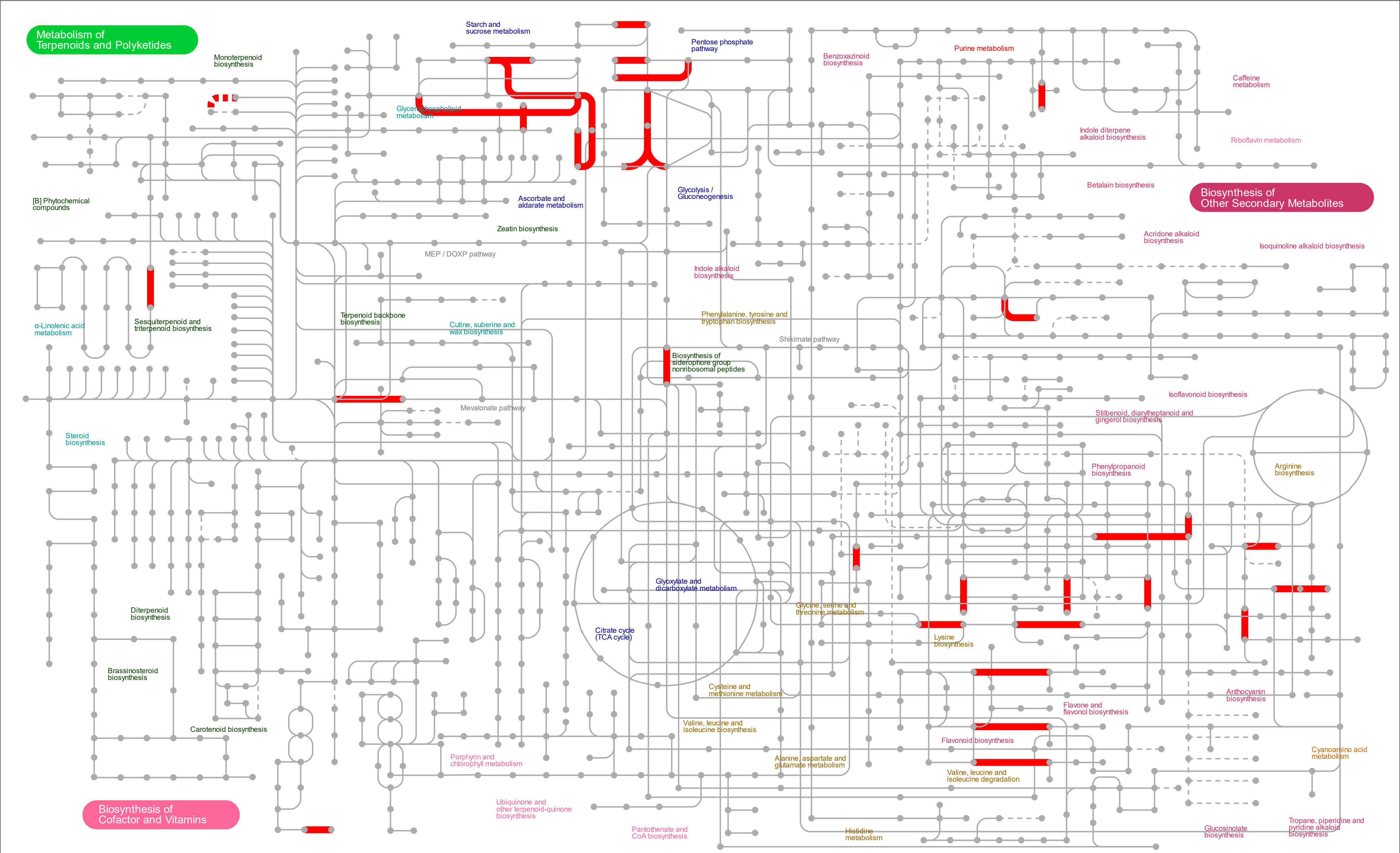

01110 4/21/17
(c) Kanehisa Laboratories

(D) Time Series analysis

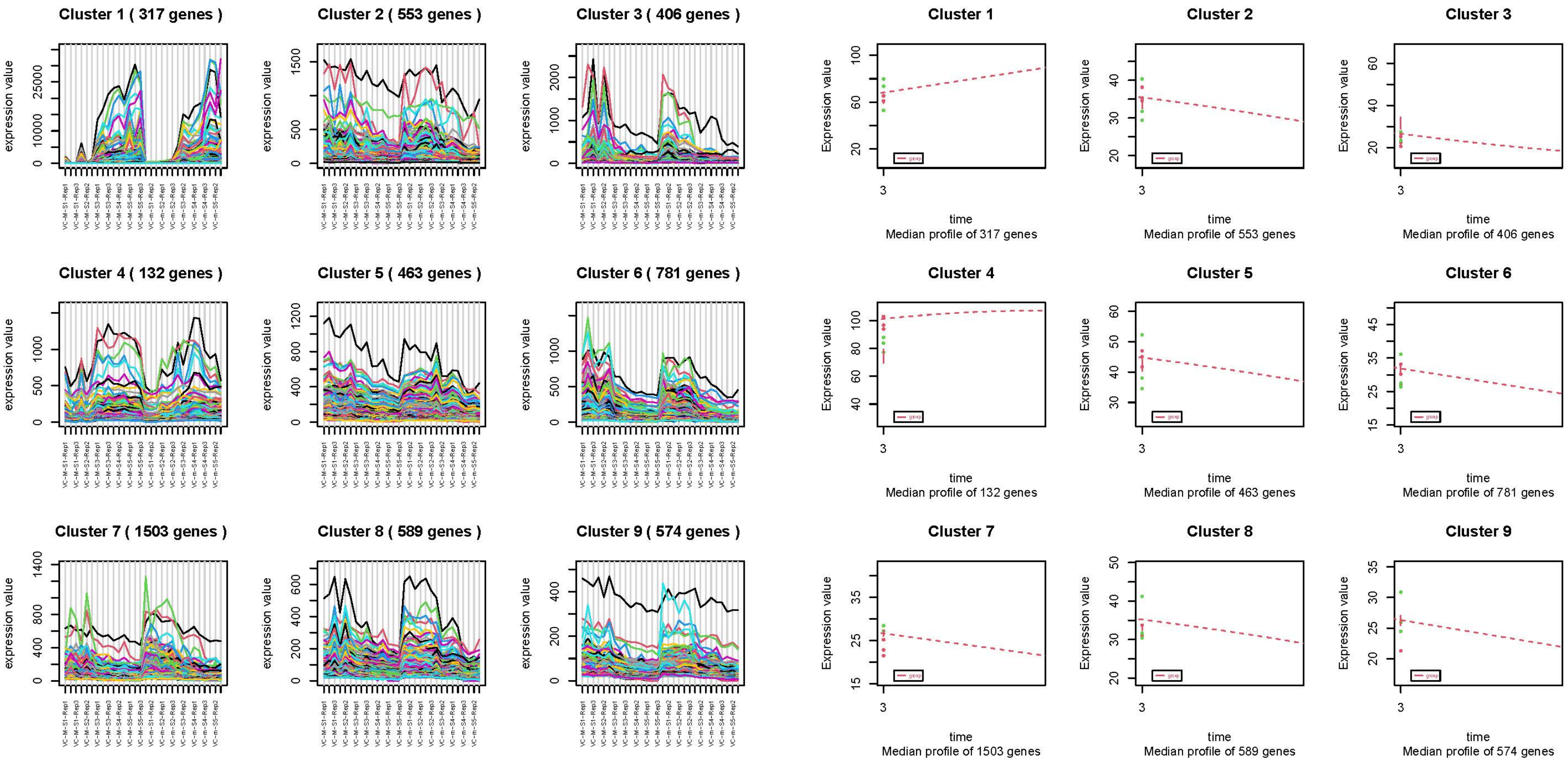

Supplement: Supplementary Figure 10 — Transcriptome analysis of G4 meta-data. [file DataSheet_10.pdf]
