# Supplementary material for: Developmental dynamic transcriptome and systematic analysis reveal the major genes underlying isoflavone accumulation in soybean
Source: Front Plant Sci. 2023 Mar 7;14:1014349. doi: 10.3389/fpls.2023.1014349 (PMC10027745; doi:10.3389/fpls.2023.1014349)
Supplement: Supplementary Figure 14 — GO annotation and enrichment analysis of Brown molecular module. [file DataSheet_14.pdf]

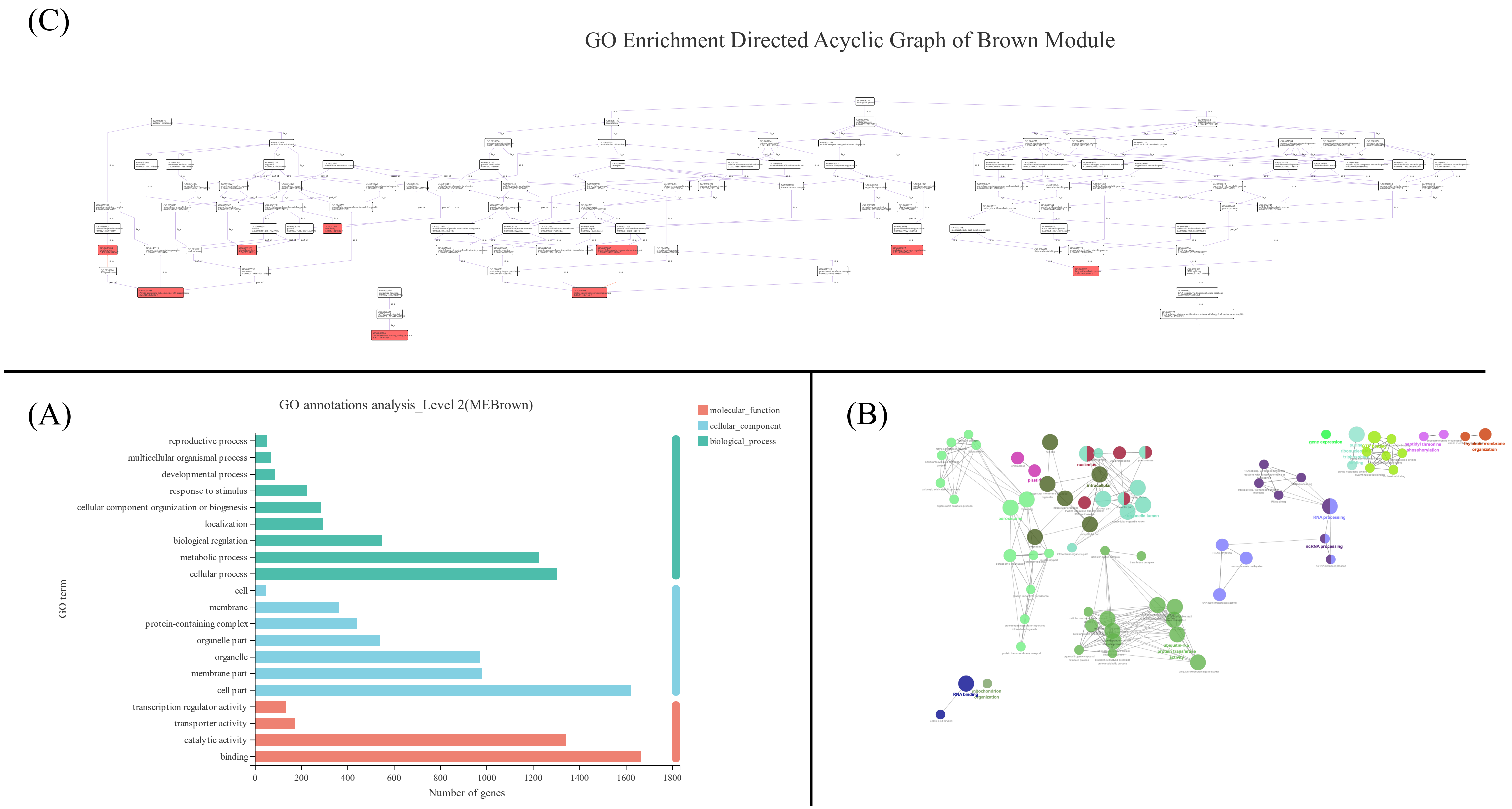

(A) GO Annotation

(B) GO Enrichment Network Analysis

(C) GO Enrichment Directed Acyclic Graph
